# Asthma characteristics and biomarkers from the Airways Disease Endotyping for Personalized Therapeutics (ADEPT) longitudinal profiling study

**DOI:** 10.1186/s12931-015-0299-y

**Published:** 2015-11-17

**Authors:** P. E. Silkoff, I. Strambu, M. Laviolette, D. Singh, J. M. FitzGerald, S. Lam, S. Kelsen, A. Eich, A. Ludwig-Sengpiel, G. C hupp, V. Backer, C. Porsbjerg, P. O. Girodet, P. Berger, R. Leigh, J. N. Kline, M. Dransfield, W. Calhoun, A. Hussaini, S. Khatri, P. Chanez, V. S. Susulic, E. S. Barnathan, M. Curran, A. M. Das, C. Brodmerkel, F. Baribaud, M. J. Loza

**Affiliations:** Janssen Research & Development LLC, 1400 McKean Rd, Springhouse, PA 19477 USA; Arensia Exploratory Medicine, Sos. Viilor 90, Bucharest, 050159 Romania; Institut Universitaire de Cardiologie et Pneumologie de Québec (IUCPQ), 2725, Chemin Ste-Foy, Québec, G1V 4G5 Canada; Medicines Evaluation Unit, University Hospital of South Manchester Foundation Trust, University of Manchester, Southmoor Road, Manchester, M23 9QZ UK; Institute for Heart and Lung Health, The Lung Centre, 7th Floor, Gordon, Canada; Leslie Diamond Health Care Centre, 2775 Laurel Street, Vancouver, BC V5Z 1M9 Canada; Department of Thoracic Medicine and Surgery, Temple University School of Medicine, 3401 N. Broad St., Philadelphia, PA 19140 USA; IKF Pneumologie Frankfurt, Institut für klinische Forschung Pneumologie, Clinical Research Centre Respiratory Diseases, Schaumainkai 101-103, Stresemannallee, 360596 Frankfurt, Germany; KLB Gesundheitsforschung Lübeck GmbH, Sandstr. 18, 23552 Lübeck, Germany; Yale Center for Asthma and Airway Disease, Division of Pulmonary and Critical Care and Sleep Medicine, Yale School of Medicine, TAC 441, 300 Cedar Street, New Haven, CT 06520 USA; Department of Respiratory Medicine, Respiratory Research Unit, Bispebjerg University Hospital, Bispebjerg bakke 23, DK-2400 Copenhagen, NV Denmark; Univ. Bordeaux, Centre de Recherche Cardio-Thoracique de Bordeaux, INSERM U1045, CIC 1401, F-33000 Bordeaux, France; Cumming School of Medicine, University of Calgary, 3280 Hospital Drive NW, Calgary, AB T2N 4Z6 Canada; Division of Pulmonary, C ritical Care, and Occupational Medicine, University of Iowa, W219B GH UIHC, 200 Hawkins Drive, Iowa City, IA 52242 USA; Division of Pulmonary, Allergy and Critical Care Medicine, University of Alabama at Birmingham & Birmingham VA Medical Center, 422 THT, 1900 University Blvd, Birmingham, AL 35294 USA; 4.116 John Sealy Annex, University of Texas Medical Branch, 301 University Blvd, Galveston, TX 77555-0568 USA; Parexel International, Shelton Simmons (MD), 3001 S Hanover St #7, Brooklyn, MD 21225 USA; Department of Pulmonary and Critical Care, Cleveland Clinic, 9500 Euclid Avenue, Cleveland, OH 44195 USA; Department of Respiratory Diseases and CIC Nord AP-HM, UMR INSERM U1067 CNRS 7733, Aix-Marseille Université, Marseille, France

**Keywords:** Asthma, Severity, Phenotypes, Profiling, Biomarkers, Personalized

## Abstract

**Background:**

Asthma is a heterogeneous disease and development of novel therapeutics requires an understanding of pathophysiologic phenotypes. The purpose of the ADEPT study was to correlate clinical features and biomarkers with molecular characteristics, by profiling asthma (NCT01274507). This report presents for the first time the study design, and characteristics of the recruited subjects.

**Methods:**

Patients with a range of asthma severity and healthy non-atopic controls were enrolled. The asthmatic subjects were followed for 12 months. Assessments included history, patient questionnaires, spirometry, airway hyper-responsiveness to methacholine, fractional exhaled nitric oxide (FENO), and biomarkers measured in induced sputum, blood, and bronchoscopy samples. All subjects underwent sputum induction and 30 subjects/cohort had bronchoscopy.

**Results:**

Mild (*n* = 52), moderate (*n* = 55), severe (*n* = 51) asthma cohorts and 30 healthy controls were enrolled from North America and Western Europe. Airflow obstruction, bronchodilator response and airways hyperresponsiveness increased with asthma severity, and severe asthma subjects had reduced forced vital capacity. Asthma control questionnaire-7 (ACQ7) scores worsened with asthma severity. In the asthmatics, mean values for all clinical and biomarker characteristics were stable over 12 months although individual variability was evident. FENO and blood eosinophils did not differ by asthma severity. Induced sputum eosinophils but not neutrophils were lower in mild compared to the moderate and severe asthma cohorts.

**Conclusions:**

The ADEPT study successfully enrolled asthmatics across a spectrum of severity and non-atopic controls. Clinical characteristics were related to asthma severity and in general asthma characteristics e.g. lung function, were stable over 12 months. Use of the ADEPT data should prove useful in defining biological phenotypes to facilitate personalized therapeutic approaches.

**Electronic supplementary material:**

The online version of this article (doi:10.1186/s12931-015-0299-y) contains supplementary material, which is available to authorized users.

## Background

Asthma is a highly-prevalent heterogeneous disease characterized by variable airflow obstruction with cough, dyspnea and wheezing. Patients are also at risk of exacerbations which may lead to hospitalization and in rare circumstances death. While there are many proposed asthma phenotypes, the underlying biology of these phenotypes remains poorly understood.

A subset of the asthma population has severe asthma, sometimes termed refractory asthma [1], which is incompletely responsive to currently available therapies. In combination with bronchodilators, the most effective therapies for asthma are anti-inflammatory in their action including most notably inhaled corticosteroids (ICS) and the monoclonal antibody (MAb) omalizumab (anti-IgE) [2]. Recently, MAb’s against interleukin- 4 (IL-4) receptor (IL-4R) [3], IL- 5 [4, 5], and IL-13 [6] have demonstrated efficacy by improving lung function and reducing exacerbations when administered in addition to standard therapies in severe asthma. Highlighting the importance of phenotyping, these therapeutics have enhanced efficacy in subjects selected using eosinophil- or type 2 immunity- associated markers e.g. serum periostin (POSTN), sputum and blood eosinophils (spEOS, bEOS), serum IgE (sIgE), and the fractional concentration of exhaled nitric oxide (FENO).

Airway inflammation, airflow obstruction, and airway hyper-responsiveness (AHR) represent major components of asthma pathophysiology [7]. Different inflammatory pathways may explain why most therapies are only effective in a subset of patients. Accordingly, diagnostic biomarkers are needed to appropriately classify patients and enable selection of a more targeted therapy for each phenotype. Such biomarkers are most easily assessed for clinical purposes in blood, exhaled breath, or urine. Other sampling methods such as induced sputum (IS) and bronchoscopy are only available in specialized centers. Response to ICS, for example, can be predicted by FENO [8], spEOS [9] and a 3-gene signature (CLCA1, POSTN, and serpinB2) in epithelial brushings [10].

The primary objective of the ADEPT study was to determine molecular and cellular profiles in peripheral blood, urine, induced sputum, and bronchial tissue across the spectrum of asthma severity and to enable the correlation of molecular subtyping with accessible clinical characteristics.

The goal of this report is to present for the first time the ADEPT asthma study design which enrolled asthma subjects of different severities and non-atopic healthy controls, and to describe subject characteristics and biomarkers.

## Methods

The study received ethical approval from the ethics committees of each of the sites involved. All subjects provided written informed consent to participate (genomic DNA testing was optional). The clinicaltrials.gov identifier is NCT01274507. The full study protocol is linked to this report.

### Population

Approximately 150 asthmatic subjects (50 subjects in each of 3 asthma categories (mild, moderate, severe) and 30 non-atopic healthy controls were planned for inclusion in the study. The National Heart, Lung, and Blood Institute (NHLBI) expert panel report [11] was adapted for classification of severity based on lung function and controller medication levels (Table 1). All subjects were non-smokers, or had quit for ≥ 1 year at initial screening visit and had a ≤ 10 pack-year history of smoking. A history of COPD or any other significant pulmonary disease was exclusionary. Imaging was not performed for this study, nor were prior imaging results collected.

**Table 1:** Categorization of asthma severity based upon background therapy and lung function

	Pre-BD FEV1 ≥ 80 % predicted normal	Pre-BD FEV1 ≥ 60- < 80 % predicted normal	Pre-BD FEV1 ≥ 50- < 60 % predicted normal	Pre-BD FEV1 < 50 % predicted normal
Controller medication				
None	Eligible for mild asthma cohort	Ineligible	Ineligible	Ineligible
Low to medium ICS alone or in combination with other controllers except chronic oral corticosteroids and omalizumab	Ineligible	Eligible for moderate asthma cohort	Ineligible	Ineligible
High dose ICS alone or in combination with other controllers including chronic oral corticosteroids and omalizumab	Ineligible	Eligible for severe asthma cohort	Eligible for severe asthma cohort	Ineligible

Mild asthma was defined as pre-bronchodilator forced expired volume in 1 s (FEV_1_) ≥ 80 % predicted values measured >6 h after the last use of bronchodilator and no asthma controller medication in the 6 weeks prior to screening, with short acting β-2 agonist (SABA) on an as-needed basis permitted (modified NHLBI STEP 1) [11]. This group allowed evaluation of biomarkers without the influence of corticosteroids.

Moderate persistent asthma was defined as pre-bronchodilator FEV_1_ from ≥ 60 to < 80 % predicted values measured > 6 h after the last use of any bronchodilator, treatment with SABA as needed, and low to medium dose ICS, based on NHLBI definitions [11], alone, or in combination with any other controller medication (e.g. long-acting β-2 agonists (LABA), leukotriene modifying agents, theophylline) with the exception of oral corticosteroids (OCS), or omalizumab (modified NHLBI STEP 2, 3, or 4;) [11].

Severe persistent asthma was defined as pre-bronchodilator FEV_1_ from ≥ 50 to <80 % predicted values measured > 6 h after last use of bronchodilator, treatment with SABA, and high dose ICS alone or in combination with any other controller medication including OCS, and/or omalizumab, (modified NHLBI STEP 5 or 6) [11].

### Inclusion criteria

All inclusion and exclusion criteria are listed in the study protocol (Additional file 1) linked to this report. Asthmatic subjects not participating in bronchoscopy were between 18 and 70 years old while those enrolled in the bronchoscopy cohort were 18–55 years old. Asthmatics were required to have principal investigator confirmation of a history of asthma with asthma symptoms for ≥6 months prior to screening, and exclusion of alternative diagnoses. There was no required level of asthma control, but subjects undergoing bronchoscopy had to be clinically stable for at least 6 weeks. Subjects had to meet at least 1 of the following three criteria tested sequentially:

1. Bronchodilator reversibility (BDR) defined as a FEV1 increase of at least 12 % and 200 mls after a SABA
  OR
2. The provocative concentration (PC20) of methacholine resulting in a 20 % or greater fall in the FEV1 < 16 mg/mL. PC20 could also have been documented within the previous 24 months. Assessment of PC20 required a pre-bronchodilator (pre-BD) FEV1 of at least 60 % predicted.
  OR
3. Airflow obstruction defined as a pre-BD FEV1/forced vital capacity (FVC) ratio <0.7 (for those with no BDR and unable to undergo PC20 testing due to low FEV1).

Healthy controls were 18–55 years old, non-smokers, and were required to be non-atopic based on a specificIgE panel, ImmunoCap Phadiatop™, (Phadia AB, Uppsala, Sweden). The healthy controls were required to have normal lung function (FEV1 % predicted of normal >85 %) and a BDR <12 % and <200 ml, Additionally, healthy controls were had no clinically significant abnormalities as determined by medical history, physical examination, blood chemistry assessments, hematologic assessments including complete blood count (CBC), urinalysis, measurement of vital signs, and ECG. Specifically, they were also required to have no history of allergic symptoms e.g., allergic rhinitis, or eczema.

## Study design and visits

### Asthma

**Fig. 1:**
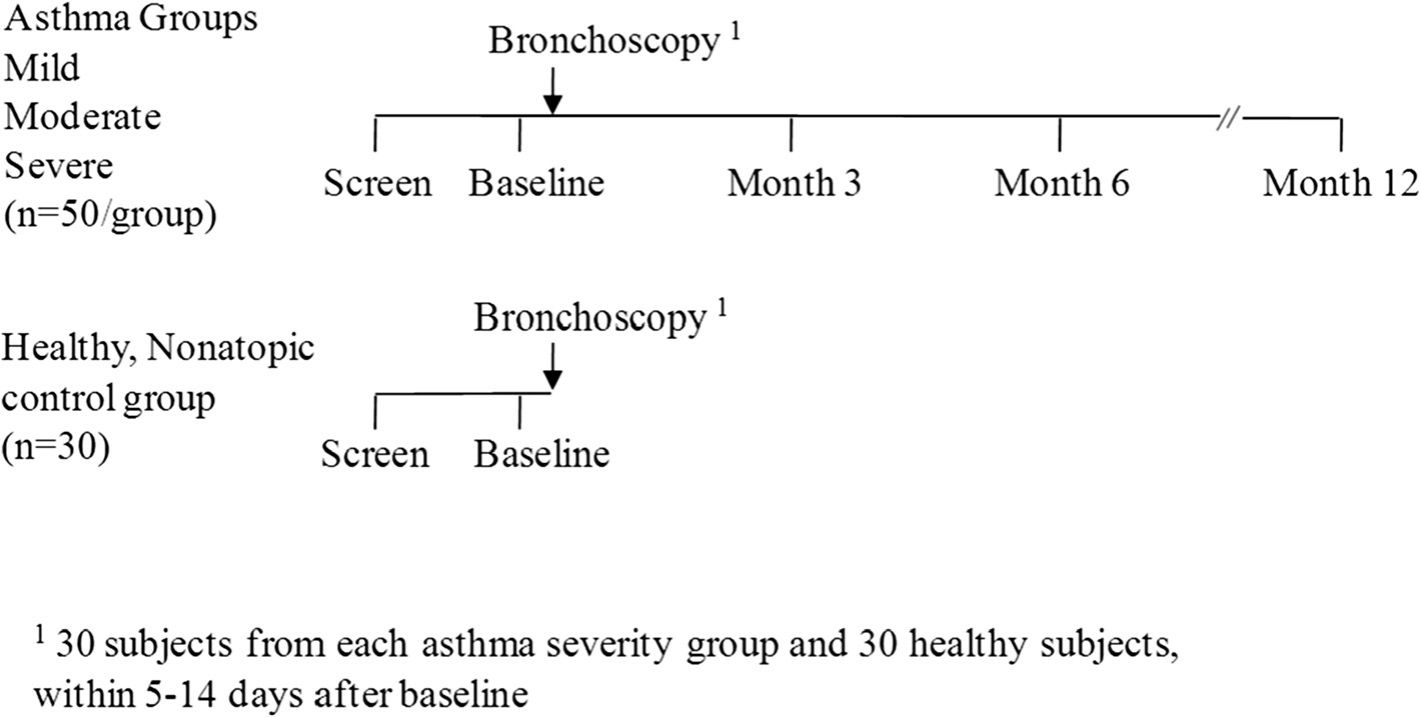
Planned study design and sample sizes for the healthy controls and asthmatics. The study was complete after the bronchoscopy visit for the healthy controls and after the 12 month biomarker visit for the asthmatic participants

A study design schematic is shown in Fig. 1. Asthmatic subjects underwent screening, then if enrolled attended the baseline visit. For subjects included in the bronchoscopy study, the bronchoscopy visit occurred within ~2 weeks of the baseline visit. Further clinical assessment/biomarker visits occurred at 3, 6 and 12 months, with induced sputum sampling repeated at the 6 month visit. These 3, 6 and 12 month visits were included to evaluate variability over time and also the impact of seasons.

The study procedures for asthmatic participants are detailed in the protocol linked to this report and were completed at several visits as necessary. The Screening Period included review of inclusion/exclusion criteria, obtaining consent, history and physical examination, vital signs, lung function testing, blood tests, and an acceptable sample of sputum obtained by induction was required from all study participants. PC20 methacholine testing was performed in asthmatics during screening if it was required for confirmation of asthma for inclusion into the study, provided prebronchodilator FEV1 was at least 60 % predicted. At the first visit after enrollment (i.e. the baseline visit) clinical and biomarker parameters, induced sputum and PC20 methacholine (in those who didn’t have this performed during screening) were assessed. The bronchoscopy visit was scheduled 5–14 days after the baseline visit to enable comparison of sputum and bronchoscopy samples within a short timeframe. Approximately 30 subjects of each asthma severity cohort underwent bronchoscopy to obtain endobronchial biopsy (EBBX) and epithelial brushing (EPBR) samples. All asthma subjects completed a detailed asthma history questionnaire, and kept a diary to record significant medical events and changes in medications.

### Healthy controls

The healthy controls underwent screening and then baseline visit procedures, and ended their participation in the study after the bronchoscopy visit. The study procedures for healthy controls are detailed in the protocol linked to this report. The Phadiatop™ (Thermo Fisher Scientific, Uppsala, Sweden) panel for specific IgE was performed during screening for eligibility purposes. Spirometry was assessed during screening and at the baseline and bronchoscopy visits before and after administration of SABA. Induced sputum was performed during screening and at the baseline visit and all healthy controls underwent bronchoscopy.

## Clinical assessments

### Study case report forms

The study coordinator captured data at screening and at each study visit using a electronic CRF which was purpose-built for ADEPT. The data the CRF captured included demographic characteristics, medical history, and 4 questions about asthma: 1) asthma date of diagnosis 2) number of asthma-related ER visits in the last year 3) number of asthma-related hospitalizations in the last year and 4) number of asthma-related episodes requiring additional treatment with systemic corticosteroids (IV or oral) in the last year.

### Asthmatics

Patient reported outcomes, assessed repeatedly throughout the study, included the 7-item asthma control questionnaire (ACQ7) [12] and the asthma quality of life questionnaire (AQLQ) [13]. A history of recent exacerbations was documented during screening. Spirometry was performed according to American Thoracic Society/European Respiratory Society (ATS/ERS) guidelines [14] with predicted values based on NHANES III [15]. BDR was defined as the change in FEV1 15–30 min after administration of a SABA and assessed during screening, and at the baseline, 3, 6, and 12 month visits. Each site used their own spirometry equipment that conformed to ERS/ATS standards [14]. Methacholine PC20 was performed during screening if required to confirm a diagnosis of asthma, or at the baseline visit (only if the pre-bronchodilator FEV1 was ≥60 % predicted). FENO was measured at an exhalation flow rate of 50 ml/s [16] at baseline and was repeated at all visits. In addition to the Case Report Form (CRF)-captured variables above, an asthma history questionnaire (AHQ) was developed which surveyed relevant details concerning each subject’s asthma history and was used for the first time in this protocol for exploratory evaluations. Each subject completed the AHQ independently, and the results were captured into the electronic CRF.

## Biomarker assessments

### Bronchoscopic sampling

Bronchoscopy was performed in a standardized fashion. Up to 6 adequate endobronchial biopsies (based on visual inspection) were taken first, with no more than 8 attempts at biopsy, if the subject was tolerating the procedure well. The biopsies were taken at bifurcations of sub-segmental airways in the lower lobe on one side. The biopsy samples were processed immediately for the required analyses using the following sequence: 2 biopsy samples for ribonucleic acid (RNA) isolation were be placed in RNAlater medium (Qiagen Inc., Valencia, CA) then 2 biopsy samples for histology-immunostaining were be placed into formalin, followed by the last 2 biopsies for RNA isolation in RNAlater. Bronchial brushings were performed after the collection of the endobronchial biopsies. During the brushing procedure, a small cytology brush was passed through the bronchoscope in the lung opposite to that in which biopsies were taken, and a small area of the airway wall was brushed gently to obtain epithelial cells lining the airway. Brushing was performed up to 6 times depending on the patient’s tolerance of the procedure. Each brush was passed into the airways and then passed back and forth into sub-segmental airways of the lower lobe 3–5 times.

### Induced sputum (IS)

#### Induction procedure

All study participants underwent sputum induction during screening to fulfill inclusion criteria and again at the baseline visit. Asthmatic subjects only had a 3^rd^ sputum induction at the 6-month biomarker visit. Sputum was induced for 21 min divided into three 7-min sessions of nebulization each followed by a 3 step cleansing procedure and a focused cough attempt. An aerosol of hypertonic saline (in increasing concentrations of 3, 4, and 5 %) was generated by an ultrasonic nebulizer for inhalation by subjects with a post-BD pre-induction FEV_1_ of ≥60 % predicted; for those with FEV_1_ ≥ 50- < 60 % predicted, induction was performed with normal/isotonic saline (0.9 %). Subjects with a post-BD FEV_1_ < 50 % predicted did not undergo induction.

#### Sputum processing

The plug selection method with dithiothreitol dispersal of mucus was used for this study in all participants [17]. At the screening visit, a plug weight of at least 50 mg and squamous cell percentages ≤ 20 % were required for enrollment. Sputum supernatant and slides for differential cell count were prepared.

Only samples with squamous cell content ≤20 %, based on each site’s evaluation, were included in the analyses.

### Serum analytical methods

Serum was collected using standard serum separation tubes, and frozen within 30 min. One of these frozen aliquots, without intermediate freeze-thaw cycles, was provided for quantification of 1129 serum analytes using the SomaScan v3 platform (SomaLogic, Boulder, CO; www.somalogic.com). Serum analyte levels were reported by Somalogic as relative fluorescence units, cross-plate calibrated, and median normalized. Analyte levels are presented as the log_2_ ratio to the geometric means of the healthy control population for further analysis. Results for serum total immunoglobulin E (sIgE) are presented from this panel, defining high sIgE levels as those above the 95^th^ percentile of the healthy control distribution. In previous evaluations of the platform in asthmatics and healthy controls, sIgE measurements highly correlated (Pearson’s correlation coefficient *r* > 0.9) with those obtained from standard ELISA-based assays (data not shown).

### Safety

Investigators were instructed to capture adverse events (AE) that were attributed to study procedures e.g. induced sputum, bronchoscopy, etc. Emergent AEs that were unrelated to procedures were captured at the investigators’ discretion.

### Statistical considerations

Statistical analyses of clinical and biomarker data used SAS v9 (Cary, NC) and OmicSoft ArrayStudio v7 (Cary, NC; www.omicsoft.com). Stability of parameters over time used a mixed linear model. No imputation was performed for missing data. For data with log-normal distributions (e.g., FENO, blood differential counts, serum protein measurements), logarithm transformations were performed. Significance of differences among groups was evaluated using General Linear Model analyses. *P*-values <0.05 were considered to be statistically significant. Correlations among variables were tested using Spearman correlation tests that do not require assumptions of normality and linearity. Because of the large number of pair-wise comparisons for the correlation analysis, a significance threshold of *p* < 0.0004 was established by the Bonferroni adjustment method to maintain a family-wise error rate <0.05 for the 136 pair-wise comparisons of 16 variables.

## Results

### Disposition

The study enrolled 30 healthy controls and 52 mild, 55 moderate, and 51 severe asthma subjects in the USA, Canada, Romania, Denmark, Germany, France and the United Kingdom between the years 2010–2013 at 17 sites that were selected for their established experience in asthma research, including ability to perform spirometry and other study procedures with requisite quality, and supervision by the Sponsor. The breakdown by country/region is shown in Table 1 with approximately equal proportions derived from the USA, Canada, EU (excluding Romania) and Romania. Thirty subjects in each cohort underwent bronchoscopy. All healthy control subjects completed the study and 17 of 158 asthma subjects withdrew prematurely (1 for a non-serious AE; 5 withdrew consent, 2 for pregnancy, 4 for sponsor decisions, 2 were lost to follow-up and 3 withdrew for other reasons).

### Demographic and clinical history from Case Report Form (CRF)

#### Demographic characteristics

**Fig. 2:**
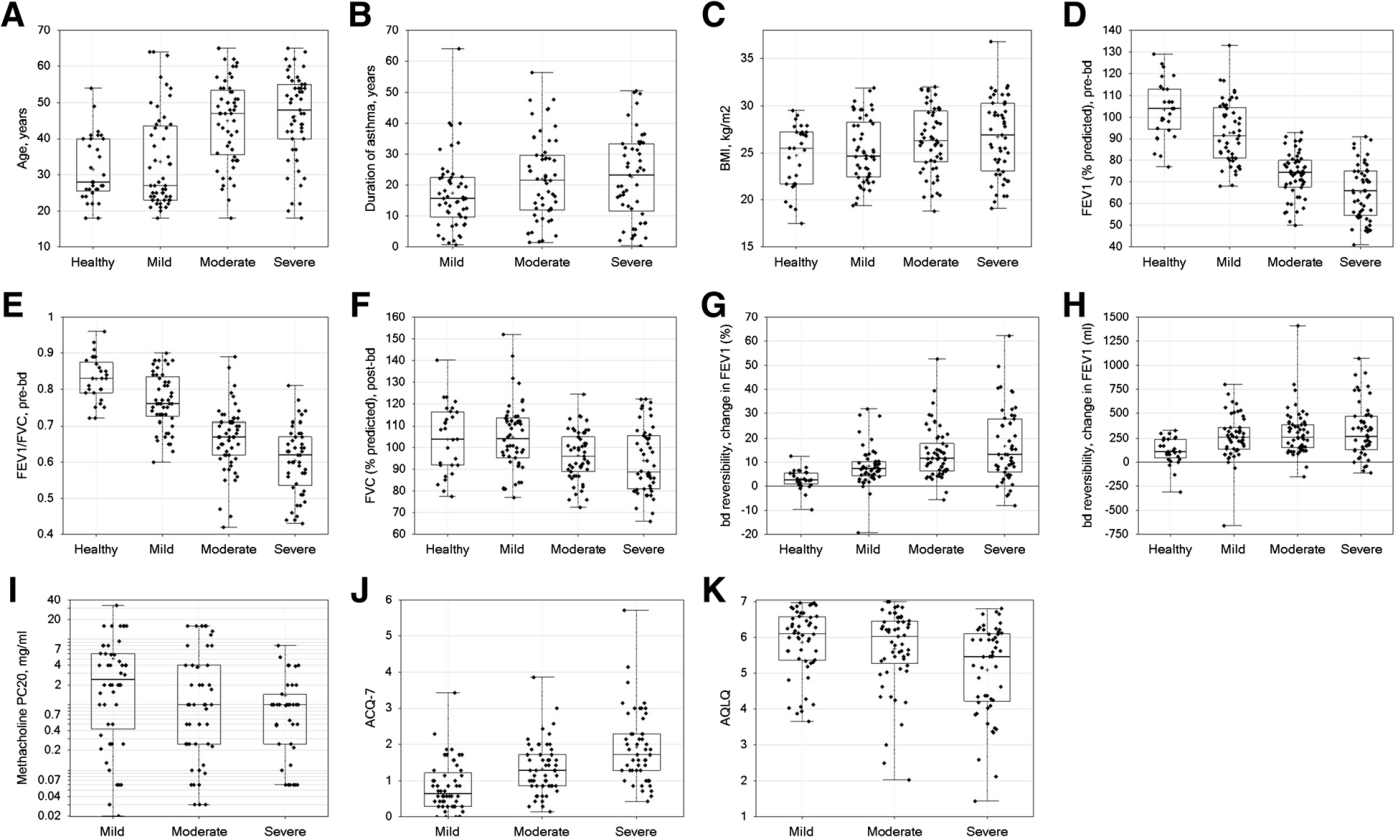
Demographic and clinical characteristics. The values for the indicated variables (y-axis) are shown for healthy control and asthma severity groups (x-axis). Data presented as symbols representing individual subjects and summarized by box (inter-quartile range and median) & whiskers (range), with ‘+’ indicating mean. Significance of differences among groups is reported in Tables 2 (for panels **a**-**c**) and 4 (for panels **d**-**k**)

Detailed demographic characteristics are shown in Table 2 by cohort and in Fig. 2 (panels a-c). Mild asthmatics were younger than moderate and severe asthmatics (mean ages 33.7, 45.0 and 46.2 years, respectively; *p* < 0.05), but similar to healthy controls (mean age 31.6 years). Participants who underwent bronchoscopy were approximately 10 years younger than those not participating in this procedure for all severity cohorts (data not shown), in part due to restriction of their upper age to 55 years by protocol. The mean duration of asthma tended to be shorter in the mild asthma cohort compared to the moderate and severe asthmatics (*p* = 0.21 and 0.22, respectively). There was slight female predominance in each asthma cohort, but male predominance in the healthy control cohort. Mean body mass index (BMI), which was limited by the protocol to <32 kg/m^2^ (ranging from 24.6 to 26.7 kg/m^2^) was modestly higher in moderate and severe asthma cohorts compared to healthy controls (*p* < 0.05 for each comparison) and tended to rise with severity (*p* = 0.094 across asthma severity cohorts, from 24.6 to 26.7 kg/m^2^). Obesity, defined by BMI > 30 kg/m^2^, increased with asthma severity (*p* = 0.008, Armitage Test for Trend in Proportions), from 8, 16, and 27 % of mild, moderate, and severe asthma cohorts, respectively.

**Table 2:** Demographic characteristics

Cohort	Healthy Controls	Mild	Moderate	Severe	Total
No. Subjects	30	52	55	51	188
USA	3 (10 %)	11 (21 %)	15 (27 %)	12 (24 %)	41 (22 %)
Canada	12 (39 %)	10 (19 %)	12 (22 %)	17 (33 %)	51 (27 %)
EU, ex-Romania	9 (29 %)	21 (40 %)	17 (31 %)	11 (22 %)	58 (31 %)
Romania	7 (23 %)	10 (19 %)	11 (20 %)	11 (22 %)	39 (21 %)
Cohort	Healthy Controls	Mild	Moderate	Severe	P-value *
Age (yrs)					<0.0001/<0.0001
Mean (SD)	31.6 (9.2)	33.7 (13.1)	45.0 (11.6)	46.2 (12.1)	
Range	(18; 54)	(18; 64)	(18; 65)	(18; 65)	
Male Sex	19 (63.3 %)	20 (38.5 %)	27 (49.1 %)	23 (45.1 %)	0.14/0.54
Race					0.43/0.27
White	27 (90.0 %)	46 (88.5 %)	47 (85.5 %)	41 (80.4 %)	
Black or African American	1 (3.3 %)	1 (1.9 %)	5 (9.1 %)	7 (13.7 %)	
Asian	2 (6.7 %)	4 (7.7 %)	3 (5.5 %)	2 (3.9 %)	
Other	0	1 (1.9 %)	0	1 (2.0 %)	
BMI (kg/m^2^)					0.034 /0.094
Mean (SD)	24.6 (3.4)	25.2 (3.4)	26.4 (3.5)	26.7 (4.1)	
Range	(17.5; 29.5)	(19.4; 31.9)	(18.8; 32.0)	(19.1; 36.8)	
Duration asthma (yrs)					NA/0.094
Mean (SD)	NA	17.4 (11.9)	22.0 (13.6)	22.7 (14. 0)	
Range	NA	(0.7; 64.0)	(1.5; 56.3)	(0.3; 50.5)	

*p-value (ANOVA F-test, or Fisher’s exact test when N, % reported) for differences across severity cohorts, including/excluding healthy control cohort

#### Assessments for allergy

A specific IgE panel (Phadiatop), assessed during screening, was negative in all healthy controls by protocol, but positive in a vast majority of asthmatics regardless of severity (76, 80, and 76 % for mild, moderate, and severe cohorts, respectively). Correspondingly, almost all asthmatics had a history of allergies, determined from the AHQ (90, 87, and 88 % for mild, moderate, and severe cohorts, respectively). For Phadiatop positive subjects (*n* = 120), 93.3 % reported a history of allergy and 87 % had high sIgE. For Phadiatop negative subjects (*n* = 35), 25 of 35 (71 %) still reported a history of allergies and 16 of 35 (46 %) had high sIgE.

#### Prior history of asthma exacerbations

The mean number of asthma exacerbations requiring systemic steroids in the 12 months prior to the study reported by subjects to the study coordinators and captured in the case report form (CRF) was 0.3 for the mild and moderate asthmatics cohorts, and 0.5 for the severe cohort. The proportion of asthmatics having at least one exacerbation in the previous year was 4 % of mild, 15 % of moderate, and 27 % of severe asthma cohorts. Thus, in general, the ADEPT asthma cohort was not highly enriched for exacerbation propensity.

#### Medical history

The prevalence of common co-morbidities reported by the asthmatic participants during screening derived from the screening CRF were as follows in the mild, moderate, and severe severity cohorts, respectively: sinusitis (25.0, 25.5 and 27.5 %), allergic rhinitis (63.5, 50.9, and 51.0 %); gastro-esophageal reflux (11.5, 10.9, and 23.5 %); anxiety (7.7, 5.5, and 9.8 %); depression (3.8, 14.5, and 7.8 %). Other common medical conditions e.g. diabetes, hypertension were rare probably due to exclusion criteria.

The reported medical history for the healthy controls in the CRF was as follows: sinusitis (*n* = 1), migraine (*n* = 2), depression (*n* = 1) and other conditions not detailed (*n* = 3).

### Asthma History Questionnaire (AHQ)

The AHQ is a non-validated patient-reported outcome questionnaire that was developed for ADEPT, and this was the first time it was included in a study. A table of results from the AHQ is provided online as Additional file 2. Data from the asthma history questionnaire in some instances revealed discrepancies with the data collected by the study coordinators via the CRF. Reported p-values for AHQ are uncorrected for multiplicity of testing and associations should be considered exploratory.

#### Common conditions accompanying asthma (AHQ)

Approximately 50 % of asthmatics reported having a first degree relative(s) with asthma. Moderate and severe asthmatics reported more frequently adult onset of asthma and lung infections deemed serious by the patient than mild asthmatics. The majority of asthma subjects reported seasonal nasal allergies with a greater proportion of mild asthmatics (78.8 %) compared to moderate and severe asthmatics (63.6, 62.7-%, respectively) (*p* = 0.079 for decreasing trend with severity). Perennial nasal allergies were reported by ~30 % of asthmatics with no relationship to severity. Nasal polyps were reported to a far greater degree by severe asthmatics (29.4 %) compared to 18.2 % in moderate and 3.8 % mild asthmatics (*p* < 0.0001 for increasing trend with severity). Symptoms of gastroesophageal reflux, which have been associated with asthma, were reported by ~35 % of asthmatics unrelated to severity. Although BMI was restricted to <32Kg/m^2^, severe asthmatics reported obesity to a greater extent (13.2 %) compared to moderate (7.3 %) and mild (3.8 %) asthma (*p* = 0.069 for increasing trend with severity). Anxiety and depression were reported by 24.7 % of asthmatics unrelated to severity.

#### Asthma symptoms (AHQ)

Asthma symptoms present in the vast majority of study participants during periods of worsening asthma included shortness of breath, chest tightness, cough, and audible wheezing. Expectoration of mucus during asthma worsening was more common in moderate and severe asthma (60.0, 60.8 %, respectively) compared to mild asthma (44.2 %) (*p* = 0.091 for increasing trend with severity). Nocturnal asthma was reported to a greater degree by moderate and severe asthmatics (81.8 and 86.3 %, respectively) compared to mild asthma (65.4 %) (*p* = 0.011 for increasing trend with severity).

#### Asthma triggers (AHQ)

The following triggers were reported by a majority of asthmatic subjects and were unrelated to asthma severity: seasonal allergens, house dust, cold air, viral infections, and exercise. The following triggers were reported by a majority of asthmatic subjects but *were* related to asthma severity: weather conditions: moderate (56.4 %) and severe (58.8 %) asthma compared to mild (38.5 %) (*p* = 0.039 for increasing trend with severity); irritant exposure e.g. tobacco smoke etc.: moderate (70.9 %) and severe (78.4 %) asthma compared to mild (63.5 %) (*p* = 0.096 for increasing trend with severity).

#### Smoking history (AHQ)

All subjects had to be current nonsmokers with a <10 pack year history, and confirmation was sought with urinary cotinine. 32.7 % of mild, 43.6 % of moderate and 39.2 % of severe asthmatics reported previous use of tobacco products, for the most part cigarettes. The number of cigarettes smoked per day was mostly in the 0–5 range for mild asthmatics and the 6–10 range for moderate and severe asthmatics. Second-hand smoke exposure at home was reported by 69.6 % of asthmatics unrelated to severity. Only 16.4 % of asthmatics were still exposed to tobacco smoke at home and most subjects rated this exposure as light. Reported secondhand smoke exposure in the workplace in the past increased with severity (*p* = 0.014).

#### Treatments for asthma (AHQ)

SABAs were used by 89.9 % of asthmatics unrelated to severity. Mild asthmatics were not on controller medications at the time of enrollment by protocol but daily controller medications (current or prior) were reported by 44.2 % of mild, 98.2 % of moderate and 94.1 % of severe asthmatics. Allergen immunotherapy was reported by 9.6 % of mild, 10.9 % of moderate and 15.7 % of severe asthmatics. Omalizumab was used by 13.7 % of severe asthmatics only. Adherence with controller medications was reported by 44.0 % of mild, 90.9 % of moderate and 100 % of severe asthmatics. For the mild asthmatics who were on no controller medications at the time of enrollment, the reported adherence presumably refers to prior use of controller therapy.

#### Exacerbations (AHQ)

The asthma subjects reported the frequency of “increase in medications for asthma worsening” which could represent moderate exacerbations, as well as severe-OCS-defined exacerbation. Both mild and moderate asthma had similar proportion of subjects with ≥1 (46 and 42 %) and ≥2 (27 and 26 %) events per year, compared to severe asthma which had more events (60 % for ≥1 and 50 % for ≥2 per year; *p* = 0.13 and 0.0088, respectively).

### Asthma disease characteristics at baseline

Table 3 reports asthma disease characteristics at the baseline visit only (screening or baseline visit for PC20), as mean parameters did not change over the 12 months of the study.

**Table 3:** Asthma disease characteristics by cohort at Baseline Visit (Screening or Baseline for PC20)

Cohorts	Healthy	Mild	Moderate	Severe	P-value***
N	30	52	55	51	
Pre-BD FEV1 (L)	3.98 (0.81) *	3.35 (0.81)	2.39 (0.62)	2.10 (0.71)	<0.0001
Pre-BD FEV1 (% predicted normal)	103.3 (13.4)	92.7 (14.3)	73.6 (10.4)	65.4 (12.7)	<0.0001
Post-BD FEV1 (L)	4.10 (0.84)	3.61 (0.87)	2.67 (0.66)	2.43 (0.81)	<0.0001
Post-BD FEV1 (% predicted normal)	106.2 (13.7)	101.4 (14.0)	82.7 (10.2)	75.7 (15.4)	<0.0001
Pre-BD FEV/FVC ratio	0.83 (0.06)	0.77 (0.08)	0.66 (0.09)	0.61 (0.09)	<0.0001
Post-BD FVC (% predicted normal)	103.8 (15.9)	105.0 (15.5)	96.4 (11.4)	94.0 (15.1)	0.0004
BDR (%)	2.9 (4.1)	8.5 (8.3)	15.2 (10.3)	18.3 (14.5)	0.0016
BDR (mL)	114.8 (140.6)	265.1 (231.7)	335.2 (234.3)	355.7 (270.6)	0.45
PC20 methacholine (mg/mL)	NA	1.68 (+10.85/-0.26)**	0.93 (+6.49/-0.13)**	0.63 (+2.62/-0.15)**	0.034
ACQ7	NA	0.84 (0.69)	1.33 (0.71)	1.92 (1.01)	<0.0001
Controlled ACQ <0.75 (N, %)	NA	29 (56 %)	10 (18 %)	4 (8 %)	
Partially controlled ACQ 0.75-1.5 (N, %)	NA	13 (25 %)	24 (44 %)	16 (31 %)	<0.0001
Uncontrolled ACQ ≥ 1.5 (N, %)	NA	10 (19 %)	21 (38 %)	31 (61 %)	
AQLQ	NA	5.86 (0.93)	5.68 (1.11)	5.09 (1.28)	0.0016

*Mean (standard deviation) reported by cohort, unless otherwise indicated ** Geometric mean (asymmetric standard deviation) *** p-value (ANOVA F-test, or Fisher’s exact test when N, % reported) for differences across severity cohorts, excluding healthy control cohort (based on log-transformed data when geometric means reported)

#### Lung function

These data are presented in Fig. 2 (panels d-h) and Table 3. The inclusion criteria were responsible in part for the increasing severity of airflow limitation from mild to severe asthma. Mean % predicted pre-BD FEV1 declined across mild to moderate and severe asthma (92.7, 73.6, and 65.4 %, respectively; *p* < 0.0001); FEV1/FVC ratio showed a similar pattern, with 20, 61, and 82 %, respectively, of subjects having a pre-BD FEV1/FVC ratio below 0.7. Mean post-BD FVC also fell from mild to severe asthma (105.0, 96.4, and 94.0 % predicted, respectively; *p* = 0.0004), with 2, 9, and 22 % of mild, moderate, and severe asthmatics having a post-BD FVC of 80 % predicted normal or below, possibly reflecting air trapping. Mean BDR increased across mild to severe asthma (8.7, 15.2, and 18.3 %, respectively; *p* = 0.0016). In general, lung function was slightly better in bronchoscopy subjects compared to non-bronchoscopy subjects (data not shown). A similar pattern was observed at months 3, 6 and 12 (data not shown). There was no statistically significant change in mean spirometric measures over time for each asthma severity cohort (data not shown).

#### Methacholine PC20

PC20 (geometric mean, mg/mL) varied among the asthma cohorts (*p* = 0.034), trending to be lower in the severe asthma cohort (0.68 mg/ml) compared to the mild (1.68 mg/ml, *p* = 0.12) and moderate (0.93 mg/ml, *p* = 0.062) asthma cohorts (Fig. 2 panel i and Table 3). Methacholine PC20 was not evaluated longitudinally.

#### Patient reported outcomes

Data are shown in Fig. 2 panels j-k and Table 3. ACQ7 scores can range from 0: excellent control to 7: extremely uncontrolled [12]. At the Baseline Visit, ACQ7 scores (mean; range) rose significantly with asthma severity (*p* < 0.0001): mild (0.84; 0.00–3.4), moderate (1.33; 0.1–3.9) and severe asthma (1.92; 0.4–5.7) indicating that on average, subjects with mild asthma were well-controlled, moderate asthma were partially- controlled and most subjects with severe asthma were uncontrolled. The proportions of patients with well-controlled, partially controlled, and uncontrolled asthma based on ACQ rose significantly by the severity of the asthma cohorts (*p* < 0.0001) as shown in Table 3.

AQLQ scores can range from 1: severe impairment to 7: no impairment [13]. For the baseline visit, AQLQ (mean; range) fell significantly with increasing asthma severity: mild (5.86; 3.7–7.0), moderate (5.68; 2.0–7.0) and severe asthma (5.09; 1.4–6.8) but the proportions of subjects with AQLQ scores under 5.0 rose with asthma severity (*p* = 0.0016).

A similar pattern was observed at months 3, 6 and 12 for ACQ and AQLQ (data not shown). There was no statistically significant change in mean values of ACQ and AQLQ over time for each asthma severity cohort (data not shown).

### Impact of region on clinical variables

Region (US, Canada, EU-ex Romania, and Romania) had minimal impact on the main demographic and clinical variables reported in Tables 2 and 3. For 2 variables (pre-BD FVC, lower in mild asthmatics from Canada; and PC20, trend for lower values in Romania), there were nominally significant interactions (*p* < 0.05) between region and asthma severity cohort that did not pass multiple testing corrections with FDR < 0.05. The significance of associations of these 2 variables with asthma severity cohorts was also maintained after adjustment for region.

### Safety

#### All adverse events

Mild asthmatics had less AEs compared to moderate and severe asthmatic subjects, and subjects participating in bronchoscopy had more AEs than those not having bronchoscopy. The % of subjects who reported at least 1 AE were as follows: healthy controls who were all bronchoscopy participants (10.0 %), mild asthmatics: bronchoscopy (26.7 %), and non-bronchoscopy participants (13.6 %), moderate asthmatics: bronchoscopy (46.7 %), and non-bronchoscopy participants (24.0 %), and severe asthmatics: bronchoscopy (36.7 %), and non-bronchoscopy participants (33.3 %). SAEs occurred in 4 subjects and were all unrelated to study procedures. Preferred terms included flank pain (*n* = 1), adenocarcinoma of the colon (*n* = 1), trigeminal neuralgia (*n* = 1), pleural effusion (*n* = 1), and deep venous thrombosis (*n* = 2), with multiple SAEs in 1 subject.

#### Procedure-attributed adverse events

There were no procedure-related SAEs. Subjects participating in bronchoscopy had more AEs than those not having bronchoscopy. The percentage of subjects who reported at least 1 AE were as follows: healthy controls who were all bronchoscopy participants (10.0 %), mild asthmatics: bronchoscopy (16.7 %), and non-bronchoscopy participants (9.1 %), moderate asthmatics: bronchoscopy (40.0 %), and non-bronchoscopy participants (8.0 %), and severe asthmatics: bronchoscopy (26.7 %), and non-bronchoscopy participants (9.5 %).

In general, the respiratory and mediastinal system-organ class was the commonest class contributing to procedure-related AEs with a much higher incidence in those undergoing bronchoscopy as follows: healthy controls (3.3 %), mild asthma (9.1 %), moderate asthma (26.7 %), and severe asthma (20.0 %). Asthma was the commonest AE occurring in 0 mild, 3.6 moderate, and 5.9 % severe asthmatics, followed in frequency by bronchospasm and cough. Hemoptysis had a low incidence (1 healthy control and 1 mild asthmatic subject).

### Clinical biomarkers

Clinical biomarkers are presented in

**Table 4:** Clinical biomarkers and sputum differential counts by cohort at Screening/Baseline Visits

Cohorts	Healthy	Mild	Moderate	Severe	p-value***
n (blood/sputum)	30/20	52/32	55/38	51/40	
FENO (ppb)*	NA	32.9 (+64.2/-16.9)	29.1 (+61.0/-13.9)	28.8 (+64.7/-12.9)	NA/0.59
bEOS, cells/μl*	112 (+218/-57)	178 (+347/-91)	197 (+435/-89)	210 (+452/-97)	0.0018/0.54
bNEU, 1000 cells/μl*	3.47 (+4.96/-2.42)	3.59 (+4.72/-2.74)	3.64 (+4.92/-2.69)	3.94 (+5.89/-2.63)	0.35/0.32
Serum IgE, RFU*	1.0 (+2.1/-0.7)	8.3 (+25.3/-6.2)	9.8 (+26.7/-7.2)	12.1 (+31.0/-8.7)	<0.0001/0.35
Sputum eosinophils, % of WBC*	0.38 (+0.78/-0.25)	1.12 (+5.38/-0.93)	3.12 (+13.62/-2.54)	2.70 (+12.27/-2.21)	<0.0001/0.033
Sputum lymphocytes, % of WBC**	1.16 (1.05)	1.29 (1.42)	0.98 (1.19)	0.94 (1.25)	0.64/0.48
Sputum macrophages, % of WBC**	44.57 (19.42)	50.20 (31.17)	32.10 (21.45)	43.04 (26.46)	0.031/0.019
Sputum neutrophils, % of WBC**	53.57 (20.06)	43.88 (30.90)	56.99 (26.13)	48.10 (25.52)	0.19/0.13

*Geometric mean (asymmetric standard deviation) reported by cohort

**Mean (standard deviation) reported by cohort

***p-value (ANOVA F-test) for differences across severity cohorts, including/excluding healthy control cohort (based on log-transformed data when geometric means reported)

NA = not applicable (not measured in healthy cohort)

**Fig. 3:**
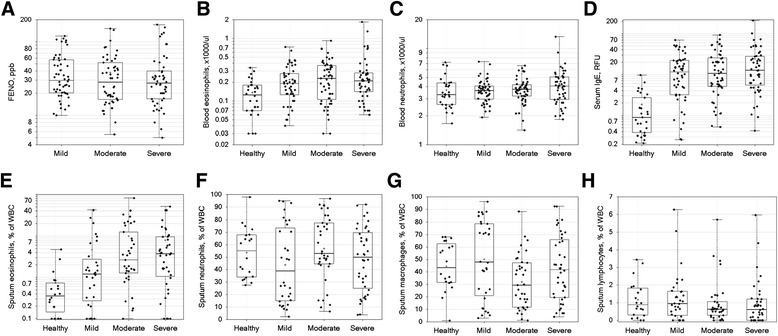
Clinical biomarkers and sputum leukocyte differentials. The values for the indicated clinical biomarkers (panels **a**-**d**) and sputum inflammatory cells (panels **e**-**h**) (y-axis) are shown for healthy control and asthma severity groups (x-axis). Data presented as symbols representing individual subjects and summarized by box (inter-quartile range and median) & whiskers (range), with ‘+’ indicating mean. Significance of differences among groups is reported in Table 4

Table 4 and Fig. 3 panels a-d by severity cohort. There was no significant impact of region on the clinical biomarkers.

#### FENO

At the baseline visit, geometric mean (asymptotic standard deviation) levels of FENO (ppb) were similar in mild (ICS-non-treated): 32.9 (+64.2/-16.9) ppb; moderate: 29.1 (+61.0/-13.9) ppb; and severe asthmatics: 28.8 (+64.7/-12.9) ppb (*p* = 0.59 for differences among asthma cohorts). Based on a high FENO cutoff of 35 ppb, 22/52 (42.3 %) mild, 24/54 (44.4 %) moderate, and 14/50 (28 %) severe asthmatics had a high FENO. Additionally, based on a low FENO cutoff of 20 ppb, 11/52 (21.1 %) mild, 24/54 (38.8 %) of moderate, and 14/50 (28 %) severe asthmatics had a low FENO.

#### Blood eosinophils

These were measured during screening only. Of the healthy controls, 28 of 31 (90 %) had bEOS < 300 cells/mm^3^ (a commonly used cutoff for “Th2” targeted therapeutics), compared to 12 of 52 (23 %), 21 of 55 (38 %), and 11 of 51 (22 %) for mild, moderate, and severe asthma. Geometric mean bEOS counts were significantly higher in asthma (*p* = 0.0018 for each asthma severity cohort vs. healthy controls) but not significantly different among asthma severity cohorts (*p* = 0.54).

#### Blood neutrophils

These were measured during screening only, without significant differences among the cohorts (*p* = 0.35), with geometric mean counts from 3.47, 3.59, 3.64, and 3.94 x1000/μl for healthy controls and mild, moderate, and severe asthma cohorts, respectively.

#### Serum IgE

This was measured at baseline in all healthy controls and asthma subjects, and was higher in asthmatics compared to healthy controls (*p* < 0.05 for each asthma severity cohort vs. healthy controls) but did not significantly differ among asthma severity cohorts (*p* = 0.35).

### Induced sputum inflammatory cells

Although sites had to meet a <20 % criteria for sputum squamous cells, samples with squamous cell content ≤30 % from cytospin differential counts were included in the analyses. A significant proportion of subjects had only a screening or only a baseline sample available that passed quality control standards (e.g., for cytospin slides, 105/189 possible subjects had acceptable readings at screening, 85 acceptable at baseline, with 128 subjects having either a screening and/or baseline read acceptable). Therefore, the mean (differential cell counts) or geometric mean (eosinophil percentage) of screening and baseline measurements was used for subsequent analyses of 128 of a possible 189 subjects.

The differential inflammatory white cell counts are reported in Table 4 and Fig. 3 panels e-h. Sputum eosinophil proportions were higher in each asthma severity cohort vs. healthy controls (*p* < 0.0001) and higher in moderate and severe asthma cohorts vs. the mild asthma cohort (*p* < 0.05 for each comparison). Sputum neutrophil percentages were lower in mild asthma vs. healthy controls and moderate asthma cohorts (*p* < 0.05 for each comparison). Sputum macrophage proportions were lower in moderate asthma vs. healthy controls and mild asthma cohorts (*p* < 0.05 for each comparison), with a similar trend for being lower compared to the severe asthma cohort (*p* = 0.099). Sputum lymphocytes did not significantly differ among cohorts.

### Correlation between clinical and biomarker variables

**Fig. 4:**
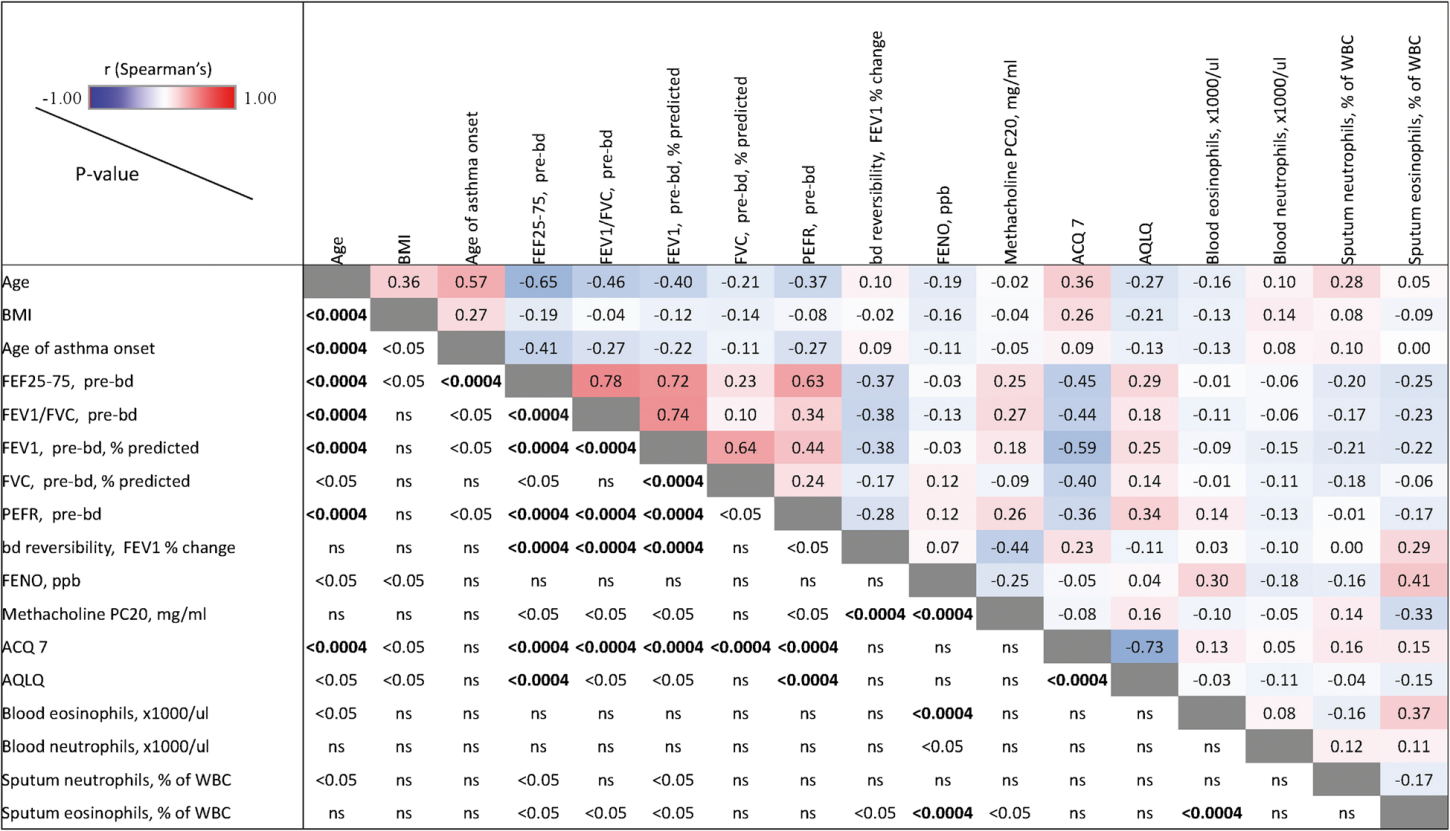
Correlation matrix across clinical and biomarker measures. Spearman correlation coefficients are reported for the pairing of variables indicated on the y-axis vs. x-axis, using values from asthma subjects only (healthy controls excluded) from baseline visit, for blood differentials from the screening visit, and for PC20 from either the screening or baseline visit. Color scale ranges from blue (*r* = −1) to white (*r* = 0) to red (*r* = 1). P- values <0.05 were considered to be statistically significant. Correlations among variables were tested using Spearman correlation tests that do not require assumptions of normality and linearity. Because of the large number of pair-wise comparisons, a significance threshold of *p* < 0.0004 was established by the Bonferroni adjustment method to maintain a family-wise error rate <0.05 for the 136 pair-wise comparisons of 16 variables

Correlations between demographic, asthma clinical, and biomarker variables are displayed in Fig. 4, reporting Spearman correlation coefficients. Spirometric measures were significantly inter-correlated, with preBD FEF25-75, FEV1 % predicted, and FEV1/FVC showing inter-correlations of *r* > 0.7 and preBD % predicted FEV1 and FVC correlating with *r* = 0.64 (*p* < 0.0001). Peak expiratory flow rate (PEFR) correlated best with the FEF25-75 (r = 0.63, *p* < 0.0001) but only modestly with FEV1 (*r* = 0.44, *p* < 0.0001). ACQ7 showed a significant negative correlation with AQLQ (*r* = −0.73, *p* < 0.0001) and FEV1 (*r* = −0.59, *p* < 0.0001). BDR showed a negative correlation with PC20 (*r* = −0.44, *p* < 0.0001). Blood eosinophils were only weakly correlated to FENO (*r* = 0.30, *p* = 0.0001) and spEOS (*r* = 0.37, *p* < 0.0001), with FENO demonstrating a trend for stronger correlation to spEOS (*r* = 0.41, *p* < 0.0001). Among the clinical variables evaluated, PC20 (*r* = −0.33, *p* = 0.0011) and BDR (*r* = −0.29, *p* = 0.0027) had the best, albeit very modest, correlations to spEOS. Blood neutrophil and macrophage counts were moderately correlated (*r* = 0.50, *p* < 0.0001), though neither were associated with asthma severity.

Despite the trends described above for the relationship of asthma disease characteristics with disease severity, the correlations between parameters were generally modest between different classes of clinical measurements.

## Discussion

The ADEPT study successfully profiled mild, moderate, and severe asthma compared to non-atopic controls with an acceptable safety profile for the invasive procedures. Although a moderately sized study, ADEPT accrued biomarker data across multiple matrices in the majority of subjects and evaluated several of these matrices repeatedly over 12 months.

The definitions of mild, moderate and severe asthma deviated somewhat from accepted definitions for the following reasons. Mild asthmatics were required to be off controller medications to allow evaluation of disease-related biomarkers without the impact of corticosteroids resulting in a very mild asthma cohort. The moderate and severe asthmatics were principally defined by ICS dose rather than lung function, primarily for practical reasons. For safety reasons in subjects undergoing invasive procedures, asthmatics were required to be free of recent exacerbations, to have no history of life-threatening asthma and BMI was limited to <32 kg/m^2^. Despite this limitation, obesity defined by a BMI >30 kg/m^2^ was increasingly prevalent in moderate and severe compared to mild asthma. Other studies have also identified a subset of asthmatics with obesity where distinct biological mechanisms may be driving disease severity [18–20].

The asthmatic bronchoscopy subset was restricted to age <55 years and this resulted in a significantly younger population by approximately 10 years. All participants in ADEPT had to be current nonsmokers (confirmed by urinary cotinine levels) to remove the confounding effect of tobacco smoke on biomarkers. We required healthy controls to be non-atopic by a specific IgE panel to provide a suitable contrast to asthmatics, who were in general atopic. The ADEPT severity definitions resulted in cohorts that differed in severity for multiple clinical parameters thus confirming their utility.

The methods of assessing allergic disposition were not in perfect agreement as might be expected from subjective (history by AHQ) and objective testing (sIgE, Phadiatop). The Phadiatop panel is composed of specific IgE against a panel of allergens for each region and is reported as a dichotomous variable. It is possible that low recent exposure could suppress titers or that relevant allergens were not captured for each subject.

Inclusion and exclusion criteria (see Additional file 1 linked to this report) were in part responsible for reducing the frequency of exacerbations. Although the mean number of severe exacerbations reported on the case report form (CRF) was low, 27 % of the severe asthmatics reported at least 1 OCS-treated exacerbation in the prior year. The AHQ presented additional data for “worsening of asthma requiring increase in medications” in that mild and moderate asthmatics had similar proportions of subjects with at least 1 event in contrast to severe asthma (60 %). This suggests that the inclusion criteria by asthma severity applied in ADEPT, and in particular the dose of ICS, succeeded to enrich for disease burden.

This was the first time we had used the AHQ in a study, and it should be regarded as an unvalidated instrument. Notwithstanding, the AHQ provided a more comprehensive insight into asthma history than that captured by the study coordinators in the CRF and highlighted some important differences between the asthma severity cohorts. Of note, the moderate and severe asthmatics had adult onset of disease more frequently than mild asthma, and reported more serious lung infections compared to mild asthma. Thus infection might be a driver for asthma severity. The discordance between allergy history by AHQ and CRF is discussed above. The increased prevalence of seasonal nasal allergy in mild asthma suggests that this might remit over time. Severe asthmatics reported nasal polyps more frequently, raising the possibility of aspirin sensitivity driving severity that has been reported to be associated with exacerbations [21]. The self-reported prevalence of obesity was highest in severe asthma but much less than BMI categorization, perhaps related to subject denial. The prevalence of nocturnal asthma was related to severity as has been previously recognized. Self-reported adherence to controller medications improved with worsening severity as might be expected.

Asthma disease characteristics present a more objective perspective on asthma severity and in general worsened with asthma severity in part dictated by inclusion criteria. Of note, the decrement in post-BD FVC in severe asthma is suggestive of air trapping perhaps related to more significant small airway disease [22] and/or loss of elastic recoil [23]. Inclusion/exclusion criteria were designed to exclude recently unstable but not necessarily poorly controlled asthma. Indeed, an objective was to enroll patients in the moderate and severe cohorts that were not adequately controlled by ICS based on persistent obstruction (FEV1 < 80 % predicted), corresponding to common therapeutic interventional clinical trials enrollment requirements. Accordingly, a significant proportion of the moderate (38 %) and severe asthma (61 %) cohorts were uncontrolled based on the ACQ-7 cut-off of 1.5.

Clinical biomarkers in ADEPT were selected for clinical practicality, with the exception of induced sputum which is not widely available. FENO levels were not different by asthma severity in agreement with one study [24] but in contrast to others [25, 26], perhaps related to less severe asthma in ADEPT and better response to ICS. Even though the mild asthmatics were not treated with inhaled corticosteroids, the absence of more elevated FENO may be due to a low inflammatory burden in this cohort.

The number of subjects who had valid sputum samples available for analysis was approximately 70 % for the screening and/or baseline visits despite considerable efforts to use experienced research sites and to train sites and subjects. The success rate in ADEPT which involved multiple centers, was less than that achievable in highly specialized single centers [27, 28] as might be expected. This illustrates that sputum induction as well as processing are technically complicated especially in a multicenter setting and probably not applicable to larger studies or clinical application, thus limiting utility.

Moderate and severe asthmatics had a significantly greater percentage of sputum eosinophils, and a trend for increase in % sputum neutrophils compared to mild and healthy controls despite treatment with ICS, but a significantly lower percentage of sputum macrophages. The persistence of sputum eosinophils in the moderate and severe asthmatics suggests a residual inflammatory burden that could be related to multiple factors e.g. ICS dose, ICS delivery to the airways, severity of disease, steroid resistance, and last but not least, patient adherence, which was not verified by objective means in ADEPT.

In general, the paucity of associations between multiple asthma characteristics may be due to distinct asthma domains e.g. lung function, inflammation, and patient history and experience. Of note, the weaker than expected correlation between bEOS and FENO and spEOS and blood perhaps related to the effect of ICS on FENO and spEOS, and less than on bEOS. Supporting this is the better correlation between FENO and spEOS as described previously [29, 30]. Eosinophilic inflammation assessed in sputum, but not in blood, showed weak correlations with worse AHR and a greater BDR which is in keeping with eosinophilic inflammation reflecting an at-risk phenotype [24].

European studies profiling efforts include ENFUMOSA (European Network for Understanding Mechanisms of Severe Asthma) [31] established in the mid-90s. ENFUMOSA, a multicenter cross sectional study, enrolled 163 patients with severe asthma and compared these to 158 mild asthma patients. A subset of subjects underwent induced sputum but bronchoscopy was not included. Important insights with regard to severe asthma from ENFUMOSA include persistent decrements in lung function and symptoms despite therapies, neutrophilic inflammation and identification of risk factors for severe asthma and near-fatal asthma. Subsequently, BIOAIR (Longitudinal Assessment of Clinical Course and BIOmarkers in Severe Chronic AIRway Disease [32]), an offshoot from ENFUMOSA, enrolled mild (*n* = 93), and severe asthma patients (*n* = 76) [33]. After optimization of inhaled therapy, patients were enrolled in a double blind 2 week trial of OCS versus placebo, followed by a one year follow up. The study revealed that spEOS and FENO were the best predictors of favorable response to oral prednisolone in severe asthmatics.

The Severe Asthma Research Program (SARP) is the biggest severe asthma program to date. In a review from 2012 [34], 1644 patients with asthma had been enrolled in a multicenter setting, of which 582 subjects had severe asthma. Subsets of these subjects underwent induced sputum evaluation [35] and 505 subjects including 151 severe asthma subjects underwent bronchoscopic sampling [34] with gene expression studies by way of example revealing novel phenotypes and pathways associated with FENO elevation [36]. Key findings from SARP have been reviewed periodically [34, 37], and clustering has been performed on subsets from this program [35, 38, 39] resulting in important insights into asthma phenotypes. Unlike ADEPT, SARP has included CT imaging in 424 subjects and MRI with hyperpolarized helium which has allowed evaluation of structure function relationships.

The more recent “Unbiased BIOmarkers in PREDiction of respiratory disease outcomes” (U-BIOPRED) consortium [40] has evaluated adult and pediatric asthmatic subjects with subsets also performing induced sputum and undergoing bronchoscopy. U-BIOPRED has a greater representation of refractory asthmatics treated with chronic OCS, and smoking asthmatics, than ADEPT, while pediatric patients were also enrolled. The study cohorts and biomarkers have been recently reported [41].

Limitations of the ADEPT population are predominantly due to protocol-mandated restrictions. Thus ADEPT did not include the most refractory asthmatic subjects (predominantly due to restrictions on lung function and recent exacerbations), currently smoking asthmatics (and those with > 10 pack year history), those with morbid obesity, and only 1 severe subject was treated with chronic OCS. Additionally, ADEPT did not include pediatric asthma and non-Caucasian representation was small. Finally, the enrollment of asthma subjects from different regions with varying geographies, ethnic composition and health-care delivery could have resulted in heterogeneity. However, there were few variables that were observed to be significantly impacted by regional differences.

## Conclusions

The ADEPT study evaluated multiple matrices including induced sputum and bronchoscopic sampling, and state of the art biomarker assessments. The ADEPT study enrolled the planned numbers of asthmatics across a range of severity, as well as non-atopic controls. Study procedures, including induced sputum and fibreoptic bronchoscopy, were performed safely. Clinical characteristics and biomarkers were differentiated in general by severity as intended. Future directions include overlaying gene expression profiles in airway samples and other matrices on the severity cohorts, and applying clustering methods to subset subjects and identify differential biology. The ADEPT data promises to be a rich repository that will help identify biological endotypes and help develop personalized therapies for asthma.

## Additional files

Additional file 1:
**ADEPT asthma study protocol.** (PDF 1042 kb) Additional file 2:
**Asthma History Questionnaire.** (DOCX 29 kb)

## Abbreviations

ACQ7: Asthma control questionnaire 7 (includes FEV1)
ADEPT: Airways Disease Endotyping for Personalized Therapeutics study
AE: Adverse event
AHQ: Asthma history questionnaire
AHR: Airways hyperresponsiveness
ANOVA: Analysis of variance
AQLQ: Asthma Quality of Life Questionnaire
ATS: American Thoracic Society
BDR: Bronchodilator reversibility
bEOS: Blood eosinophils
BIOAIR: Longitudinal Assessment of Clinical Course and BIOmarkers in Severe Chronic AIRway Disease study
CCL: C-C motif chemokine ligand
CLCA1: Calcium-activated chloride channel regulator 1
CRF: Case report form
DNA: Deoxyribonucleic acid
ENFUMOSA: European Network for Understanding Mechanisms of Severe Asthma
ERS: European Respiratory Society
FENO: Fractional concentration of exhaled nitric oxide
EBBX: Endobronchial biopsy
ELISA: Enzyme-linked immunosorbent assay
ENFUMOSA: European Network for Understanding Mechanisms of Severe Asthma
EPBR: Epithelial brushing
EOS: Eosinophils
FEV1: Forced expired volume in 1 s
FVC: Forced vital capacity
FEF25-75: Forced expired flow averaged between 25 and 75 % of the vital capacity
ICS: Inhaled corticosteroids
IgE: Immunoglobulin E
IL: Interleukin
IL-4R: Interleukin 4 receptor
IS: Induced sputum
LABA: Long-acting β-2 agonist
MAb: Monoclonal antibody
ml: milliliters
NHANES: National Health and Nutrition Examination Survey
NHLBI: National Heart Lung and Blood Institute
NA: Not applicable
OCS: Oral steroids
PEFR: Peak expiratory flow rate
PC20: The provocative concentration of methacholine resulting in a 20 % or greater fall in the forced expired volume in 1 s
POSTN: Periostin
ppb: Parts per billion
Pre-BD: Prebronchodilator
Post-BD: Post-bronchodilator
RFU: Relative Fluorescence Units
RNA: Ribonucleic acid
SABA: Short-acting β-2 agonist
SAE: Serious adverse events
SARP: Severe Asthma Research Program
SerpinB2: Serpin peptidase inhibitor, clade B, member 2 (also known as plasminogen activator inhibitor-2)
sIgE: Serum immunoglobulin E
spEOS: Sputum eosinophils
Th2: T helper cell type 2
U-BIOPRED: Unbiased Biomarkers in the Prediction of Respiratory Disease Outcomes consortium
WBC: White blood cells.
