# Supplementary material for: Asthma characteristics and biomarkers from the Airways Disease Endotyping for Personalized Therapeutics (ADEPT) longitudinal profiling study
Source: Respir Res. 2015 Nov 17;16:142. doi: 10.1186/s12931-015-0299-y (PMC4650115; doi:10.1186/s12931-015-0299-y)
Supplement: Additional file 1: — ADEPT asthma study protocol. (PDF 1042 kb) [file 12931_2015_299_MOESM1_ESM.pdf]

Janssen Research & Development

Protocol NOCOMPOUNDASH0001
AMENDMENT 3

A Multicenter Longitudinal Study for Disease Profiling of Asthma

---

*Janssen Research & Development is a global organization that operates through different legal entities in various countries. Therefore, the legal entity acting as the sponsor for Janssen Research & Development studies may vary, such as, but not limited to Janssen Biotech, Inc.; Janssen Products, LP; Janssen Biologics, BV; Janssen-Cilag International NV; Janssen, Inc; Janssen Infectious Diseases BVBA; Janssen R&D Ireland; or Janssen Research & Development, LLC. The term "sponsor" is used throughout the protocol to represent these various legal entities; the sponsor is identified on the Contact Information page that accompanies the protocol.

Issue/Report Date: Incorporates Amendment 3: 29 May 2012
Amendment 2: 22 Apr 2011
Amendment 1: 17 Nov 2010
Original Protocol: 12 Apr 2010

Compliance: This study will be conducted in compliance with this protocol, Good Clinical Practice, and applicable regulatory requirements.

Confidentiality Statement

The information in this document contains trade secrets and commercial information that are privileged or confidential and may not be disclosed unless such disclosure is required by applicable law or regulations. In any event, persons to whom the information is disclosed must be informed that the information is *privileged* or *confidential* and may not be further disclosed by them. These restrictions on disclosure will apply equally to *all* future information supplied to you that is indicated as *privileged* or *confidential*.

TABLE OF CONTENTS

TABLE OF CONTENTS 2

SYNOPSIS 5

ABBREVIATIONS 7

1 INTRODUCTION 9
- 1.1 Background 9
- 1.2 Overall Rationale for the Study 10

2 OBJECTIVES 10

3 OVERVIEW OF STUDY DESIGN 11
- 3.1 Study Design Rationale 12

4 STUDY POPULATION 13
- 4.1 General Considerations 13
- 4.2 Healthy Subjects 14
  - 4.2.1 Inclusion Criteria 14
  - 4.2.2 Exclusion Criteria 15
- 4.3 Subjects with Asthma 16
  - 4.3.1 Inclusion Criteria for Asthmatic Subjects 16
  - 4.3.2 Exclusion Criteria for Asthmatic Subjects 18

5 STUDY SUBJECT ALLOCATION 19

6 PRESTUDY AND CONCOMITANT THERAPY 19

7 STUDY EVALUATIONS 20
- 7.1 Screening Procedures 21
- 7.2 Study Procedures 22
  - 7.2.1 Baseline Visit 22
  - 7.2.2 Bronchoscopy Visit 23
  - 7.2.3 Biomarker Visits (Asthmatic Subjects) 24
- 7.3 Biomarker Evaluations 25
  - 7.3.1 Serum and Urine Biomarkers 25
  - 7.3.2 Whole Blood Gene Expression Profiling 25
  - 7.3.3 Bronchoscopy (Endobronchial Biopsies and Brushings) 25
  - 7.3.4 Induced Sputum 25
- 7.4 Spirometry and Bronchodilator Response 26
- 7.5 Methacholine Challenge 27
- 7.6 Fractional Exhaled Nitric Oxide (FENO) 27
- 7.7 Asthma Event Log 27
- 7.8 Asthma Quality of Life Questionnaire (AQLQ) 28
- 7.9 Asthma Control Questionnaire (ACQ) 28
- 7.10 Asthma History Questionnaire 28
- 7.11 Safety Evaluations 29
- 7.12 Genomic Evaluation 30

8 SUBJECT COMPLETION/WITHDRAWAL 31
- 8.1 Completion 31
- 8.2 Withdrawal From the Study 31

9 STATISTICAL METHODS 32
- 9.1 Subject Information 32

9.2 Sample Size 32
9.3 Interim Analyses 33
9.4 Biomarker Data Analyses 34
9.5 Safety Analyses 34

10 ADVERSE EVENT REPORTING 35
10.1 Definitions 35
10.1.1 Adverse Event Definitions and Classifications 35
10.1.2 Attribution Definitions 36
10.1.3 Severity Criteria 37
10.2 Procedures 37
10.2.1 All Adverse Events 37
10.2.2 Serious Adverse Events 38
10.2.3 Pregnancy 39
10.3 Contacting Sponsor Regarding Safety 39

11 ETHICAL ASPECTS 39
11.1 Study-Specific Design Considerations 39
11.2 Regulatory Ethics Compliance 40
11.2.1 Investigator Responsibilities 40
11.2.2 Independent Ethics Committee or Institutional Review Board (IEC/IRB) 40
11.2.3 Informed Consent 42
11.2.4 Privacy of Personal Data 43
11.2.5 Long-Term Storage of Samples for Future Research 44

12 ADMINISTRATIVE REQUIREMENTS 44
12.1 Protocol Amendments 44
12.2 Regulatory Documentation 44
12.2.1 Regulatory Approval/Notification 44
12.2.2 Required Prestudy Documentation 45
12.3 Subject Identification, Enrollment, and Screening Logs 46
12.4 Source Documentation 46
12.5 Case Report Form Completion 46
12.6 Data Quality Assurance/Quality Control 47
12.7 Record Retention 47
12.8 Monitoring 48
12.9 Study Completion/Termination 48
12.9.1 Study Completion 48
12.9.2 Study Termination 49
12.10 On-Site Audits 49
12.11 Use of Information and Publication 49

13 REFERENCES 52

ATTACHMENT 1: SCHEDULE OF EVENTS 53
Attachment 1.1 Study Assessments for Healthy Subjects 53
Attachment 1.2 Study Assessments for Asthmatic Subjects Receiving a Bronchoscopy 55
Attachment 1.3 Study Assessments for Asthmatic Subjects Not Receiving a Bronchoscopy 58

ATTACHMENT 2: CENTRAL LABORATORY ASSESSMENTS 61

ATTACHMENT 3 PROTOCOL HISTORY 62

AMENDMENT 1 - 17 NOV 2010 63

AMENDMENT 2 - 22 APR 2011 87

AMENDMENT 3 - 29 MAY 2012 96

APPENDIX A: NHLBI ASTHMA CLASSIFICATION SYSTEM 99

APPENDIX B: NHLBI CLASSIFICATION OF DAILY DOSE LEVELS FOR INHALED CORTICOSTEROIDS FOR YOUTHS ≥ 12 YEARS OF AGE AND ADULTS 102

SYNOPSIS

A Multicenter Longitudinal Study for Disease Profiling of Asthma

OBJECTIVES

Primary Objectives

The objective of this study is to characterize the clinical, physiologic, and molecular profiles of healthy subjects and subjects with mild, moderate, and severe asthma.

Hypothesis

There is no formal hypothesis to be tested in this study.

However, this exploratory study is based on the hypothesis that specific subpopulations of patients with asthma exist with distinct molecular and clinical phenotypes of disease that will be identified through the profiling methodology employed in this study. The data obtained in this study will aid in the evaluation of new and existing therapeutics for asthma and may allow for targeted selection of subpopulations of asthmatic subjects for clinical trials. In addition it may facilitate asthma drug discovery by furthering the understanding of non-clinical models of disease and their relation to human disease.

OVERVIEW OF STUDY DESIGN

This is a multi-center, longitudinal, exploratory study of biomarkers, clinical and physiological parameters in subjects with mild, moderate, severe asthma and healthy control subjects. There is no therapeutic intervention and this protocol will not restrict or introduce any medical interventions including medications. Study participants will undergo procedures that include pulmonary function testing, assessment of airway reactivity, collection of blood samples for routine laboratory tests, biomarkers and DNA evaluation (in further consenting subjects), induced sputum collection, and exhaled nitric oxide collection. All subjects with asthma will have additional follow-up visits at 3, 6, and 12 months for repeat sample collection for biomarker analysis and the assessment of clinical and physiological parameters. Up to thirty subjects from each asthma severity group and all healthy subjects will undergo a bronchoscopy for collection of endobronchial biopsy and brushing samples. These samples will be used for analysis of proteins, RNA and other biomarkers in endobronchial tissue and epithelial cells.

STUDY POPULATION

Approximately 150 subjects with asthma (50 subjects in each asthmatic class of mild, moderate and severe) and 30 healthy subjects will be enrolled in this study for disease profiling. Healthy subjects will be divided approximately equally into 2 age ranges of 18 to 36 years or 37 to 55 years.

BIOMARKER EVALUATIONS

Biomarker assessments will include the evaluation of biomarkers in serum, urine, and whole blood as well as from endobronchial biopsy, brushings and induced sputum specimens. Potential markers include but are not limited to TNFα, IL-8, eotaxin, myeloperoxidase, MMP2, MMP9, IL-4, IL-13, cysteinyl leukotrienes, 8-isoprostane, mRNA and miRNA differential expression profiles.

GENOMIC EVALUATIONS

A blood sample for genomic analysis will be collected for research purposes from subjects that provide consent and where local regulations permit.

SAFETY EVALUATIONS

All subjects will be monitored for adverse events related to study procedures.

STATISTICAL METHODS

No formal hypothesis testing will be conducted. Data will be summarized using descriptive statistics. Continuous variables will be summarized using the number of observations, mean, standard deviation (SD), minimum, median, and maximum values as appropriate. Categorical values will be summarized using the number of observations and percentages as appropriate.

Sample sizes of 50 subjects per asthma severity group and 30 healthy subjects were chosen based on previous experience with microarray datasets. The power and effect size to detect differential expression between asthma severity groups and the control group and among the asthma severity groups was evaluated. The proposed sample size will appropriately generate descriptive measures on exploratory biomarkers.

ABBREVIATIONS

ACQ	asthma control questionnaire
AE	adverse event
AQLQ	asthma quality of life questionnaire
BMI	body mass index
CBC	complete blood count
CDM	clinical data manager
CLCA1	calcium-activated chloride channel regulator 1
COPD	chronic obstructive pulmonary disease
CRF	case report form
DCF	data clarification form
DNA	deoxyribonucleic acid
ECG	electrocardiogram
eDC	electronic data capture
ER	emergency room
FDA	Food and Drug Administration
FENO	fractional exhaled nitric oxide
FEV_1	forced expiratory volume in the first second
FVC	forced vital capacity
GCP	Good Clinical Practices
GI	gastrointestinal
HBsAg	hepatitis B surface antigen
HCV	hepatitis C virus
HIV	human immunodeficiency virus
ICH	International Conference on Harmonisation
ICS	inhaled corticosteroid
ICU	intensive care unit
IEC	Independent Ethics Committee
IgE	immunoglobulin E
IL	interleukin
IRB	Institutional Review Board
LABA	long acting β-2 agonist
LTRA	leukotriene receptor antagonist
MedDRA	Medical Dictionary for Regulatory Activities
MiRNA	mitochondrial ribonucleic acid
MMP	matrix metalloprotein
mRNA	messenger ribonucleic acid
NHLBI	National Heart, Lung, and Blood Institute
NIH	National Institutes of Health
NSAID	nonsteroidal anti-inflammatory drug
NO	nitric oxide
OCS	oral corticosteroid
PC20	provocation concentration of methacholine needed to produce a 20% fall in FEV_1 from baseline
PEFR	peak expiratory flow rate
PFT	pulmonary function test
PI	principal investigator
PRN	per rising need or as needed
RNA	ribonucleic acid

SABA	short acting β-2 agonist
SD	standard deviation
SM	site manager
SOP	standard operating procedure
TB	tuberculosis
Th2	T helper lymphocyte 2
TNFα	tumor necrosis factor alpha

1 INTRODUCTION

Asthma is a heterogeneous inflammatory syndrome of reversible airflow obstruction and airway hyperreactivity leading to symptoms such as dyspnea, cough, wheezing and asthma exacerbations which may lead to hospitalization and even mortality. Over long periods, remodeling of airways and lung parenchyma occurs leading to fixed airflow obstruction which may worsen over time. While there are many ways to classify the phenotypes of asthma, the underlying biology of these phenotypes is poorly understood, and a deeper understanding of molecular and cellular events in different severity asthma cohorts may aid in identification of subjects more responsive to existing therapeutics and in the identification of new therapeutic targets.

1.1 Background

Airway inflammation, intermittent airflow obstruction, and bronchial hyperresponsiveness represent major components of asthma pathophysiology (Barnes, 2008; Linzer, 2007; Maddox and Schwartz, 2002). It has been proposed that an underlying cause of these components is inflammation that can be acute, subacute, or chronic (NIH, 1997; Toews, 2001). In addition, airway edema (Chu et al, 2001; Rogers and Evans, 1992) and mucus secretion (Rogers, 2004) also contribute to airflow obstruction and bronchial reactivity. Chronic airway inflammation leads to airway remodeling as characterized by subendothelial fibrosis, goblet cell hyperplasia, smooth muscle hypertrophy, thickening of basement membrane, and inflammatory cell infiltration (Maddox and Schwartz, 2002; Tiddens et al, 2000). Also, increased epithelial shedding into the bronchial lumen (Yoshihara et al, 2006) could lead to exposure of sensory nerve endings and an imbalance in cholinergic and peptidergic neuronal control contributing to persistent airway obstruction.

The molecular and cellular mechanisms leading to airway inflammation as a response to different environmental triggers are not yet well understood although significant progress has been made. Some of the principal cells identified in airway inflammation include mast cells, neutrophils, eosinophils, epithelial cells, macrophages, and activated T lymphocytes. Numerous cytokines released by T lymphocytes play an important role in the regulation of airway inflammation. In addition, other airway cells such as fibroblasts, endothelial cells, and epithelial cells also contribute to the chronicity and severity of the disease (Yoshihara et al, 2006). On the molecular level, pro-inflammatory cytokines and other factors such as adhesion molecules (eg, selectins, integrins), lipid mediators (prostaglandins and leukotrienes) (Huang and Peters-Golden, 2008), oxygen radicals, and toxic granule proteins are critical in directing the inflammatory changes in the airway. Finally, cell-derived mediators influence smooth muscle tone and produce structural changes and remodeling of the airway (Barnes, 2008; Tiddens et al, 2000).

Lack of well defined markers for different disease phenotypes has been one of the main obstacles in understanding the natural progression of asthma and implementing the most

adequate treatment. Recent studies (Woodruff et al, 2007; Woodruff et al, 2009) identified subsets of asthma patients with respect to the molecular mechanisms underlying airway inflammation. In these studies, messenger ribonucleic acid (mRNA) microarray expression profiles were obtained from airway epithelial cells (obtained by bronchoscopy/epithelial brushings) of 42 mild to moderate steroid-naïve asthma subjects and 28 healthy non-smoking control subjects. It was found that these asthma subjects had an increased expression of CLCA1, periostin, ovalbumin and serpinB2, compared to healthy controls (Woodruff et al, 2007), and that the mRNA expression levels of these genes could predict responsiveness to inhaled corticosteroids (Woodruff et al, 2007). The identified genes are highly regulated by IL-13 in cultured airway epithelial cells (Woodruff et al, 2009). In addition, the data suggested that asthma subjects could be divided into at least 2 distinct phenotypes (Th2-low and Th2-high) depending on the level of T helper lymphocyte 2 (Th2) inflammation as assessed by the expression level of IL-13 (Woodruff et al, 2009). The Th2-low asthma subgroup did not reveal any additional clustering suggesting that in at least 50% of asthma subjects other processes not related to Th2 may contribute to asthma phenotypes.

This study is designed to identify molecular and cellular profiles in peripheral blood, urine, induced sputum, and bronchial tissue from three main categories of asthma (persistent mild, moderate and severe) and to correlate these profiles to respective clinical phenotypes.

1.2 Overall Rationale for the Study

This study will focus on evaluating the biology of asthma of varying severities including a comparison to healthy nonatopic controls in order to aid in the evaluation of new and existing therapeutics for asthma and also to better link non-clinical disease models to human asthma. This may allow for targeted selection of subpopulations of asthmatic subjects for clinical trials and help understand the heterogeneity of response.

2 OBJECTIVES

Primary Objective

The objective of this study is to characterize the clinical, physiologic, and molecular profiles of healthy subjects and subjects with mild, moderate, and severe asthma.

Hypothesis

There is no formal hypothesis to be tested in this study.

However, this exploratory study is based on the hypothesis that specific subpopulations of asthma exist with distinct molecular and clinical phenotypes of disease that can be identified through the profiling methodology employed in this study. The data obtained in this study will aid in the evaluation of new and existing therapeutics for asthma and may allow for targeted selection of subpopulations of asthmatic subjects for clinical

trials. In addition, it may facilitate asthma drug discovery by furthering the understanding of non-clinical models of disease and their relation to human disease.

3 OVERVIEW OF STUDY DESIGN

Approximately 180 subjects will be enrolled with up to 50 subjects in each of the mild, moderate and severe asthma cohorts and 30 subjects in the healthy subject cohort. There is no study related therapeutic intervention and this protocol will not restrict or introduce any therapeutic interventions including medications. All subjects will continue to be managed by their personal physicians.

All eligible subjects will be enrolled during screening visits and then undergo the study procedures which will include medical history and physical exam, patient reported outcomes and questionnaires for asthmatic subjects only, pulmonary function testing (PFT), assessment of airway reactivity, blood for routine laboratory tests, biomarkers and DNA evaluation (in consenting subjects), induced sputum collection and exhaled nitric oxide collection. A bronchoscopy will be performed on up to 30 healthy subjects and on up to 30 asthma subjects from each of the asthma severity cohorts. The healthy subject cohort will be evenly split into two age ranges of 18 to 36 years of age or 37 to 55 years of age. All subjects with asthma will have 3 subsequent visits at 3, 6 and 12 months after the baseline visit for repeat sample collection (PFT, exhaled nitric oxide (NO), blood, urine and sputum only) to evaluate stability of phenotypes. The overall study schema is provided in Figure 1.

Two database locks are planned: an interim database lock after completion of the last bronchoscopy procedures for the last subject receiving a bronchoscopy; a second database lock will occur at the end of the study after the last scheduled visit has occurred for the last subject in the study.

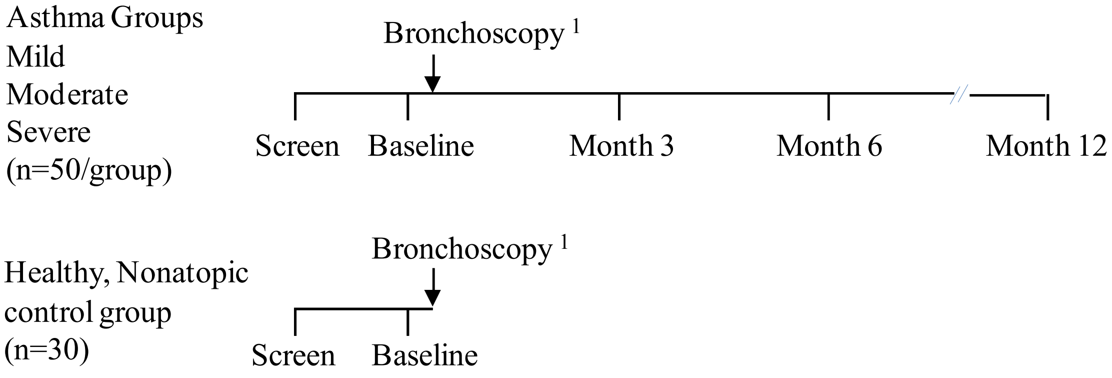

[1] 30 subjects from each asthma severity group and 30 healthy subjects, within 5-14 days after baseline

Figure 1 Study Schema

3.1 Study Design Rationale

Although clinical manifestations of asthma are well characterized the underlying molecular and cellular events that play a role in its development and progression are not yet well understood. This lack of understanding hampers the development of more effective asthma therapies. This study is designed to better understand the cell types and molecular mediators that characterize specific asthma severity phenotypes (mild, moderate and severe asthma). Biomarker analysis of blood and induced sputum at the cellular, protein, and mRNA levels will enable better understanding of asthma phenotypes. In addition histological, cellular and mRNA analysis of endobronchial biopsy and epithelial brushing samples will allow for bringing a bedside understanding of the disease to bench-side understanding of research animal models. This information will facilitate the identification of new therapeutic targets for asthma, the discovery of novel therapeutics for those targets and patient stratification for existing therapeutics.

Asthma has a heterogeneous disease etiology and is clinically presented in a broad range of severity. The diagnosis of asthma is based on characteristic symptoms, physician confirmation of the diagnosis, and confirmation of bronchodilator FEV_1 reversibility. If confirmation of bronchodilator FEV_1 reversibility is lacking, then evaluation of airway hyperreactivity to methacholine may be used to confirm the diagnosis. If hyperreactivity to methacholine cannot be performed at screening due to a low FEV_1, or the subject does not have historical data to document hyperreactivity, then, an obstructive airway deficit as demonstrated by a FEV_1/FVC ratio of ≤ 0.7 may be used to support the diagnosis.

The inclusion and exclusion criteria detailed in Section 4 of this protocol should enroll true asthmatic subjects and reduce the chances of enrolling subjects with other pulmonary conditions into this study. The inclusion of asthmatic subjects in the mild, moderate, and severe categories will provide important information about the pathobiology that accompanies these clinical categories. The healthy subjects will be screened for the absence of atopy, asthma, or other pulmonary conditions. Serial measurements over 12 months in the asthmatic subjects will allow reproducibility and stability of the initial phenotypes to be assessed.

DNA Collection

It is recognized that genetic variation can be an important contributory factor to inter-individual differences in drug distribution and response and can also serve as a marker for disease susceptibility and prognosis. Genomic research may help to explain inter-individual variability in clinical outcomes and may help to identify population subgroups that respond differently to a drug. The goal of the genomic component is to collect DNA to potentially identify genetic factors that may influence the asthma phenotype in a particular individual. A separate consent will be signed by all subjects who agree to DNA sampling.

4 STUDY POPULATION

4.1 General Considerations

Approximately 150 asthmatic subjects and 30 healthy subjects will be enrolled in this study. Those subjects who are scheduled for a bronchoscopy and withdraw from the study prior to the bronchoscopy will be replaced. Approximately equal numbers of healthy subjects are planned to be enrolled into 2 age groups (18 to 36 years of age or 37 to 55 years of age).

The National Heart, Lung, and Blood Institute (NHLBI) expert panel (NIH, 1997) provided a guideline for asthma subjects' classification into respective asthma severity groups (Table A.1, Appendix A). Medication dose levels used for the treatment of asthma are also defined by the NHLBI (Appendix B) (NIH, 2007). Approximately 50 subjects will be recruited in each of the 3 asthma categories (mild, moderate, severe) as defined below. Note that not all asthmatic subjects will qualify for this study (see Table A.2, Appendix A). The categories are defined as:

- Mild:
  - Prebronchodilator $FEV_1 \geq 80\%$ of predicted normal values measured > 6 hours after last use of bronchodilator **AND**
  - No asthma controller medication in the 6 weeks prior to screening, but may be taking short acting β-2 agonist (SABA) on an as needed (PRN) basis (NHLBI STEP 1; Appendix A).

- Moderate Persistent:
  - Prebronchodilator FEV_1 from ≥ 60% to < 80% of predicted normal values measured > 6 hours after last use of bronchodilator **AND**
  - Treatment with SABA PRN **AND**
  - Low to medium dose inhaled corticosteroid (ICS) alone, or low to medium dose ICS in combination with any other controller medication (eg LABA, leukotriene antagonist (LTRA), theophylline, etc,) with the exception of oral corticosteroids (OCS) and omalizumab (NHLBI STEP 2, 3, or 4; Appendix A).
- Severe Persistent:
  - Prebronchodilator FEV_1 from ≥ 50% to < 80% of predicted normal values measured > 6 hours after last use of bronchodilator **AND**
  - Treatment with SABA PRN **AND**
  - High dose ICS alone or in combination with any other controller medication (eg, LABA, LTRA, theophylline, anticholinergics, OCS, anti-IgE, etc) (NHLBI STEP 5 or 6; Appendix A).

The inclusion and exclusion criteria for enrolling subjects in this study are described in the following sections. If there is a question about the inclusion or exclusion criteria below, the investigator should consult with the appropriate sponsor representative before enrolling the subject in the study.

4.2 Healthy Subjects

4.2.1 Inclusion Criteria

Subjects must satisfy all of the following criteria to be enrolled in the study:

1. Must have signed an informed consent indicating that they understand the purpose of and procedures required for the study and are willing to participate in the study.
2. Be willing and able to adhere to the study visit schedule and other protocol requirements.
3. Be between 18 and 55 years of age, inclusive, at informed consent.
4. Have no clinically significant abnormalities as determined by medical history, physical examination, blood chemistry assessments, hematologic assessments including complete blood count (CBC), urinalysis (see Attachment 2 for clinical laboratory analyses), measurement of vital signs, and ECG.
5. Have a body mass index (BMI) of ≤ 32 kg/m^2.
6. Must be able to produce an adequate induced sputum sample at screening, defined as a selected plug weight of at least 50 mg and a squamous cell count of < 20% (see Study Reference Manual).

7. Must be judged by the Principal Investigator (PI) to be a subject suitable to undergo bronchoscopy.
8. Have no history of chronic respiratory disease including asthma.
9. Have no history of allergic symptoms eg, allergic rhinitis, eczema.
10. Be a non-smoker for ≥ 1 year at initial screening visit and have ≤ 10 pack-year history of smoking.
11. No other acute illness in the 6 weeks prior to screening.
12. No contraindications to the procedures in this study including clinical or research bronchoscopy.
13. No bleeding disorder including use of anticoagulants and antiplatelet agents that could place the subjects at risk for bleeding. Able to abstain from aspirin and use for 7 days and NSAID use for 3 days prior to bronchoscopy without risk.
14. No contraindications to conscious sedation or medications used in the bronchoscopy procedure.
15. To participate in the optional genomic component of this study, subjects must have signed the informed consent for genomic research indicating willingness to participate in the genomic component of the study. Refusal to give consent for this component does not exclude a subject from participation in the study.

4.2.2 Exclusion Criteria

Subjects who meet any of the following criteria will be excluded from participating in the study:

1. Be considered, in the opinion of the investigator, to be an unsuitable candidate for the study.
2. History of any clinically significant medical illness or medical disorders including (but not limited to) cardiovascular disease, neuromuscular, hematological disease including bleeding disorders, respiratory disease, hepatic or gastrointestinal (GI) disease, neurological or psychiatric disease, ophthalmological disorders, neoplastic disease, renal or urinary tract diseases, or dermatological disease.
3. Diagnosis of chronic obstructive pulmonary disease (COPD), cystic fibrosis, or other significant respiratory disorder including significant occupational or environmental exposures with ongoing respiratory symptoms.
4. Have a bronchodilator response of ≥ 12% and at least 200 mL from baseline or an FEV_1 value < 85% of predicted value at screening.
5. A known history of sleep apnea requiring medical intervention.
6. Major or traumatic surgery within the 3 months prior to screening.
7. Have a positive urine pregnancy screening result.
8. Have a recent history (within previous 6 months) of alcohol or drug abuse.

9. Positive urine toxicology screen for substances of abuse, including but not limited to amphetamines, barbiturates, benzodiazepines, cannabinoids, cocaine, methadone, opiates and tricyclic antidepressants.
10. Have a positive urine screen for nicotine (urine cotinine test).
11. Have a positive breath test for alcohol at initial screening visit.
12. Have a positive serology test for HIV antibodies, hepatitis B surface antigen (HBsAg), or hepatitis C virus antibody (anti-HCV) at screening.
13. Have a positive Phadiatop test.
14. Donated blood (volume ≳ 500 mL) within 56 days prior to screening.
15. Received an experimental antibody or biologic therapy within the 6 months prior to screening, or received any other experimental therapy or new investigational agent within 60 days or 5 half-lives (whichever is longer) of study start.
16. Be unable or unwilling to undergo multiple venipunctures because of poor tolerability or lack of easy access to veins.
17. Is an employee or employee family member of the investigator, study center, or Sponsor.
18. Any condition that, in the opinion of the investigator, would complicate or compromise the study, or the well-being of the subject.

4.3 Subjects with Asthma

4.3.1 Inclusion Criteria for Asthmatic Subjects

Subjects must satisfy all of the following criteria to be enrolled in the study:

1. Must have signed an informed consent indicating that they understand the purpose of and procedures required for the study and are willing to participate in the study.
2. Be willing and able to adhere to the study visit schedule and other protocol requirements.
3. Be between 18 and 70 years of age, inclusive, at informed consent. Subjects who elect to undergo bronchoscopy must be between 18 and 55 years old, inclusive, at informed consent.
4. Have no clinically significant abnormalities as determined by medical history, physical examination, blood chemistry assessments, hematologic assessments including CBC, urinalysis (see Attachment 2 for clinical laboratory analyses), measurement of vital signs, and ECG.
5. Have a BMI ≤ 32 kg/m^2.
6. Must be able to produce an adequate induced sputum sample at screening, defined as a selected plug weight of at least 50 mg and a squamous cell count of < 20% (see Study Reference Manual).

7. Be a non-smoker for ≥ 1 year prior to screening and have ≤ 10 pack- year history of smoking.
8. No contraindications to the procedures in this study.
9. Symptoms compatible with asthma for at least 6 months prior to screening (wheezing, dyspnea, chest tightness), PI confirmation of diagnosis of asthma of any severity and exclusion of alternative diagnoses, and at least 1 one the following, tested sequentially at screening or based on historical documentation:
   - Bronchodilator reversibility after inhalation of SABA at screening or documented within the previous 24 months defined as an increase in FEV_1 ≥ 12% and at least 200 mL from baseline. If reversibility is < 12% and per the opinion of the PI, a repeat bronchodilator reversibility test is performed; this result may be used for subject enrollment.
   - PC20 methacholine ≤ 16 mg/mL at screening or documented within 24 months prior to screening. If the PC20 is > 16 mg/mL, the subject is ineligible.
   - Obstructive physiology defined as a FEV_1/FVC ratio < 0.7.
10. Fall into 1 of the categories for asthma severity (mild, moderate, or severe) as described in the study population (see Section 4.1).
11. Prebronchodilator FEV_1 ≥ 50% predicted at screening.
12. Must have clinically stable asthma (see Section 7.2.2) for at least 6 weeks prior to screening.
13. No acute illness including asthma exacerbation requiring augmentation of therapy in the 6 weeks prior to screening.
14. Have been on their current asthma controller therapy for at least 6 weeks prior to screening. Mild asthmatics must have been off asthma controller therapy for at least 6 weeks prior to screening. **Addition or withdrawal of asthma controller medications in order to be considered for participation in the study is prohibited**.
15. For asthmatic subjects participating in bronchoscopy:
   - Must be between 18 and 55 years of age, inclusive, at informed consent.
   - Must be judged by the PI to be a subject suitable to undergo bronchoscopy.
   - No lifetime medical history of life-threatening asthma including intubation and ICU admission.
   - Post-bronchodilator FEV_1 ≥ 60% predicted at screening.
   - No history of adverse events associated with a prior bronchoscopy, including but not limited to, significant worsening of asthma, significant bleeding or reaction to sedative agents.
   - No history of bleeding disorder including use of anticoagulants and antiplatelet agents that could place the subjects at risk of bleeding. Able to abstain from aspirin use for 7 days prior to bronchoscopy without risk.

   – No contraindications to conscious sedation or medications used in the bronchoscopy procedure.

16. To participate in the optional genomic component of this study, subjects must have signed the informed consent for genomic research indicating willingness to participate in the genomic component of the study. Refusal to give consent for this component does not exclude a subject from participation in the study.

4.3.2 Exclusion Criteria for Asthmatic Subjects

1. Be considered, in the opinion of the investigator, to be an unsuitable candidate for the study.
2. History of any clinically significant medical illness or medical disorders that place the subject at risk from participation by the judgment of the PI including (but not limited to) cardiovascular disease, neuromuscular, hematological disease including bleeding disorders, respiratory disease, hepatic or GI disease, neurological or psychiatric disease, ophthalmological disorders, neoplastic disease, renal or urinary tract diseases, or dermatological disease. Subjects with stable, well-controlled conditions may be eligible after consultation with the Sponsor's medical monitor.
3. Diagnosis of allergic bronchopulmonary aspergillosis (ABPA), allergic bronchopulmonary mycosis (ABPM), or occupational asthma.
4. Diagnosis of COPD, cystic fibrosis, or other significant respiratory disorder including significant occupational or environmental exposures with ongoing respiratory symptoms.
5. If a PC20 methacholine test is performed during screening for enrollment, a PC20 result > 16 mg/mL excludes the subject from the study (refer to 4.3.1, inclusion criterion #9).
6. Major or traumatic surgery within 12 weeks of screening.
7. A known history of sleep apnea requiring medical intervention.
8. Have a positive urine pregnancy screening result.
9. Have a recent history (within previous 6 months) of alcohol or drug abuse.
10. Positive urine toxicology screen for substances of abuse, including but not limited to, cannabinoids, cocaine, and methadone.
11. Positive urine toxicology screen, including but not limited to amphetamines, barbiturates, benzodiazepines, opiates and tricyclic antidepressants, unless the results can be reliably attributed to a concomitant prescription medication by the PI for a condition identified on medical history, in which case the subject may be enrolled after consultation with and agreement of the Sponsor's medical monitor.
12. Have a positive urine screen for nicotine (urine cotinine test).
13. Have a positive breath test for alcohol at initial screening.
14. Have a positive serology test for HIV antibodies, HBsAg, or anti-HCV at screening.

15. Donated blood (volume ≥ 500 mL) within 56 days prior to screening.
16. Be unable or unwilling to undergo multiple venipunctures because of poor tolerability or lack of easy access to veins.
17. Received an experimental antibody or biologic therapy within the 6 months prior to screening, or received any other experimental therapy or new investigational agent within 60 days or 5 half-lives (whichever is longer) of study start.
18. Is an employee or employee family member of the investigator, study center, or Sponsor.
19. Any condition that, in the opinion of the investigator, would complicate or compromise the study, or the well-being of the subject.

5 STUDY SUBJECT ALLOCATION

Subjects will be assigned to 1 of 4 cohorts based on their current asthma status (healthy, mild, moderate, or severe asthma) according to the criteria described in Section 4.1 and Table A.2. All healthy subjects and approximately 30 of the 50 subjects in each of the asthma severity classifications will undergo a bronchoscopy procedure with endobronchial biopsies and bronchial brushings. The healthy subject group will be split into 2 age ranges of 18 to 36 years of age or 37 to 55 years of age. Asthma subjects undergoing bronchoscopy must be between 18 and 55 years of age, inclusive.

6 PRESTUDY AND CONCOMITANT THERAPY

All therapies administered for all subjects prior to this study must be recorded at screening.

Concomitant therapies must be recorded throughout the study beginning when the subject signs the consent form. All therapies (prescriptions or over-the-counter medications, including vitamins and herbal supplements; non-pharmacologic therapies such as electrical stimulation, acupuncture, special diets, exercise regimens) must be recorded in the concomitant therapy section of the CRF. Bronchodilators used to assess reversibility during pulmonary function testing must also be recorded in the concomitant therapy section of the CRF.

Moderate and severe asthmatic subjects are permitted to be on any asthma therapies at any time during the study. Modifications can be made to these therapies by their treating physician at any time during the study. Mild asthmatics must not be taking asthma controller medications eg, ICS at least 6 weeks prior to screening, but these medications may be started at any time during the study if the medical need arises. During the study, rescue medications, such as SABAs, are allowed. **Mild asthmatics must not have asthma controller therapies discontinued in order to fulfill inclusion/exclusion criteria for this study.**

7 STUDY EVALUATIONS

The Schedule of Events (Attachment 1) summarizes the frequency and timing of procedures, safety, genomic and other assessments applicable to this study. For screening, approximately 50 mL of blood will be required to complete hematology, serology and chemistry assessments. The estimated total blood volume to be collected at each scheduled venipuncture from each subject will be approximately 50 mL.

In addition, 1 blood sample will be collected from subjects who have signed a separate ICF agreeing to participate in the genomics component of the study. Analyses of collected samples will only be performed if it is deemed that this information may provide insight into the pathophysiology of asthma or other inflammatory diseases. Samples will also be stored for future research after the clinical study is completed (where local regulations permit).

Procedures will be performed at the times and order indicated in study procedures section of the Schedule of Events (Attachment 1). Asthma subjects should complete the ACQ and AQLQ questionnaires prior to completing any study procedures. When multiple procedures are performed at a given visit, the following guideline for order of procedures is recommended:

- ACQ
- AQLQ
- Physical Exam
- 12-lead ECG
- Vital signs

Every effort should be made to conduct the pulmonary assessment procedures in the following order:

- Exhaled nitric oxide
- Prebronchodilator and postbronchodilator spirometry (reversibility test).
- PC20 methacholine challenge
- Induced sputum

For further details on the order of procedures, see Study Reference Manual.

The following pulmonary procedures can be performed on the same day:

- Pre- and postbronchodilator spirometry (reversibility test) can be performed before sputum induction on the same day. The postbronchodilator FEV_1 from the reversibility test can be used as the pre-sputum induction FEV_1 baseline if the sputum induction is performed within 60 minutes.

- PC20 methacholine challenge may precede sputum induction on the same day. The postbronchodilator FEV_1 from the PC20 methacholine challenge test can be used as the pre- sputum induction FEV_1 baseline if the sputum induction is performed within 60 minutes.

Please note that the reversibility test (pre- and postbronchodilator spirometry) *cannot* be performed on the same day as PC20 methacholine challenge. However, the use of bronchodilators is permitted after the completion of the PC20 methacholine test.

The following assessments are to be performed after all pulmonary assessments are completed:

- Blood samples (excluding screening samples)
- Urinalysis (excluding screening samples)

7.1 Screening Procedures

Prior to screening, the investigator (or designee) will explain the study's rationale, procedures, and risks, and ask each potential subject if they wish to consent to participate. After written informed consent has been obtained, each subject will be assigned a unique subject number and will be evaluated for each of the inclusion/exclusion criteria outlined in Section 4.2 (healthy subjects) or Section 4.3 (asthmatic subjects). Subject screening can be conducted over a period of up to 21 days.

For healthy subjects, the induced sputum procedure should take place after the results of Phadiatop and other inclusion/exclusion criteria have been met and laboratory assessments have been received.

For asthma subjects the PC20 methacholine test, if required for eligibility, and induced sputum procedure will be performed after other inclusion/exclusion criteria and laboratory assessments have been received.

If a subject is not able to produce an acceptable sample of sputum, he/she may return for a second attempt during the screening period. The second sputum induction should occur no earlier than 48 hours post first attempt and during the 21 days of the screening period.

If the result of a screening assessment is abnormal, the assessment may be performed a second time at the discretion of the investigator and sponsor. In such cases, the first abnormal test result will not constitute a screen failure; however, a second clinically significant abnormal result will be considered a screen failure. A subject will not be enrolled in the study if results of tests performed at screening, or, if applicable, at the time of a second test indicate that the subject is ineligible to participate.

For subjects who are not eligible for this study due to screening virology serology test results, consultation with a physician with expertise in the treatment of such infections is recommended.

For asthmatic subjects who do not meet asthma inclusion/exclusion criteria (based on investigator and sponsor judgment and/or medication history) or do not meet the spirometric criteria, an additional assessment of asthma and/or spirometry can be done within 2 weeks of initial screening. In such cases, the first abnormal test result will not constitute a screen failure; however, a second clinically significant abnormal result will be considered a screen failure.

The assessments to be performed at the screening visit can be found in Attachment 1, Schedule of Events.

7.2 Study Procedures

Study procedures should be performed at the times noted and completed in the order indicated in the study procedures section of the Schedule of Events, Attachment 1.

7.2.1 Baseline Visit

A baseline visit should be scheduled within 5-14 days after the last screening procedure has been completed. The baseline procedures must be completed within a 7 day period. Subjects should not have had any acute illness in the 2 weeks prior to study visit, or the visit should be postponed until the subject can meet this prerequisite. If a subject cannot complete the baseline visit within 28 days of the end of screening, they will be withdrawn from the study. Subjects can be rescreened after the 28 day time period has elapsed and will be assigned a new subject number before continuing with the study.

Asthmatic subjects will be required to complete an asthma history questionnaire, an asthma quality of life questionnaire (AQLQ), and an asthma control questionnaire (ACQ).

Lung function in healthy subjects will be assessed using the reversibility test (pre- and post- bronchodilator spirometry). Lung function in asthmatic subjects will be assessed using fractional exhaled nitric oxide (FENO) and pre- and postbronchodilator spirometry (reversibility test). Baseline samples for biomarkers (induced sputum, blood and urine) will be collected. In addition, a blood sample for genomics analysis will be collected from subjects who provided consent. Asthmatic subjects will also need to complete a PC20 methacholine challenge if not performed at screening, but only if the subject's prebronchodilator FEV_1 is ≥ 60% of predicted normal value on the day of the challenge. Subjects who qualify for the study based on historical methacholine challenge data will also have a methacholine challenge test at the baseline visit. If the sputum sample is unacceptable, one additional attempt can be made to obtain sputum at least 48 hrs after the first attempt.

The assessments to be performed at the baseline visit can be found in Attachment 1, Schedule of Events.

7.2.2 Bronchoscopy Visit

A bronchoscopy and attendant procedures will be performed during this visit and should occur within 5 to 14 days after the baseline visit. Details of the bronchoscopy procedure are described in the Study Reference Manual.

Healthy subjects should meet the following criteria, or the bronchoscopy visit should be postponed until the subject can meet these prerequisites.

- No intercurrent illness in the 14 days prior to the bronchoscopy including upper respiratory tract infections, etc.
- Post-bronchodilator $FEV_1 \geq 80\%$ predicted that has not declined by more than 10% from screening. For example, if baseline FEV_1 is 3.86 liters, a decrease of ≥ 386 mL would render the subject ineligible for the bronchoscopy on that day.
- Healthy subjects who cannot complete the bronchoscopy visit within 14 days of the baseline visit will be given an additional 14 days to complete the procedure. If the subject could not complete the bronchoscopy after the additional 14 days they will be discontinued from the study.

Asthmatic subjects should not withhold any inhaled medications before the bronchoscopy visit. A pre- and post-bronchodilator reversibility test will be performed prior to the bronchoscopy. The postbronchodilator FEV_1 from this test serves as the prebronchoscopy FEV_1 baseline determining eligibility. Asthmatic subjects must meet the following criteria or the bronchoscopy visit should be postponed until the subject can meet these prerequisites.

- Must be between 18 and 55 years of age, inclusive, at informed consent.
- Clinically stable asthma for at least 14 days prior to the bronchoscopy procedure, as defined by:
  - no clinically significant worsening of asthma requiring augmentation of therapy (eg, increased use of rescue medications, antibiotics, systemic steroids, increased controller medications, hospitalization) as per judgment of the investigator,
- Post-bronchodilator $FEV_1 \geq 60\%$ predicted that has not declined by more than 10% from screening. For example, if baseline FEV_1 is 2.56 liters, a decrease of ≥ 256 mL would render the subject ineligible for the bronchoscopy on that day.
- No intercurrent illness in the 14 days prior to the bronchoscopy including upper respiratory tract infections etc.

Asthmatic subjects who cannot complete the bronchoscopy visit within 14 days of the baseline visit will be given an additional 14 days to complete the procedure. If an asthmatic subject cannot complete the bronchoscopy visit within 28 days of the baseline visit, that subject can be considered to be part of the non-bronchoscopy cohort. If the non-bronchoscopy cohort has already been completely enrolled, then the subject will be discontinued from the study.

Subjects receiving a bronchoscopy will be required to remain overnight in the study unit or at a suitable medical facility for follow-up if, in the opinion of the investigator, they are unfit for discharge based on symptoms, physical examination, vital signs, oximetry, and lung function parameters. In such a case, subjects should be treated with regular bronchodilators and any other treatments that may be medically indicated according to the judgment of the Investigator and undergo reassessment, including spirometry, before discharge the next day. Subjects will then be discharged based on the assessment of the PI.

Blood and urine samples for biomarker analysis will also be collected from all subjects undergoing bronchoscopy during this visit.

The assessments to be performed during the bronchoscopy visit can be found in Attachment 1, Schedule of Events.

7.2.3 Biomarker Visits (Asthmatic Subjects)

Additional visits are scheduled at 3, 6 and 12 months after the initial baseline visit for asthmatic subjects to monitor disease activity and should occur within ± 14 days of that time point. All biomarker visit procedures must be completed within a 7 day period. Subjects should not have had any acute illness in the 2 weeks prior to study visit or the visit should be postponed until the subject can meet this prerequisite. Each visit will include completion of ACQ and AQLQ, review of subject asthma event logs, lung function testing that includes bronchodilator reversibility, and collection of blood and urine for biomarker analysis. The 6-month visit will also include the collection of an induced sputum sample. If the sputum sample is not adequate, one additional attempt to obtain sputum should be scheduled at least 48 hrs after the first attempt.

If a subject is not available for a particular biomarker visit, the investigator should contact the sponsor medical monitor and discuss options for completing/rescheduling that visit.

The assessments to be performed during the biomarker visits can be found in Attachment 1, Schedule of Events.

7.3 Biomarker Evaluations

Biomarker assessments will include the evaluation of biomarkers in serum, urine, whole blood, endobronchial sampling, and induced sputum. Potential markers include, but are not limited to, tumor necrosis factor alpha (TNFα), interleukin-8 (IL-8), eotaxin, myeloperoxidase, matrix metalloprotease 2 (MMP2), MMP9, IL-4, IL-13, cysteinyl leukotrienes, 8-isoprostane, mRNA and microRNA (miRNA) differential expression profiles.

All samples will be taken according to the Schedule of Events. Instructions for the collection, processing, quality control and shipment of all biomarker samples are found in the Study Reference Manual.

7.3.1 Serum and Urine Biomarkers

Serum and urine will be obtained using routine collection procedures and assessed for cytokines, chemokines, MMPs, eicosanoids, and other markers that may be associated with asthma.

7.3.2 Whole Blood Gene Expression Profiling

Whole blood will be collected for RNA analysis to better understand the pathological mechanisms involved in asthma and the differential expression profiles of normal healthy subjects compared with those of mild, moderate and/or severe asthmatics.

7.3.3 Bronchoscopy (Endobronchial Biopsies and Brushings)

Bronchial brushings and endobronchial biopsies will be collected following a standardized procedure based on established guidelines and as described in the Study Reference Manual. Endobronchial biopsies and bronchial brushings will be analyzed for routine histology, immunohistochemistry, mRNA, miRNA and other biomarkers.

After the procedures, subjects will receive a short acting bronchodilator and spirometry will be performed. Discharge of subjects will be based on the principal investigator's clinical judgment taking the spirometry readings into account. Additional short-acting bronchodilators will be administered if required. All medication administered during the bronchoscopy procedure will be captured in the eCRF concomitant medication log. If the PI determines that a subject is not fit for discharge at 4 hours after the procedure, that subject will be kept overnight in a medical facility and be reassessed for discharge in the morning.

7.3.4 Induced Sputum

Induced sputum samples will be collected following a standardized procedure based on guidelines and recommendations and as described in the Study Reference Manual. All subjects will be required to have a post-bronchodilator FEV_1 of $\geq$ 60% predicted on the day of induction for induction with 3%, 4%, and 5% hypertonic saline. If post-bronchodilator FEV_1 is from $\geq$ 50% to < 60% predicted on the day of the induced

sputum visit, induction will be performed with normal (isotonic) 0.9% saline. If post-bronchodilator FEV_1 is < 50%, no sputum induction will be performed on that day and the visit should be rescheduled. An acceptable sputum sample requires a selected plug weight of at least 50 mg and < 20% squamous cells.

After the procedures, subjects will receive a short acting bronchodilator and spirometry will be performed. All medication administered during the induced sputum procedure will be captured in the eCRF concomitant medication log. Discharge of subjects will be based on the principal investigator's clinical judgment taking the spirometry readings into account. Additional short-acting bronchodilators will be administered if required. If the PI determines that a subject is not fit for discharge at 4 hours after the procedure, that subject will be kept overnight in a medical facility and be reassessed for discharge in the morning.

Sputum will be processed and both sputum supernatants and cell pellets will be analyzed for cell counts, RNA, and other biomarkers.

7.4 Spirometry and Bronchodilator Response

Throughout the study, it is preferable that for any individual subject at each study visit, PFTs are administered by the same pulmonologist or technician in order to ensure consistency of technique. All personnel administering PFTs must be trained according to local or institution-specific standard operating procedures (SOPs) before administering the tests to any subjects in the study. Confirmation of the training, as well as any updates, must be maintained at the study site.

FEV_1, FVC, forced expiratory flow (FEF25-75) and peak expiratory flow rate (PEFR) will be measured according to the Body Temperature, Pressure, Saturated standard (BTPS) convention with a spirometer that is suitable for research purposes, calibrated and maintained to acceptable respiratory function laboratory standards (Miller et al, 2005).

At all clinic visits, PFTs will be performed in the absence of albuterol/salbutamol (ie, at ≥ 6 hours after the last dose). In addition, post bronchodilator PFTs are performed 15 to 30 (± 5) minutes after administration of albuterol/salbutamol via metered dose inhaler (MDI) with a spacer or treatment with nebulized albuterol/salbutamol (albuterol/salbutamol reversibility) and % predicted FEV_1 will be calculated using previously established formulas (see Study Reference Manual). All medication administered during the bronchoscopy procedure will be captured in the eCRF concomitant medication log.

A detailed description of the lung function testing appears in the Study Reference Manual.

7.5 Methacholine Challenge

The methacholine challenge will not be performed if a subject has a pre-bronchodilator FEV_1 < 60% of predicted on the day of the test. Detailed instructions for conducting methacholine challenge test are described in Study Reference Manual.

A methacholine challenge test will be performed at screening in asthma subjects if needed in order to assess study eligibility. Asthma subjects who do not require the challenge test at screening will complete this assessment at their baseline visit.

In the methacholine challenge test, baseline spirometry for a subject is measured by inhalation of increasing concentrations of aerosolized methacholine up to 16 mg/mL. The provocative dose (PC20) is defined as the dose of methacholine required to cause a ≥ 20% decrease from the baseline FEV_1 value. A PC20 result of ≤ 16 mg/mL will be considered to reflect increase airway responsiveness for fulfilling eligibility criteria.

After the procedures, subjects will receive a short acting bronchodilator and spirometry will be performed. Discharge of subjects will be based on the principal investigator's clinical judgment taking the spirometry readings into account. Additional short-acting bronchodilators will be administered if required. If the PI determines that a subject is not fit for discharge at 4 hours after the procedure, that subject will be kept overnight in a medical facility and be reassessed for discharge in the morning. All medication administered during the bronchoscopy procedure will be captured in the eCRF concomitant medication log.

7.6 Fractional Exhaled Nitric Oxide (FENO)

Fractional exhaled nitric oxide will be measured for all asthmatic subjects at an exhalation flow rate of 50 mL/s according to ATS guidelines using an exhaled NO meter that is approved for asthma measurements. FENO testing must precede any other pulmonary procedures. Two replicate FENO measurements will be obtained that agree at the 10% level and up to a total of 8 measurements will be performed to achieve this level of agreement. The procedures for the collection of FENO are described in detail in Study Reference Manual.

7.7 Asthma Event Log

Asthmatic subjects will be provided with paper journals to cover periods between visits. The journal does not require an entry on days without symptoms, but in the event that the subject detects a change in respiratory status (eg, asthma exacerbation, ER visit, unscheduled clinic visit or hospitalization), they should note the date, time, precipitating event (if possible), and symptoms or activity limitation in the log. Any change in medication, use of prednisone, etc. should be recorded with start and stop dates. Additionally, if there are any new significant medical problems, eg, newly diagnosed diabetes, or acute medical problems such as pneumonia, appendicitis etc., these should also be recorded together with any new prescription medications.

The journal should be submitted to the study coordinator at the next study visit. A new paper journal will be provided to the study subjects at each visit.

7.8 Asthma Quality of Life Questionnaire (AQLQ)

Asthmatic subjects only will be asked to complete the Asthma Quality of Life Questionnaire, a 32 item, patient-reported outcome questionnaire to evaluate symptoms and several aspects of daily living in adult subjects (age 18+) with asthma (Juniper et al, 1999a; Juniper et al, 1993). The instrument uses a 7-point Likert scale to measure activity limitation and symptom frequency using a recall period of 2 weeks. The results are expressed as 4 domain scores (symptoms, activity limitation, emotional function, and environmental exposure) and an overall score. The generic activity limitation items were developed to represent a range of activity levels (sleeping to strenuous activity). A higher score represents better outcomes with less severe involvement. The AQLQ is self-administered and takes approximately 5 to 10 minutes to complete. The questionnaire content validity was established using patient interviews; developers also established construct and criterion validity; the resulting instrument has been used in clinical and research settings. Translations are available in several languages. The questionnaire will be provided in paper format to the study centers.

7.9 Asthma Control Questionnaire (ACQ)

Asthmatic subjects only will be asked to complete the Asthma Control Questionnaire, an instrument designed to evaluate asthma control defined as “the full range of clinical impairment that patients with asthma may experience as a result of the disease” (Juniper et al, 1999b). The control options cover a continuum from ‘well controlled’ to ‘life threatening’. The original version was developed with expert opinion, and patient participation in validation procedures. Items address recall of symptoms (night-time awakenings, morning symptoms, limitation of activities, shortness of breath, and wheezing) and SABA use during the previous week. In addition, the subject’s percent predicted FEV_1 value is scored. All 7 items are scored on a 7-point scale (0 = good control, 6 = poor control), with the mean score as an overall summary score; higher scores reflect poorer control. The recall period is 7 days. The questionnaire has strong measurement properties and has been widely used in research. The instrument is responsive to differences between stable and unstable asthma. Multiple translations are available.

A later version of the instrument was adapted for use as a daily diary (Juniper et al, 2000). Concordance between the original version and the diary version is high. The questionnaire will be provided in paper format to the study centers.

7.10 Asthma History Questionnaire

The asthma history questionnaire will be completed at the baseline visit in asthmatic subjects only, to provide details of the subject’s asthma history. The questionnaire will be provided in paper format to the study centers.

7.11 Safety Evaluations

Any procedure related clinically significant abnormalities persisting at the end of the study will be followed by the investigator until resolution or until a clinically stable endpoint is reached.

The study will include the following evaluations of safety and tolerability according to the time points provided in the Schedule of Events.

Adverse Events

Adverse events will be reported by the subject for the duration of the study. Adverse events will be followed by the investigator as specified in Section 10, Adverse Event Reporting.

Clinical Laboratory Tests

Blood samples for serum chemistry and hematology will be collected at screening and can be repeated if medically necessary during the study. The list of analyses to be completed is in Attachment 2. In addition to serum chemistry and hematology, the following assessments will also be performed:

- Urine Pregnancy Testing for all women participating in the study.
- Serology (HIV-1 antibodies, HBsAg, and anti-HCV antibodies).
- Urine Drug Screen (amphetamine, barbiturates, benzodiazepines, cannabinoids, cocaine, methadone, opiates, tricyclic antidepressants).
- Urine cotinine test.
- Alcohol breath test.

Electrocardiogram (ECG)

12-lead ECGs will be recorded at a paper speed of 25 mm per second until 4 regular consecutive complexes are available. The PI will review the ECG to ensure that there is no abnormality which would place the subject at risk from participation in the study.

Vital Signs

Measurement of supine vital signs (resting pulse rate, blood pressure, and temperature) will be performed after 10 minutes of rest at the timepoints specified in the study schedule of events (see Attachment 1). When scheduled concurrently with a blood draw or ECG, vital signs should be measured after the ECG and before the blood draw.

If any clinically significant changes in vital signs are noted and considered related to study procedures, they must be reported as AEs (see Section 10) and followed to resolution, or until reaching a clinically stable endpoint.

A detailed listing of vital signs to be evaluated will be provided in the Study Reference Manual.

Physical Examination

Physical examinations will be performed by the investigator or designated physician. The timepoints of these examinations are specified in the Schedule of Events (see Attachment 1).

Any abnormalities or changes in severity noted during the review of body systems should be documented in the source document and recorded on the CRF.

A new, clinically significant finding (in the opinion of the investigator) not noted at screening, and considered related to study procedures, must be captured as an AE (see Section 10). In addition, resolution of any abnormal findings during the study will be noted in the source document and in the CRF.

A detailed listing of body systems to be evaluated for initial physical exam and subsequent brief physical exams will be provided in the Study Reference Manual.

7.12 Genomic Evaluation

There are 2 parts to the genomic component of this study.

Subjects will be given the option to participate in Part 1 only, Part 2 only, both parts, or neither part of the genomic component of this study (where local regulations permit).

Analysis Related to the Study (Part 1)

Collection of genomic samples will allow for genetic research to help understand asthma. Genetic analysis will be conducted if it is hypothesized that this may help resolve issues with the clinical data. DNA samples will only be used for genetic research related to asthma. Genetic research may consist of the analysis of 1 or more candidate genes or of the analysis of genetic markers throughout the genome (as appropriate) in relation to asthma clinical endpoints. Subjects will be offered the separate option to consent to storage of their optional samples for future research as scientific discoveries are made.

DNA Storage for Future Research (Part 2)

Part 2 of the genomic research allows for the storage of DNA samples for future genetic research related to asthma. DNA samples will be stored for up to 5 years after the study is complete. Stored DNA samples and relevant clinical data will be de-identified after the Clinical Study Report has been issued. This involves removing personal identifiers and replacing the study subject identifier with a new number to limit the possibility of linking genetic data to a subject's identity.

Refusal to participate in genomics testing will not result in ineligibility for participation in the rest of the clinical study. Further, a subject may withdraw genomic consent at any time without affecting their participation in other aspects of the study.

8 SUBJECT COMPLETION/WITHDRAWAL

8.1 Completion

A healthy subject will be considered to have completed the study if he or she has completed the bronchoscopy procedure and telephone follow-ups on the day following bronchoscopy and 1 week after bronchoscopy. Asthmatic subjects will be considered to have completed the study if he or she completes the final biomarker visit. Subjects who prematurely discontinue study participation for any reason before completion of their final visit will not be considered to have completed the study.

8.2 Withdrawal From the Study

A subject will be withdrawn from the study for any of the following reasons:

- Lost to follow-up.
- Withdrawal of consent.
- Death.
- The investigator believes that for safety reasons (eg, adverse event) it is in the best interest of the subject to stop participation.
- The subject becomes pregnant.
- The study investigator or Sponsor for any reason stops the study or stops the subject's participation in the study.

Subjects who terminate study participation before their last scheduled visit should contact the investigational site if possible for a final visit. The Study coordinator should consult with the Sponsor as to what testing should be performed at that visit.

In the event a subject is lost to follow-up, every possible effort must be made by the study site personnel to contact the subject and determine the reason for discontinuation/withdrawal. The measures taken to follow up with a subject must be documented.

When a subject withdraws before completing the study, the reason for withdrawal is to be documented in the CRF and in the source document. Those subjects chosen to undergo bronchoscopy who withdraw prior to the bronchoscopy will be replaced with new subjects until up to 30 subjects from each subject category (healthy, mild, moderate or severe asthma) have undergone a bronchoscopy. Subjects who withdraw from the longitudinal sample collection portion of the study will not be replaced.

A subject who withdraws from the main part of the study will have the following options regarding genomic research:

- The DNA extracted from the subject's blood will be retained and used in accordance with the subject's original genomic informed consent.
- The subject may withdraw consent for genomic research, in which case the DNA sample will be destroyed and no further testing will take place. To initiate the sample destruction process, the investigator must notify the sponsor site contact to request sample destruction. The sponsor site contact will, in turn, contact the genomics representative to execute sample destruction. If requested, the investigator will receive written confirmation from the sponsor that the sample has been destroyed.

Withdrawal From Genomic Research Only

The subject may withdraw consent for genomic research while remaining in the clinical study. In such a case, any DNA extracted from the subject's blood will be destroyed. The sample destruction process will proceed as described above. However, all samples will be made nonidentifiable after the clinical study report is issued and thereafter cannot be identified for destruction. If the sample has already undergone conversion to the nonidentifiable format, the sponsor will notify the investigator in writing.

9 STATISTICAL METHODS

No formal hypothesis testing will be conducted. Data will be summarized using descriptive statistics. Continuous variables will be summarized using the number of observations, mean, SD, minimum, median, and maximum values as appropriate. Categorical values will be summarized using the number of observations and percentages as appropriate.

9.1 Subject Information

For all subjects who are enrolled in this study, descriptive statistics of demographic information and baseline characteristics will be provided.

9.2 Sample Size

This is an exploratory study. Therefore, no formal hypothesis is to be tested and no formal power calculation is performed in sample size determination.

The primary objective of this study is to explore asthma-associated biomarkers to aid in the evaluation of new and existing therapeutics for asthma and to understand the correlation between non-clinical models of disease and human disease. To perform this analysis, four groups are required: 3 asthma severity groups of subjects (mild, moderate, and severe) and a healthy control group.

The 3 asthma severity groups are required to:

- Identify biomarkers differentially expressed in asthmatics compared to healthy controls.
- Identify biomarkers differentially expressed among asthma severities.
- Identify subsets of asthmatics defined by biomarker expression profiles.

The healthy control group is required to:

- Identify biomarkers differentially expressed in asthmatics compared to healthy controls.
- Determine whether a biomarker profile expressed by a subset of asthmatics is specific for asthma or is also found in the healthy control population.

To estimate a suitable sample size of subjects to undergo bronchoscopy, the following was considered.

Based upon prior microarray data studies (Woodruff et al, 2007; Woodruff et al, 2009) and a previous clinical study of golimumab in subjects with asthma (unpublished results), the following assumptions can be made for determining the sample size:

1. Subsets comprising 20% of asthmatics and healthy controls can be identified from gene expression microarray analysis. A 20% subset is considered practical for targeting in personalized/stratified medicine approaches in asthma.
2. A minimum of 5 subjects would be necessary to be able to identify a biomarker-defined subset from hierarchical clustering analysis of gene expression microarray data.

Based on these assumptions, bronchoscopy samples from 25 subjects each for the 4 groups are needed to identify biomarker-defined subsets comprising at least 20% of subjects within a group. Assuming that samples adequate for analysis would be obtainable from 80% of subjects, a sample size of 30 subjects per group undergoing bronchoscopy is needed.

9.3 Interim Analyses

Two interim analyses are planned for this study.

The first interim analysis will be conducted on bronchoscopy samples and will be performed after the first 15 healthy subjects and the first 15 asthma subjects of at least one severity group have undergone bronchoscopy to determine whether to continue with additional bronchoscopy sampling. No formal database lock is required for this interim analysis. Any asthma severity group that has completed bronchoscopies in 15 subjects would be included in the analysis. Endobronchial biopsy specimens will be submitted for RNA extraction and gene expression microarray analysis to evaluate the level of variability within the cohort. If the analysis reveals that the median coefficient of

variation for probe-sets from gene expression microarray analysis exceeds 60% in both the asthma groups and the healthy control group, then additional bronchoscopies will not be conducted. In addition, if it is determined that the overall quality of the RNA obtained from bronchoscopy samples is inadequate, then additional bronchoscopies will not be conducted. While the interim analysis is being performed, the screening of subjects for bronchoscopies and performance of bronchoscopies will be stopped for subjects in the healthy cohort and the asthma severity group(s) included in the interim analysis. Enrollment of asthma subjects for bronchoscopy in the other severity groups will not exceed 15 subjects per group until the interim analysis is complete. Enrollment of asthma subjects not undergoing bronchoscopy will not be interrupted for the purpose of the interim analysis.

The second interim analysis will be conducted upon completion of last bronchoscopy procedures in the last subject participating in the bronchoscopy portion of the study. The main goal of this interim analysis will be biomarker evaluation of the samples collected at the screening, baseline, and bronchoscopy visits and will include but will not be limited to microarray analysis of RNA isolated from samples and soluble analyte analysis of serum and IS samples. To support this analysis a data base lock will be performed to provide demographic and clinical information for the planned biomarker analyses. The output of this interim analysis will include, but not be limited to, study population results, safety results, information on asthma characteristics of mild, moderate and severe cohorts and their respective clinical parameters, differences in biomarkers across cohorts, identification of biomarker-defined subsets across and within cohorts, correlations of biomarker, clinical, and demographic parameters, and correlations of biomarker measurements across sampling matrices.

9.4 Biomarker Data Analyses

The biomarker data and analyses will be reported in an independent technical report.

9.5 Safety Analyses

Adverse Events

In general, adverse events will be collected when these arise from study procedures (eg induced sputum, bronchoscopy, methacholine challenge test or blood draw). For an example, an asthma exacerbation that occurs after a bronchoscopy and that in the opinion of the investigator is related to the procedure should be recorded as an AE. The original terms used in the CRFs by investigators to identify adverse events will be coded using the MedDRA. All reported adverse events with onset during the study will be included in the analysis. All adverse events reported from both healthy subject and asthmatic subject populations will be listed.

Special attention will be given to those subjects who died, or who experienced a severe or a serious adverse event (eg, summaries, listings, and narrative preparation may be provided, as appropriate).

Vital Signs

Descriptive statistics of temperature, pulse and blood pressure (systolic and diastolic) values, and changes from baseline will be listed at each scheduled time point.

10 ADVERSE EVENT REPORTING

Timely, accurate, and complete reporting and analysis of safety information from clinical studies are crucial for the protection of subjects, investigators, and the sponsor, and are mandated by regulatory agencies worldwide. The sponsor has established SOPs in conformity with regulatory requirements worldwide to ensure appropriate reporting of safety information; all clinical studies conducted by the sponsor or its affiliates will be conducted in accordance with those procedures.

10.1 Definitions

10.1.1 Adverse Event Definitions and Classifications

Adverse Event

An adverse event is any untoward medical occurrence in a clinical study subject administered a medicinal (investigational or non-investigational) product or underwent a study procedure. An adverse event does not necessarily have a causal relationship with the treatment. An adverse event can therefore be any unfavorable and unintended sign (including an abnormal finding), symptom, or disease temporally associated with the use of a medicinal (investigational or non-investigational) product or study procedure, whether or not related to that medicinal (investigational or non-investigational) product or study procedure. (Definition per International Conference on Harmonisation [ICH]).

As no therapeutic intervention is involved with this study, only events temporally associated with a study procedure and determined to be possibly, probably or very likely related to a study procedure including spirometry, bronchoscopy, induced sputum, methacholine challenge and blood collection will be reported as adverse events.

This includes any occurrence that is new in onset or aggravated in severity or frequency from the baseline condition, or abnormal results of diagnostic procedures, including laboratory test abnormalities.

Note: The sponsor collects adverse events starting with the signing of the informed consent form (refer to Section 10.2.1, All Adverse Events, for time of last adverse event recording).

Serious Adverse Event

A serious adverse event as defined by ICH is any untoward medical occurrence that meets any of the following conditions:

- Results in death
- Is life-threatening
  (The subject was at risk of death at the time of the event. It does not refer to an event that hypothetically might have caused death if it were more severe.)
- Requires inpatient hospitalization or prolongation of existing hospitalization
- Results in persistent or significant disability/incapacity, or
- Is a congenital anomaly/birth defect

Note: Medical and scientific judgment should be exercised in deciding whether expedited reporting is also appropriate in situations other than those listed above. For example, important medical events may not be immediately life threatening or result in death or hospitalization but may jeopardize the subject or may require intervention to prevent one of the outcomes listed in the definition above (eg, suspected transmission of an infectious agent by a medicinal product is considered a serious adverse event). Any adverse event is considered a serious adverse event if it is associated with clinical signs or symptoms judged by the investigator to have a significant clinical impact.

10.1.2 Attribution Definitions

Not related

An adverse event that is not related to procedures in this study.

Doubtful

An adverse event for which an alternative explanation is more likely, eg, concomitant drug(s), concomitant disease(s), or the relationship in time suggests that a causal relationship to a study procedure is unlikely.

Possible

An adverse event that might be due to a study procedure. An alternative explanation, eg, concomitant drug(s), concomitant disease(s), is inconclusive. The relationship in time is reasonable; therefore, the causal relationship cannot be excluded.

Probable

An adverse event that might be due a study procedure. The relationship in time is suggestive. An alternative explanation is less likely, eg, concomitant drug(s), concomitant disease(s).

Very likely

An adverse event that is listed as a possible adverse reaction and cannot be reasonably explained by an alternative explanation, eg, concomitant drug(s), concomitant disease(s). The relationship in time is very suggestive (eg, it is confirmed by dechallenge and rechallenge).

10.1.3 Severity Criteria

An assessment of severity grade will be made using the following general categorical descriptors:

Mild: Awareness of symptoms that are easily tolerated causing minimal discomfort and not interfering with everyday activities.

Moderate: Sufficient discomfort is present to cause interference with normal activity.

Severe: Extreme distress causing significant impairment of functioning or incapacitation. Prevents normal everyday activities.

The investigator should use clinical judgment in assessing the intensity of events not directly experienced by the subject (eg, laboratory abnormalities).

10.2 Procedures

10.2.1 All Adverse Events

Only adverse events related to the study procedures, whether serious or non-serious, will be reported from the time a signed and dated informed consent form is obtained until completion of the last study-related procedure (may include contact for follow-up of safety). Serious adverse events, including those spontaneously reported to the investigator within 30 days after the last study procedure, must be reported using the Serious Adverse Event Form. The sponsor will evaluate any safety information that is spontaneously reported by an investigator beyond the time frame specified in the protocol.

All events related to study procedures that meet the definition of a serious adverse event will be reported as serious adverse events. Adverse events related to study procedures, regardless of seriousness or severity, must be recorded using medical terminology in the source document and the CRF. Whenever possible, diagnoses should be given when signs and symptoms are due to a common etiology (eg, cough, runny nose, sneezing, sore throat, and head congestion should be reported as “upper respiratory infection”). Investigators must record in the CRF their opinion concerning the relationship of the adverse event to study procedures. All measures required for adverse event management must be recorded in the source document and reported according to sponsor instructions.

The sponsor assumes responsibility for appropriate reporting of study procedure related adverse events to the regulatory authorities if required. The investigator (or sponsor where required) must report these events to the appropriate Independent Ethics Committee/Institutional Review Board (IEC/IRB) that approved the protocol unless otherwise required and documented by the IEC/IRB.

Subjects (or their designees, if appropriate) must be provided with a “study card” indicating the study number, the investigator’s name and a 24-hour emergency contact number.

10.2.2 Serious Adverse Events

All serious adverse events occurring during clinical studies must be reported to the appropriate sponsor contact person by investigational staff within 24 hours of their knowledge of the event.

Information regarding serious adverse events will be transmitted to the sponsor using the Serious Adverse Event Form, which must be completed and signed by a member of the investigational staff, and transmitted to the sponsor within 24 hours. The initial and follow-up reports of a serious adverse event should be made by facsimile (fax).

All serious adverse events that have not resolved by the end of the study, or that have not resolved upon discontinuation of the subject’s participation in the study, must be followed until any of the following occurs:

- The event resolves
- The event stabilizes
- The event returns to baseline, if a baseline value is available
- The event can be attributed to factors other than study procedures or study conduct
- It becomes unlikely that any additional information can be obtained (subject or health care practitioner refusal to provide additional information, lost to follow-up after demonstration of due diligence with follow-up efforts)

The cause of death of a subject in a clinical study, whether or not the event is expected or associated with a study required procedure, is considered a serious adverse event. Any event requiring hospitalization (or prolongation of hospitalization) that occurs during the course of a subject’s participation in a clinical study must be reported as a serious adverse event, except hospitalizations for the following:

- Social reasons in absence of an adverse event
- Surgery or procedure planned before entry into the study (must be documented in the CRF)

10.2.3 Pregnancy

Any subject who becomes pregnant must be promptly withdrawn from the study and no further study procedures should be performed.

10.3 Contacting Sponsor Regarding Safety

The names (and corresponding telephone numbers) of the individuals who should be contacted regarding safety issues or questions regarding the study are listed on the Contact Information page(s), which will be provided as a separate document.

11 ETHICAL ASPECTS

11.1 Study-Specific Design Considerations

None of the procedures may necessarily benefit the subjects and there is the potential of adverse events in particular related to the procedures. For asthmatic subjects, it is possible that knowledge of the inflammatory phenotype of the subjects could help their physician guide future therapy.

For subjects who are undergoing sputum induction, common expected effects include salty taste, retching, vomiting coughing, dizziness and faintness. Most of these are mild and self-limited. However, depending on the severity of these symptoms, the PI may decide to record an AE. Subjects, particularly those with asthma may experience dyspnea, wheeze due to bronchospasm. Again, in most cases the symptoms are mild and self-limiting. The PI will decide as to whether such asthma symptoms require recording as an adverse event on a case-by-case basis. The overall risk of sputum induction is low and there are no reported cases of death (see Study Reference Manual for safety monitoring). If a subject has an unnoticed infectious disease (eg, active TB), there may be a risk of exposing study personnel to infection.

The bronchoscopy procedure consists of the administration of lidocaine, atropine and agents used for conscious sedation and the procedure itself. There are potential adverse events that arise from lidocaine including lidocaine toxicity which is dose related and can be prevented by recording the amounts administered and setting an upper limit (see Study Reference Manual). Atropine may result in tachycardia, worsening of glaucoma, and urinary retention. Subjects should be screened for these conditions before bronchoscopy. The medications used for conscious sedation vary from center to center but include opiates and short-acting intravenous benzodiazepines. The risks of these agents include allergic reactions, excessive sedation with respiratory depression and hypoxemia, hypercarbia and apnea. Careful titration of medications and monitoring of all subjects by skilled personnel including continuous ECG and oximetry should help avoid these complications.

The bronchoscopy procedure includes insertion of the scope via a naris or the oral cavity and hence through the vocal cords into the lower respiratory tree. While an extremely

safe procedure, complications can occur that, in some cases, may be serious and even result in death. A non-exhaustive list includes nasal pain, epistaxis, retching, vomiting and aspiration, vocal cord spasm, bleeding from the lower airways, bronchospasm, hypoxemia and pneumothorax. Death, while rare, can occur due to bleeding or asphyxia particularly in those with asthma. Careful screening of subjects and skilled operators and precautions as mandated in the Study Reference Manual will help reduce the chance that these procedures are harmful.

Potential subjects will be fully informed of the risks and requirements of the study and, during the study, subjects will be given any new information that may affect their decision to continue participation. They will be told that their consent to participate in the study is voluntary and may be withdrawn at any time with no reason given and without penalty or loss of benefits to which they would otherwise be entitled. Only subjects who are fully able to understand the risks, benefits, and potential adverse events of the study, and provide their consent voluntarily will be enrolled.

11.2 Regulatory Ethics Compliance

11.2.1 Investigator Responsibilities

The investigator is responsible for ensuring that the clinical study is performed in accordance with the protocol, current ICH guidelines on GCP, and applicable regulatory and country-specific requirements.

Good Clinical Practice is an international ethical and scientific quality standard for designing, conducting, recording, and reporting studies that involve the participation of human subjects. Compliance with this standard provides public assurance that the rights, safety, and well being of study subjects are protected, consistent with the principles that originated in the Declaration of Helsinki, and that the clinical study data are credible.

11.2.2 Independent Ethics Committee or Institutional Review Board (IEC/IRB)

Before the start of the study, the investigator (or sponsor where required) will provide the IEC/IRB with current and complete copies of the following documents:

- Final protocol and, if applicable, amendments
- Sponsor-approved informed consent form (and any other written materials to be provided to the subjects)
- Sponsor-approved subject recruiting materials
- Information on compensation for study-related injuries or payment to subjects for participation in the study, if applicable
- Investigator's curriculum vitae or equivalent information (unless not required, as documented by IEC/IRB)

- Information regarding funding, name of the sponsor, institutional affiliations, other potential conflicts of interest, and incentives for subjects
- Any other documents that the IEC/IRB requests to fulfill its obligation

This study will be undertaken only after the IEC/IRB has given full approval of the final protocol, amendments (if any), the informed consent form, applicable recruiting materials, and subject compensation programs, and the sponsor has received a copy of this approval. This approval letter must be dated and must clearly identify the IEC/IRB and the documents being approved.

Approval for the genomic research component of the clinical study and for the genomic informed consent form must be obtained from the IEC/IRB. Approval for the protocol can be obtained independent of approval for genomic research.

During the study the investigator (or sponsor where required) will send the following documents and updates to the IEC/IRB for their review and approval, where appropriate:

- Protocol amendments
- Revision(s) to informed consent form and any other written materials to be provided to subjects
- If applicable, new or revised subject recruiting materials approved by the sponsor
- Revisions to compensation for study-related injuries or payment to subjects for participation in the study, if applicable
- Summaries of the status of the study at intervals stipulated in guidelines of the IEC/IRB (at least annually)
- Reports of adverse events that are serious, unlisted/unexpected, and associated with a study procedure
- New information that may adversely affect the safety of the subjects or the conduct of the study
- Deviations from or changes to the protocol to eliminate immediate hazards to the subjects
- Report of deaths of subjects under the investigator's care
- Notification if a new investigator is responsible for the study at the site
- Annual Safety Report and Line Listings, where applicable
- Any other requirements of the IEC/IRB

For all protocol amendments (excluding the ones that are purely administrative, with no consequences for subjects, data or trial conduct), the amendment and applicable informed

consent form revisions must be submitted promptly to the IEC/IRB for review and approval before implementation of the change(s).

At least once a year, the IEC/IRB will be asked to review and reapprove this clinical study. The reapproval should be documented in writing (excluding the ones that are purely administrative, with no consequences for subjects, data or trial conduct).

At the end of the study, the investigator (or sponsor where required) will notify the IEC/IRB about the study completion.

11.2.3 Informed Consent

Each subject must give written consent according to local requirements after the nature of the study has been fully explained. The consent form must be signed before performance of any study-related activity. The consent form that is used must be approved by both the sponsor and by the reviewing IEC/IRB. The informed consent should be in accordance with principles that originated in the Declaration of Helsinki, current ICH and GCP guidelines, applicable regulatory requirements, and sponsor policy.

Before enrollment in the study, the investigator or an authorized member of the investigational staff must explain to potential subjects the aims, methods, reasonably anticipated benefits, and potential hazards of the study, and any discomfort participation in the study may entail. Subjects will be informed that their participation is voluntary and that they may withdraw consent to participate at any time. They will be informed that choosing not to participate will not affect the care the subject will receive for the treatment of his or her disease. Finally, they will be told that the investigator will maintain a subject identification register for the purposes of long-term follow-up if needed and that their records may be accessed by health authorities and authorized sponsor staff without violating the confidentiality of the subject, to the extent permitted by the applicable law(s) or regulations. By signing the informed consent form the subject is authorizing such access, and agrees to allow his or her study physician to recontact the subject for the purpose of obtaining consent for additional safety evaluations, if needed.

The subject will be given sufficient time to read the informed consent form and the opportunity to ask questions. After this explanation and before entry into the study, consent should be appropriately recorded by means of the subject's personally dated signature. After having obtained the consent, a copy of the informed consent form must be given to the subject.

Subjects will be asked to consent to participate in a genomic research component of the study (where local regulations permit). After informed consent for the clinical study is appropriately obtained, the subject will be asked to sign and personally date a separate genomic informed consent form indicating agreement to participate in optional genomic

research. A copy of the signed genomic informed consent form will be given to the subject. Refusal to participate will not result in ineligibility for the clinical study.

If the subject is unable to read or write, an impartial witness should be present for the entire informed consent process (which includes reading and explaining all written information) and should personally date and sign the informed consent form after the oral consent of the subject is obtained.

Subjects who are unable to comprehend the information provided can be enrolled only after obtaining consent of a legally-acceptable representative. Written assent should be obtained from subjects who are able to write. After having obtained the assent, a copy of the assent form must be given to the subject, and the subject's legally-acceptable representative.

When prior consent of the subject is not possible, enrollment procedures should be described in the protocol with documented approval/favorable opinion by the IEC/IRB to protect the rights, safety, and well being of the subject and to ensure compliance with applicable regulatory requirements. The subject or legally-acceptable representative must be informed about the study as soon as possible and give consent to continue.

11.2.4 Privacy of Personal Data

The collection and processing of personal data from subjects enrolled in this study will be limited to those data that are necessary to fulfill the objectives of the study.

These data must be collected and processed with adequate precautions to ensure confidentiality and compliance with applicable data privacy protection laws and regulations. Appropriate technical and organizational measures to protect the personal data against unauthorized disclosures or access, accidental or unlawful destruction, or accidental loss or alteration must be put in place. Sponsor personnel whose responsibilities require access to personal data agree to keep the identity of study subjects confidential.

The informed consent obtained from the subject (or his or her legally-acceptable representative) includes explicit consent for the processing of personal data and for the investigator to allow direct access to his or her original medical records for study-related monitoring, audit, IEC/IRB review, and regulatory inspection (if applicable). This consent also addresses the transfer of the data to other entities and to other countries.

The subject has the right to request through the investigator access to his or her personal data and the right to request rectification of any data that are not correct or complete. Reasonable steps will be taken to respond to such a request, taking into consideration the nature of the request, the conditions of the study, and the applicable laws and regulations.

For those subjects who gave consent to store DNA samples for future genetic research, samples and corresponding relevant clinical data will be made nonidentifiable by the removal of personal identifiers. Samples will be stored until completely used. Genotypic data generated on nonidentifiable samples cannot be returned to individual subjects.

11.2.5 Long-Term Storage of Samples for Future Research

After publication of the final study report, samples and corresponding relevant clinical data will be made nonidentifiable by the removal of personal identifiers. Samples will be stored up to 5 years after completion of the study report. Only research related to asthma will be done on stored samples.

12 ADMINISTRATIVE REQUIREMENTS

12.1 Protocol Amendments

Neither the investigator nor the sponsor will modify this protocol without a formal amendment. All protocol amendments must be issued by the sponsor, and signed and dated by the investigator. Protocol amendments must not be implemented without prior IEC/IRB approval, or when the relevant competent authority has raised any grounds for non-acceptance, except when necessary to eliminate immediate hazards to the subjects, in which case the amendment must be promptly submitted to the IEC/IRB and relevant competent authority. Documentation of amendment approval by the investigator and IEC/IRB must be provided to the sponsor or its designee. When the change(s) involves only logistic or administrative aspects of the study, the IRB (and IEC where required) only needs to be notified.

During the course of the study, in situations where a departure from the protocol is unavoidable, the investigator or other physician in attendance will contact the appropriate sponsor representative (see Contact Information pages provided separately). Except in emergency situations, this contact should be made before implementing any departure from the protocol. In all cases, contact with the sponsor must be made as soon as possible to discuss the situation and agree on an appropriate course of action. The data recorded in the CRF and source documents will reflect any departure from the protocol, and the source documents will describe this departure and the circumstances requiring it.

12.2 Regulatory Documentation

12.2.1 Regulatory Approval/Notification

As this study does not involve an investigational agent, it is anticipated that regulatory approval will not be required. In the event that regulatory approval is required in a given country, this protocol and any amendment(s) must be submitted to the appropriate regulatory authorities in each respective country. A study may not be initiated until all local regulatory requirements are met.

12.2.2 Required Prestudy Documentation

The following documents must be provided to the sponsor before study initiation at the investigational site:

- Protocol and amendment(s), if any, signed and dated by the investigator
- A copy of the dated and signed written IEC/IRB approval of the protocol, amendments, informed consent form, any recruiting materials, and if applicable, subject compensation programs. This approval must clearly identify the specific protocol by title and number and must be signed by the chairman or authorized designee.
- Name and address of the IEC/IRB including a current list of the IEC/IRB members and their function, with a statement that it is organized and operates according to GCP and the applicable laws and regulations. If accompanied by a letter of explanation, or equivalent, from the IEC/IRB, a general statement may be substituted for this list. If an investigator or a member of the investigational staff is a member of the IEC/IRB, documentation must be obtained to state that this person did not participate in the deliberations or in the vote/opinion of the study.
- Regulatory authority approval or notification, if applicable
- Signed and dated statement of investigator (eg, Form FDA 1572), if applicable
- Documentation of investigator qualifications (eg, curriculum vitae)
- Completed investigator financial disclosure form from the investigator, where required
- Signed and dated clinical trial agreement, which includes the financial agreement
- Any other documentation required by local regulations

The following documents must be provided to the sponsor before screening of the first subject:

- Completed investigator financial disclosure forms from all clinical subinvestigators, where required
- Documentation of subinvestigator qualifications (eg, curriculum vitae)
- Photocopy of the site signature log, describing delegation of roles and responsibilities at the start of the study
- Name and address of any local laboratory conducting tests for the study, and a dated copy of current laboratory normal ranges for these tests
- Local laboratory documentation demonstrating competence and test reliability (eg, accreditation/license), if applicable.

12.3 Subject Identification, Enrollment, and Screening Logs

The investigator agrees to complete a subject identification and enrollment log to permit easy identification of each subject during and after the study. This document will be reviewed by the sponsor site contact for completeness.

The subject identification and enrollment log will be treated as confidential and will be filed by the investigator in the study file. To ensure subject confidentiality, no copy will be made. All reports and communications relating to the study will identify subjects by initials and assigned number only.

The investigator must also complete a subject-screening log, which reports on all subjects who were seen to determine eligibility for inclusion in the study.

12.4 Source Documentation

At a minimum, source documentation must be available for the following to confirm data collected in the CRF: subject identification, eligibility, and study identification; study discussion and date of informed consent; dates of visits; results of safety and efficacy parameters as required by the protocol; record of all adverse events; and follow-up of adverse events; concomitant medication; and date of study completion, and reason for withdrawal from the study, if applicable.

It is recommended that the author of an entry in the source documents be identifiable.

At a minimum, the type and level of detail of source data available for a study subject should be consistent with that commonly recorded at the site as a basis for standard medical care. Specific details required as source data for the study will be reviewed with the investigator before the study and will be described in the monitoring guidelines (or other equivalent document).

12.5 Case Report Form Completion

Case report forms are provided for each subject in electronic format. Electronic data capture (eDC) will be used for this study. The study data will be transcribed by study personnel from the source documents onto an electronic CRF, and transmitted in a secure manner to the sponsor within 2 days of the subject's visit. The electronic file will be considered to be the CRF. Worksheets may be used for the capture of some data to facilitate completion of the CRF. Any such worksheets will become part of the subjects' source documentation. All data relating to the study must be recorded in CRFs prepared by the sponsor. Data must be entered into CRFs in English. Designated site personnel must complete CRFs as soon as possible after a subject visit, and the forms should be available for review at the next scheduled monitoring visit.

Every effort should be made to ensure that all subjective measurements (eg questionnaires) to be recorded in the CRF are completed by the same individual who

made the initial baseline determinations. The investigator must verify that all data entries in the CRFs are accurate and correct.

All CRF entries, corrections, and alterations must be made by the investigator or other authorized study-site personnel. If necessary, queries will be generated in the eDC tool. The investigator or an authorized member of the investigational staff must adjust the CRF (if applicable) and complete the query.

If corrections to a CRF are needed after the initial entry into the CRF, this can be done in 3 different ways:

- Site personnel can make corrections in the eDC tool at their own initiative or as a response to an auto query (generated by the eDC tool).
- Site manager (SM) can generate a query (field DCF) for resolution by the investigational staff.
- Clinical data manager (CDM) can generate a query for resolution by the investigational staff.

12.6 Data Quality Assurance/Quality Control

Steps to be taken to ensure the accuracy and reliability of data include the selection of qualified investigators and appropriate study centers, review of protocol procedures with the investigator and associated personnel before the study, and periodic monitoring visits by the sponsor, and direct transmission of clinical laboratory data from a central laboratory into the sponsor's data base. Written instructions will be provided for collection, preparation, and shipment of blood, plasma, and urine samples.

Guidelines for CRF completion will be provided and reviewed with study personnel before the start of the study. The sponsor will review CRFs for accuracy and completeness during on-site monitoring visits and after transmission to the sponsor; any discrepancies will be resolved with the investigator or designee, as appropriate. After upload of the data into the clinical study database they will be verified for accuracy.

12.7 Record Retention

In compliance with the ICH/GCP guidelines, the investigator/institution will maintain all CRFs and all source documents that support the data collected from each subject, as well as all study documents as specified in ICH/GCP Section 8, Essential Documents for the Conduct of a Clinical Trial, and all study documents as specified by the applicable regulatory requirement(s). The investigator/institution will take measures to prevent accidental or premature destruction of these documents.

Essential documents must be retained until at least 2 years after the issuance of a final approved clinical study report for this study. These documents will be retained for a

longer period if required by the applicable regulatory requirements or by an agreement with the sponsor. It is the responsibility of the sponsor to inform the investigator/institution as to when these documents no longer need to be retained.

If the responsible investigator retires, relocates, or for other reasons withdraws from the responsibility of keeping the study records, custody must be transferred to a person who will accept the responsibility. The sponsor must be notified in writing of the name and address of the new custodian. Under no circumstance shall the investigator relocate or dispose of any study documents before having obtained written approval from the sponsor.

If it becomes necessary for the sponsor or the appropriate regulatory authority to review any documentation relating to this study, the investigator must permit access to such reports.

12.8 Monitoring

The sponsor will perform on-site monitoring visits as frequently as necessary. The monitor will record dates of the visits in a study center visit log that will be kept at the site. The first post-initiation visit will be made as soon as possible after enrollment has begun (when the first subject successfully completes the screening period) or in accordance with monitoring guideline, whichever occurs first. At these visits, the monitor will compare the data entered into the CRFs with the hospital or clinic records (source documents). The nature and location of all source documents will be identified to ensure that all sources of original data required to complete the CRF are known to the sponsor and investigational staff and are accessible for verification by the sponsor site contact. If electronic records are maintained at the investigational site, the method of verification must be discussed with the investigational staff.

Direct access to source documentation (medical records) must be allowed for the purpose of verifying that the data recorded in the CRF are consistent with the original source data. Findings from this review of CRFs and source documents will be discussed with the investigational staff. The sponsor expects that, during monitoring visits, the relevant investigational staff will be available, the source documentation will be accessible, and a suitable environment will be provided for review of study-related documents. The monitor will meet with the investigator on a regular basis during the study to provide feedback on the study conduct.

12.9 Study Completion/Termination

12.9.1 Study Completion

The study is considered completed with the last visit of the last subject participating in the study. The final data from the investigational site will be sent to the sponsor (or designee) after completion of the final subject visit at that site, in the time frame specified in the Clinical Trial Agreement.

12.9.2 Study Termination

The sponsor reserves the right to close the investigational site or terminate the study at any time for any reason at the sole discretion of the sponsor. Investigational sites will be closed upon study completion. An investigational site is considered closed when all required documents and study supplies have been collected and a site closure visit has been performed.

The investigator may initiate site closure at any time, provided there is reasonable cause and sufficient notice is given in advance of the intended termination.

Reasons for the early closure of an investigational site by the sponsor or investigator may include but are not limited to:

- Failure of the investigator to comply with the protocol, the sponsor's procedures, or GCP guidelines
- Inadequate recruitment of subjects by the investigator

12.10 On-Site Audits

Representatives of the sponsor's clinical quality assurance department may visit the site at any time during or after completion of the study to conduct an audit of the study in compliance with regulatory guidelines and company policy. These audits will require access to all study records, including source documents, for inspection and comparison with the CRFs. Subject privacy must, however, be respected. The investigator and staff are responsible for being present and available for consultation during routinely scheduled site audit visits conducted by the sponsor or its designees.

Similar auditing procedures may also be conducted by agents of any regulatory body, either as part of a national GCP compliance program or to review the results of this study in support of a regulatory submission. The investigator should immediately notify the sponsor if they have been contacted by a regulatory agency concerning an upcoming inspection.

12.11 Use of Information and Publication

All information, including but not limited to information regarding this study or the sponsor's operations (eg, patent application, formulas, manufacturing processes, basic scientific data, prior clinical data, formulation information) supplied by the sponsor to the investigator and not previously published, and any data, including genomic research data, generated as a result of this study, are considered confidential and remain the sole property of the sponsor. The investigator agrees to maintain this information in confidence and use this information only to accomplish this study, and will not use it for other purposes without the sponsor's prior written consent.

The investigator understands that the information developed in the clinical study will be used by the sponsor in connection with the development of new medicines, and thus may be disclosed as required to other clinical investigators or regulatory agencies. To permit the information derived from the clinical studies to be used, the investigator is obligated to provide the sponsor with all data obtained in the study.

The results of the study will be reported in a Clinical Study Report generated by the sponsor and will contain CRF data from all investigational sites that participated in the study. Recruitment performance or specific expertise related to the nature and the key assessment parameters of the study will be used to determine a coordinating investigator. Results of biomarker and genomic analyses performed after the Clinical Study Report has been issued will be reported in a separate report and will not require a revision of the Clinical Study Report. Study subject identifiers will not be used in publication of genomic results. Any work created in connection with performance of the study and contained in the data that can benefit from copyright protection (except any publication by the investigator as provided for below) shall be the property of the sponsor as author and owner of copyright in such work.

The sponsor shall have the right to publish such data and information without approval from the investigator. If an investigator wishes to publish information from the study, a copy of the manuscript must be provided to the sponsor for review at least 60 days before submission for publication or presentation. Expedited reviews will be arranged for abstracts, poster presentations, or other materials. If requested by the sponsor in writing, the investigator will withhold such publication for up to an additional 60 days to allow for filing of a patent application. In the event that issues arise regarding scientific integrity or regulatory compliance, the sponsor will review these issues with the investigator. The sponsor will not mandate modifications to scientific content and does not have the right to suppress information. For multicenter study designs and substudy approaches, results may need to be published in a given sequence (eg, substudies) should not generally be published before the primary endpoints of a study have been published. Similarly, investigators will recognize the integrity of a multicenter study by not publishing data derived from the individual site until the combined results from the completed study have been published in full, within 12 months after conclusion, abandonment, or termination of the study at all sites, or the sponsor confirms there will be no multicenter study publication. Authorship of publications resulting from this study will be based on the guidelines on authorship, such as those described in the Uniform Requirements for Manuscripts Submitted to Biomedical Journals, which state that the named authors must have made a significant contribution to the design of the study or analysis and interpretation of the data, provided critical review of the paper, and given final approval of the final version.

Registration of Clinical Studies and Disclosure of Results

The sponsor will register and/or disclose the existence of and the results of clinical studies as required by law.

13 REFERENCES

Barnes PJ. Immunology of asthma and chronic obstructive pulmonary disease. *Nat Rev Immunol*. 2008;8:183-192.

Chu HW, Kraft M, Rex MD, Martin RJ. Evaluation of Blood Vessels and Edema in the Airways of Asthma Patients. *Chest*. 2001;120:416-422.

Huang SK, Peters-Golden M. Eicosanoid lipid mediators in fibrotic lung diseases. Ready for prime time? *Chest.* 2008;133:1442-1450.

Juniper EF, Buist AS, Cox FM, Ferrie PJ, King DR. Validation of a standardized version of the Asthma Quality of Life Questionnaire. *Chest*. 1999a;115(5):1265-1270.

Juniper EF, Guyatt GH, Ferrie PJ, Griffith LE. Measuring quality of life in asthma. *Am Rev Respir Dis*. 1993;147(4):832-838.

Juniper EF, O'Byrne PM, Ferrie PJ, King DR, Roberts JN. Measuring asthma control. Clinic questionnaire or daily diary? *Am J Respir Crit Care Med*. 2000;162(4 Pt 1):1330-4.

Juniper EF, O'Byrne PM, Guyatt GH, Ferrie PJ, King DR. Development and validation of a questionnaire to measure asthma control. *Eur Respir J*. 1999b;14(4):902-907.

Linzer JF. Review of Asthma: Pathophysiology and Current Treatment Options. *Clin Pediatr Emerg Med*. 2007;8:87-95.

Maddox L, Schwartz DA. The Pathophysiology of Asthma. *Ann Rev Med*. 2002;53:477-498.

Miller MR, Hankinson J, Brusasco V, et al. Standardisation of spirometry. *Eur Respir J*. 2005;26:319-338.

National Institutes of Health. Clinical Practice Guidelines: Expert Panel Report 2: Guidelines for the Diagnosis and Management of Asthma. NHLBI: *NIH publication 97-4051*, July 1997.

National Institutes of Health. Clinical Practice Guidelines: Expert Panel Report 3: Guidelines for the Diagnosis and Management of Asthma. NHLBI: *NIH publication 08-4051*, 2007.

Rogers DF, Evans TW. Plasma exudation and oedema in asthma. *Br Med Bull*. 1992;48(1):120-134.

Rogers DF. Airway mucus hypersecretion in asthma: an undervalued pathology? *Current Opin Pharmacol*. 2004;4:241–250.

Tiddens H, Silverman M, Bush A. The Role of Inflammation in Airway Disease Remodeling. *Am J Respir Crit Care Med*. 2000;162:S7–S10.

Toews GB. Cytokines and the lung. *Eur Respir J.* 2001;18: Suppl. 34:3s–17s.

Woodruff PG, Boushey HA, Dolganov GM, et al. Genome-wide profiling identifies epithelial cell genes associated with asthma and with treatment response to corticosteroids. *Proc Nat Acad Sci USA*. 2007;104(40):15858-15863.

Woodruff PG, Modrek B, Choy DF, et al. T-helper Type 2–driven Inflammation Defines Major Subphenotypes of Asthma. *Am J Respir Crit Care Med*. 2009;180:388–395.

Yoshihara S, Yamada Y, Abe T, Lindén A, Arisaka O. Association of epithelial damage and signs of neutrophil mobilization in the airways during acute exacerbations of paediatric asthma. *Clin Exp Immunol*. 2006;144:212–216.

ATTACHMENT 1: SCHEDULE OF EVENTS

Attachment 1.1 Study Assessments for Healthy Subjects

Assessments	Screen[a]	Baseline[b] 5 - 14 days after screening	Bronchoscopy[c] 5 -14 days after baseline
Screening/Administrative/Other Procedures			
Informed consent	X		
Demography/history	X		
Eligibility criteria	X	X[d]	X[d]
Medical and medication history	X		
Drug/alcohol screen[e]	X		
Serology[f, g]	X		
Phadiatop test[g, h]	X		
Safety Procedures			
Physical exam	X		
Brief physical exam		X	X
Vital signs[i]	X	X	X
Height	X		
Weight	X		
BMI	X		
Supine 12-Lead ECG	X		
Urine pregnancy test[j]	X	X	X
Serum chemistry[f]	X		
Hematology[f]	X		
PT/aPTT	X		
Urinalysis[f]	X		
Adverse event monitoring[k]	X---X		
Monitor for concomitant medications	X---X		
Study Procedures			
Spirometry prebronchodilator	X[l]	X	X[o]
Spirometry postbronchodilator	X[l]	X[m, n]	X[o]
Induced sputum[p]	X	X	
Fiberoptic bronchoscopy			X[q]
Serum samples for biomarkers		X	X
Urine sample for biomarkers		X	X
Whole blood sample for RNA		X	
Blood sample for genomics[r]		X	
Telephone follow-up	X[s]	X[s]	X[s]

a. Screening can be conducted over a period of 21 days.
b. The baseline procedures can be conducted over a period of 7 days.
c. Subjects who cannot complete the bronchoscopy visit within 14 days of the baseline visit will be given an additional 14 days to complete the procedure. If the subject could not complete the bronchoscopy after the additional 14 days they will be discontinued from the study.
d. Review eligibility and safety criteria for the induced sputum and bronchoscopy procedures.
e. Includes amphetamines, barbiturates, benzodiazepines, cannabinoids, cocaine, methadone, opiates, tricyclic antidepressants, a screen for urine cotinine and alcohol breath test.
f. See Attachment 2 for the list of serologic, serum chemistry, hematologic and urine laboratory tests to be completed.
g. Blood draw for serology and Phadiatop test can be performed when the blood draw for serum chemistry, hematology and PT/aPTT is being done.
h. Phadiatop test will be used for subject exclusion in the healthy cohort only.

Attachment 1.1 Study Assessments for Healthy Subjects

i. Supine vital signs should be measured after 10 minutes of rest and include temperature, resting pulse rate, and blood pressure. When scheduled concurrently with a blood draw or ECG, vital signs should be measured after the ECG and before the blood draw.
j. Pregnancy test will be performed for all female subjects prior to study procedures.
k. Only adverse events related to study procedures should be recorded.
l. Healthy subjects will be excluded if the pre-bronchodilator FEV_1 value is < 85% of predicted value, and bronchodilator response is ≥ 12% and at least 200 mL.
m. The post bronchodilator FEV_1 value obtained at this visit serves as eligibility criterion for induced sputum if the induced sputum procedure is performed within 60 minutes. This FEV_1 value also serves as the pre-induction FEV_1 baseline and as a reference for post procedure FEV_1 recovery value.
n. Post bronchodilator spirometry will be performed at this visit if subjects' post procedure FEV_1 does not return to 80% of pre induction FEV_1 baseline. (see Study Reference Manual).
o. Pre- and postbronchodilator FEV_1 values are assessed before bronchoscopy and the postbronchodilator FEV_1 serves as the prebronchoscopy baseline for eligibility and safety monitoring. After the bronchoscopy, a short-acting bronchodilator may be administered and spirometry is repeated (see Study Reference Manual).
p. If a subject does not produce a valid sputum sample at screening, they may be given a second opportunity to do so within the screening window (21 days). Sputum Induction may be repeated at the baseline visit, if required, within the 7 day window for this visit. The second sputum induction should occur no earlier than 48 hours from first attempt for both the screening and baseline visits.
q. Subjects will be required to remain overnight in the study unit for follow-up if, based on the clinical judgment of the Investigator, a subject is unfit for discharge. Subjects will undergo reassessment, including spirometry, the following day and will be discharged based on the Investigator's assessment of the subject's well being. See Section 7.2.2 and the Study Reference Manual for follow-up procedures.
r. Requires an additional informed consent from the subject.
s. Telephone contact should be made on the day after sputum induction and both 1 and 7 days after bronchoscopy to collect any procedure related adverse events.

Attachment 1.2 Study Assessments for Asthmatic Subjects Receiving a Bronchoscopy

Assessments	Visit					
	**Screen**[a]	**Baseline**[b] **5 - 14 days after screening**	**Bronchoscopy 5 – 14 days**[c] **after baseline**	**Biomarkers 3 months**[d] **after baseline**	**Biomarkers**[b] **6 months**[d] **after baseline**	**Biomarkers 12 months**[d] **after baseline**
Screening/Administrative/Other Procedures						
Informed consent	X					
Demography/history	X					
Eligibility criteria	X	X[e]	X[e]		X[e]	
Medical and medication history	X					
Drug/alcohol screen[f]	X					
Serology[g,h]	X					
Phadiatop test[h,i]		X				
Safety Procedures						
Physical exam	X					
Brief physical exam		X	X	X	X	X
Vital signs[j]	X	X	X	X	X	X
Height	X					
Weight	X					
BMI	X					
Supine 12-Lead ECG	X					
Urine pregnancy test[k]	X	X	X	X	X	
Serum chemistry[g]	X					
Hematology[g]	X					
PT/aPTT	X					
Urinalysis[g]	X					
Adverse event monitoring[l]	X---X					
Monitor for concomitant medications	X---X					
Study Procedures						
Asthma diary and medical event log[m]	X---X					
AQLQ, ACQ		X		X	X	X
Asthma history questionnaire		X				
FENO		X		X	X	X

Attachment 1.2 Study Assessments for Asthmatic Subjects Receiving a Bronchoscopy

Assessments	Visit					
	Screen[a]	Baseline[b] 5 - 14 days after screening	Bronchoscopy 5 – 14 days[c] after baseline	Biomarkers 3 months[d] after baseline	Biomarkers[b] 6 months[d] after baseline	Biomarkers 12 months[d] after baseline
Spirometry prebronchodilator	X[n]	X[n]	X	X[n]	X[n]	X[n]
Spirometry postbronchodilator	X	X	X	X	X	X
PC20 methacholine[o]	X[p,q]	X[q,r]				
Induced sputum	X[s,t]	X[s,t]			X[r]	
Fiberoptic bronchoscopy			X[u]			
Serum samples for biomarkers		X	X	X	X	X
Urine sample for biomarkers		X	X	X	X	X
Whole blood sample for RNA		X		X	X	X
Blood sample for genomics[v]		X				
Telephone follow-up	X[x]	X[w,x]	X[w,y]		X[w,x]	

a. Screening can be conducted over a period of 21 days.
b. The baseline and 6-month biomarkers procedures can be conducted over a period of 7 days.
c. Asthmatic subjects who cannot complete the bronchoscopy visit within 14 days of the baseline visit will be given an additional 14 days to complete the procedure. If an asthmatic subject cannot complete the bronchoscopy visit within 28 days of the baseline visit, that subject can be considered to be part of the non-bronchoscopy cohort. If the non-bronchoscopy cohort has already been completely enrolled, then the subject will be discontinued from the study.
d. These visits should be conducted ± 14 days of the scheduled time.
e. Review eligibility and safety criteria for the induced sputum and bronchoscopy procedures (see Sections 4.3.1 and 7.2.2).
f. Includes amphetamines, barbiturates, benzodiazepines, cannabinoids, cocaine, methadone, opiates, tricyclic antidepressants, a screen for urine cotinine and alcohol breath test.
g. See Attachment 2 for the list of serologic, serum chemistry, hematologic and urine laboratory tests to be completed.
h. Blood draw for serology and Phadiatop test can be performed when the blood draw for serum chemistry, hematology and PT/aPTT is being done.
i. Phadiatop test is completed at baseline visit as it is not an inclusion criterion for asthmatics.
j. Supine vital signs should be measured after 10 minutes of rest and include temperature, resting pulse rate, and blood pressure. When scheduled concurrently with a blood draw or ECG, vital signs should be measured after the ECG and before the blood draw.
k. Pregnancy test will be performed for all female subjects prior to study procedures.
l. Only adverse events related to study procedures should be recorded.
m. Asthma diary and medical event log should be filled out continuously from screening through the bronchoscopy visit and for 2 weeks prior to each biomarker visit.

Attachment 1.2 Study Assessments for Asthmatic Subjects Receiving a Bronchoscopy

Assessments	Visit					
	Screen[a]	Baseline[b] 5 - 14 days after screening	Bronchoscopy 5 – 14 days[c] after baseline	Biomarkers 3 months[d] after baseline	Biomarkers[b] 6 months[d] after baseline	Biomarkers 12 months[d] after baseline

n. Some asthma medications are withheld prior to reversibility testing – pre and post bronchodilator FEV_1 response. For a detailed list of medications, see Study Reference Manual.
o. If prebronchodilator FEV_1 is < 60%, PC20 methacholine should not be done. PC20 methacholine challenge test cannot be performed on the same day as reversibility testing.
p. PC20 methacholine is done at screening if FEV_1 bronchodilator response is < 12% and no documented reversibility or PC20 is available (see Sections 4.3.1 and 7.5).
q. Some asthma medications are withheld prior to PC20 methacholine test. For detailed list of medications, see Study Reference Manual.
r. PC20 methacholine test is performed at the baseline visit if not conducted at the screening visit.
s. If a subject does not produce a valid sputum sample at screening, they may be given a second opportunity to do so within the screening window (21 days). Sputum induction may also be repeated at the baseline and 6-month biomarker Visit if required within the 7 day window for this visit. The second sputum induction should occur no earlier than 48 hours from first attempt for the screening baseline and biomarker visits.
t. Sputum induction may be performed on the same day as PC20 methacholine test with the PC20 methacholine test being conducted first followed by induced sputum. The postbronchodilator FEV_1 obtained after the PC20 methacholine test can serve as a pre sputum induction baseline value if sputum induction occurs within 60 minutes. This pre induction baseline FEV_1 value serves to determine eligibility for sputum induction, which strength of saline should be used, and for safety monitoring (see Study Reference Manual).
u. Asthmatic subjects will be required to remain overnight in the study unit for follow-up if, based on the clinical judgment of the Investigator, a subject is unfit for discharge. Subjects will undergo reassessment, including spirometry, the following day and will be discharged based on the Investigator’s assessment of the subject’s well being See Section 7.2.2 and the Study Reference Manual for follow-up procedures.
v. Requires an additional informed consent from the subject.
w. The study coordinator should contact the subject by telephone 7 days and 24 hours before the baseline, bronchoscopy and 6 month biomarker visits to ensure that subject’s asthma is well controlled before the visit.
x. Telephone contact should be made the day after the sputum induction procedure to collect any procedure related adverse events.
y. Telephone contact should be made 1 and 7 days after the bronchoscopy visit to collect any procedure related adverse events.

Attachment 1.3 Study Assessments for Asthmatic Subjects Not Receiving a Bronchoscopy

Assessment	Visit				
	Screen[a]	Baseline[b] 5 - 14 days after screening	Biomarkers 3 months[c] after baseline	Biomarkers[b] 6 months[c] after baseline	Biomarkers 12 months[c] after baseline
Screening/Administrative/Other Procedures					
Informed consent	X				
Demography/history	X				
Eligibility criteria	X	X[d]		X[d]	
Medical and medication history	X				
Drug/alcohol screen[e]	X				
Serology[f,g]	X				
Phadiatop test[g,h]		X			
Safety Procedures					
Physical exam	X				
Brief physical exam		X	X	X	X
Vital signs[i]	X	X	X	X	X
Height	X				
Weight	X				
BMI	X				
Supine 12-Lead ECG	X				
Urine pregnancy test[j]	X	X	X	X	
Serum chemistry[f]	X				
Hematology[f]	X				
PT/aPTT	X				
Urinalysis[f]	X				
Adverse event monitoring[k]	X--X				
Monitor for concomitant medications	X--X				
Study Procedures					
Asthma diary and medical event log[l]	X--X				
AQLQ, ACQ		X	X	X	X
Asthma history questionnaire		X			
FENO		X	X	X	X

Attachment 1.3 Study Assessments for Asthmatic Subjects Not Receiving a Bronchoscopy

Assessment	Visit				
	Screen[a]	Baseline[b] 5 - 14 days after screening	Biomarkers 3 months[c] after baseline	Biomarkers[b] 6 months[c] after baseline	Biomarkers 12 months[c] after baseline
Spirometry prebronchodilator	X[m]	X[m]	X[m]	X[m]	X[m]
Spirometry postbronchodilator	X	X	X	X	X
PC20 methacholine[n]	X[o,p]	X[p,q]			
Induced sputum	X[r,s]	X[r,s]		X[r]	
Serum samples for biomarkers		X	X	X	X
Urine sample for biomarkers		X	X	X	X
Whole blood sample for RNA		X	X	X	X
Blood sample for genomics[t]		X			
Telephone follow-up	X[u]	X[u,v]		X[u,v]	

a. Screening can be conducted over a period of 21 days.
b. The baseline and 6-month biomarker procedures can be conducted over a period of 7 days.
c. These visits may be conducted within ± 14 days of the scheduled time.
d. Review eligibility and safety criteria for the induced sputum procedures (see Section 4.3.1).
e. Includes amphetamines, barbiturates, benzodiazepines, cannabinoids, cocaine, methadone, opiates, tricyclic antidepressants, a screen for urine cotinine and alcohol breath test.
f. See Attachment 2 for the list of serologic, serum chemistry, hematologic and urine laboratory tests to be completed.
g. Blood draw for serology and Phadiatop test can be performed when the blood draw for serum chemistry, hematology and PT/aPTT is being done.
h. Phadiatop test is completed at baseline visit as it is not an inclusion criterion for asthmatics.
i. Supine vital signs should be measured after 10 minutes of rest and include temperature, resting pulse rate, and blood pressure. When scheduled concurrently with a blood draw or ECG, vital signs should be measured after the ECG and before the blood draw.
j. Pregnancy test will be performed for all female subjects prior to study procedures.
k. Only adverse events related to study procedures should be recorded.
l. Asthma diary and medical event log should be filled out continuously from screening through the baseline visit and for 2 weeks prior to each biomarker visit.
m. Some asthma medications are withheld prior to reversibility testing – pre and post bronchodilator FEV_1 response. For a detailed list of medications, see Study Reference Manual.
n. If prebronchodilator FEV_1 is < 60%, PC20 methacholine should not be done. PC20 methacholine challenge test cannot be performed on the same day as reversibility testing.

Attachment 1.3 Study Assessments for Asthmatic Subjects Not Receiving a Bronchoscopy
o. PC20 methacholine is done at screening if FEV_1 bronchodilator response is < 12% and no documented reversibility or PC20 is available (see Sections 4.3.1 and 7.5).<br>p. Some asthma medications are withheld prior to PC20 methacholine test. For detailed list of medications, see Study Reference Manual.<br>q. PC20 methacholine test is performed at the baseline visit if not conducted at the screening visit.<br>r. If a subject does not produce a valid sputum sample at screening, they may be given a second opportunity to do so within the screening window (21 days). Sputum induction may also be repeated at the baseline and 6-month biomarker Visit if required within the 7 day window for this visit. The second sputum induction should occur no earlier than 48 hours from first attempt for the screening baseline and biomarker visits.<br>s. Sputum induction may be performed on the same day as PC20 methacholine test with the PC20 methacholine test being conducted first followed by induced sputum. The postbronchodilator FEV_1 obtained after the PC20 methacholine test can serve as a pre sputum induction baseline value if sputum induction occurs within 60 minutes. This pre induction baseline FEV_1 value serves to determine eligibility for sputum induction, which strength of saline should be used, and for safety monitoring (see Study Reference Manual).<br>t. Requires an additional informed consent from the subject.<br>u. Telephone contact should be made the day after the sputum induction procedure to collect any procedure related adverse events.<br>v. The study coordinator should contact the subject by telephone 7 days and 24 hours before the baseline, bronchoscopy and 6 month biomarker visits to ensure that subject's asthma is well controlled before the visit.

ATTACHMENT 2: CENTRAL LABORATORY ASSESSMENTS

Urine Drug Screen	Serology	Urinalysis	Hematology	Serum Chemistry
• Amphetamine<br>• Barbiturates<br>• Benzodiazepines<br>• Cannabinoids<br>• Cocaine<br>• Methadone<br>• Opiates<br>• Tricyclic antidepressants	• HIV-1 screen<br>• HBsAg<br>• Anti-HCV	Dipstick:<br>• Specific gravity<br>• pH<br>• Glucose<br>• Protein<br>• Blood<br>• Ketones<br>• Bilirubin<br>• Urobilinogen<br>• Nitrite<br>• Leukocyte esterase	• Hemoglobin<br>• Hematocrit<br>• RBC<br>• WBC with differential<br>• Lymphocytes<br>• Monocytes<br>• Neutrophils<br>• Bands[b]<br>• Eosinophils<br>• Basophils<br>• Platelets	• Albumin<br>• Alkaline phosphate<br>• ALT<br>• AST<br>• Bicarbonate<br>• Total bilirubin<br>• Direct bilirubin[a]<br>• BUN<br>• Calcium<br>• Chloride<br>• CK<br>• CK-MB[c]<br>• Troponin-I[c]<br>• Creatinine<br>• serum glucose<br>• LDH<br>• Phosphate, inorganic<br>• Potassium<br>• Total protein<br>• Sodium
	Other Assessments		Coagulation	
	• Urine pregnancy test[e]<br>• Phadiatop test<br>• Alcohol breath test[e]<br>• Urine cotinine test		• PTT<br>• aPTT	

a Assay if total bilirubin is elevated above normal range.
b If immature neutrophils are detected, the sample is to be flagged, and a blood slide for microscopic analysis will be made. If Bands are detected in the microscopic analysis, then a result will be provided.
c Assess if CK is elevated above normal range.
d If dipstick result is abnormal, flow cytometry will be used to measure sediment. In case of discordance between the dipstick results and the flow cytometric results, the sediment will be examined microscopically.
e. Test to be conducted at the study center.

ATTACHMENT 3 PROTOCOL HISTORY

Original Protocol: 12 Apr 2010

Amendment 1: 17 Nov 2010

Amendment 2: 22 Apr 2011

Amendment 3: 29 May 2012

AMENDMENT 1 - 17 NOV 2010

The protocol has been revised to incorporate this amendment, which applies to all study centers. The rationale and description of the changes to the protocol are provided below. Minor formatting and typographical errors have also been corrected to improve the clarity and accuracy of the protocol text; these changes do not affect the overall conduct of the study.

1. Substantial changes were made throughout the protocol in inclusion/exclusion criteria, asthma classification definitions, order of pulmonary procedures, timing and conduct of procedures, and to the schedule of events.

These changes were deemed necessary based on Investigator input during study start-up and regulatory review to further clarify the conduct of the study.

Sections Affected	Original Content	Amended/New Content
3 OVERVIEW OF STUDY DESIGN	Figure 1	Figure 1 was updated.
3.1 Study Design Rationale (paragraph 2, sentence 4)	If hyperreactivity to methacholine cannot be performed at screening due to a low FEV_1 or subject does not have a historical data to document hyperreactivity, then, an obstructive airway deficit demonstrated by a FEV_1/FVC ratio, may be used to support the diagnosis.	If hyperreactivity to methacholine cannot be performed at screening due to a low FEV_1 or subject does not have a historical data to document hyperreactivity, then, an obstructive airway deficit demonstrated by a FEV_1/FVC ratio **of ≤ 0.7**, may be used to support the diagnosis.
4.1 General Considerations (paragraph 2)	All asthma severities as defined by the National Heart, Lung, and Blood Institute (NHLBI) (Appendix A) are eligible for this study (NIH, 1997). Dose levels below are also defined by the NHLBI (Appendix B) (NIH, 2007). Approximately 50 subjects will be recruited in each of the three asthma categories (mild, moderate and severe) and eligibility will be determined by the recruitment status in each category. The categories are defined as:	The National Heart, Lung, and Blood Institute (NHLBI) **expert panel** (NIH, 1997) **provided a guideline for asthma subjects classification into respective asthma severity groups (Table A.1,** Appendix A). **Medication** dose levels **used for the treatment of asthma** are also defined by the NHLBI (Appendix B) (NIH, 2007). Approximately 50 subjects will be recruited in each of the three asthma categories (mild, moderate, severe) **as defined below. Note that not all asthmatic subjects will qualify for this study (see Table A.2, Appendix A).** The categories are defined as:

(bullet 2, sub-bullet 3)	- Medium dose inhaled corticosteroid (ICS), or low to medium dose ICS/long acting β-2 agonist (LABA) or low to medium dose ICS and leukotriene antagonist (LTRA) modifiers, theophylline, or other controller (NHLBI STEP 2 or 3; Appendix A).	- **Low to** medium dose inhaled corticosteroid (ICS) **alone**, or low to medium dose ICS **in combination with any other controller medication (eg** LABA, leukotriene antagonist (LTRA), theophylline, **etc) with the exception of oral corticosteroids (OCS) and omalizumab** (NHLBI STEP 3 or 4; Appendix A).
(bullet 3, sub-bullet 3)	- High dose ICS/LABA, or high-dose ICS and other controller(s) including but not limited to LTRA modifiers, theophylline, anticholinergics, oral corticosteroid (OCS), anti-IgE (NHLBI STEP 4, 5 or 6; Appendix A).	- High dose ICS **alone or in combination with any other controller medication (eg LABA**, LTRA, theophylline, anticholinergics, OCS, anti-IgE, **etc)** (NHLBI STEP 5 or 6; Appendix A).
4.2.1 Inclusion Criteria (bullet 6)<br>4.3.1 Inclusion Criteria for Asthmatic Subjects (bullet 6)	• Must be able to produce an adequate induced sputum sample at screening (> 1 mL and < 80% squamous cells).	• Must be able to produce an adequate induced sputum sample at screening, **defined as a selected plug weight of at least 50 mg and a squamous cell count of < 20% (see Study Reference Manual).**
4.2.2 Exclusion Criteria (bullet 4)	• Have a bronchodilator response of >12% or an FEV1 value ≤ 85% of predicted value at screening.	• Have a bronchodilator response of **≥ 12% and 200 mL from baseline** or an FEV_1 value < 85% of predicted value at screening.
4.2.2 Exclusion Criteria (bullet 9),<br>4.3.2Exclusion Criteria for Asthmatic Subjects (bullet 9)	• Positive urine toxicology screen for substances of abuse, including but not limited to alcohol, cocaine, cannabinoids, phencyclidine, amphetamines, benzodiazepines, barbiturates, opiates, propoxyphene, and methadone.	• Positive urine toxicology screen for substances of abuse, including but not limited to amphetamines, barbiturates, benzodiazepines, cannabinoids, cocaine, methadone, opiates and tricyclic antidepressants.
4.2.2 Exclusion Criteria (bullet 11),<br>4.3.2 Exclusion Criteria for Asthmatic Subjects (bullet 11)	None	• **Have a positive breath test for alcohol at initial screening visit.**
4.2.2 Exclusion Criteria (bullet 15),<br>4.3.2 Exclusion Criteria for Asthmatic Subjects (bullet 15)	• Received an experimental antibody or biologic therapy within the previous 6 months before screening, or received any other experimental therapy or new investigational agent within 60 days or 5 half-lives (whichever is longer) of study start.	• Received an experimental antibody or biologic therapy within the 6 months **prior to screening**, or received any other experimental therapy or new investigational agent within 60 days or 5 half-lives (whichever is longer) of study **start**.

4.3.1 Inclusion Criteria for Asthmatic Subjects (bullet 3)	• Male or female between 18 and 70 years of age, inclusive.	• Be between 18 and 70 years of age, inclusive**, at informed consent. Subjects who elect to undergo bronchoscopy must be between 18 and 55 years old, inclusive, at informed consent.**
(bullet 10)	None	• **Fall into 1 of the categories for asthma severity (mild, moderate, or severe) as described in the study population (see Section 4.1).**
(bullet 11)	None	• **Prebronchodilator** FEV_1 **$\geq$ 50% predicted at screening.**
(bullet 12)	None	• **Must have clinically stable asthma (see Section 7.2.2) for at least 6 weeks prior to screening.**
(bullet 14)	None	• **Have been on their current asthma controller therapy for at least 6 weeks prior to screening. Mild asthmatics must have been off asthma controller therapy for at least 6 weeks prior to screening. Addition or withdrawal of asthma controller medications in order to be considered for participation in the study is prohibited.**
(bullet 15, sub-bullets 3 and 5)	- Clinically stable asthma for at least 14 days prior to the bronchoscopy procedure, as defined by:<br>- No acute illness in the interval between the baseline visit and bronchoscopy procedure.	Removed
(bullet 15, sub-bullet 1)	- Must be $\leq$ 55 years of age.	- Must be **between 18 and** 55 years of age, **inclusive, at informed consent.**
(bullet 15, sub-bullet 4)	- Post-bronchodilator FEV1 > 60% predicted and has not declined by more than 10% from screening.	- Post-bronchodilator $FEV_1 \geq 60\%$ predicted at screening.

4.3.2 Exclusion Criteria for Asthmatic Subjects<br>(bullet 2, sentence 2)	None	**Subjects with stable, well-controlled conditions may be eligible after consultation with the Sponsor's medical monitor.**
(bullet 3)	None, original bullet 3 is now bullet 4)	• **Diagnosis of allergic bronchopulmonary aspergillosis (ABPA), allergic bronchopulmonary mycosis (ABPM), or occupational asthma.**
(bullet 15)	• Received an experimental antibody or biologic therapy within the previous 6 months, or received any other experimental therapy or new investigational agent within 60 days or 5 half-lives (whichever is longer) of study.	• Received an experimental antibody or biologic therapy within the 6 months **prior to screening**, or received any other experimental therapy or new investigational agent within 60 days or 5 half-lives (whichever is longer) of study **start**.
5 STUDY SUBJECT ALLOCATION<br>(paragraph 1)	Subjects will be assigned to 1 of 4 cohorts based on their current asthma status (healthy, mild, moderate or severe asthma). All healthy subjects and approximately 30 of 50 in each of the asthma severity classifications will receive a bronchoscopy with endobronchial biopsies and bronchial brushings. The healthy subject cohort will be split equally into two age ranges of 18 to 36 years of age or 37 to 55 years of age.	Subjects will be assigned to 1 of 4 cohorts based on their current asthma status (healthy, mild, moderate, or severe asthma) **according to the criteria described in Section 4.1 and Table A.2**. All healthy subjects and approximately 30 of the 50 **subjects** in each of the asthma severity classifications will **undergo** a bronchoscopy **procedure** with endobronchial biopsies and bronchial brushings. The healthy subject **group** will be split equally into 2 age ranges of 18 to 36 years of age or 37 to 55 years of age. **Asthma subjects undergoing bronchoscopy must be between 18 and 55 years of age, inclusive.**
6 PRESTUDY AND CONCOMITANT THERAPY<br>(paragraph 2, sentence 3)	new sentence inserted	**Bronchodilators used to assess reversibility during pulmonary function testing must also be recorded in the concomitant therapy section of the CRF.**
(paragraph 3, sentences 3 and 4)	None	**Mild asthmatics must not be taking asthma controller medications eg, ICS at least 6 weeks prior to screening, but these medications may be started at any time during the study if the medical need arises. During the study, rescue medications, such as SABAs, are allowed.**

7 STUDY EVALUATIONS (sentence after bullet 4)	None	**Every effort should be made to conduct the pulmonary assessment procedures in the following order:**
(bullet 6)	• Spirometry	• **Prebronchodilator and postbronchodilator spirometry (reversibility test).**
(bullet 9)	• Blood samples	Deleted
(bullet 10)	• Urinalysis	Deleted
(rest of section)	The post-bronchodilator FEV1 measurement, if performed, will be the baseline spirometry reading for the sputum induction procedure. Alternatively, spirometry and sputum induction may be performed on separate days over a two-day period. Sputum induction may be performed with hypertonic saline in subjects with FEV1 ≥ 60%. For subjects with FEV1 ≥ 50 and ≤ 59%, sputum induction should be performed using normal saline. Sputum induction should not be done on asthmatic subjects with an FEV1 value < 50% of predicted values.	**For further details on the order of procedures, see Study Reference Manual.**<br>**The following pulmonary procedures can be performed on the same day:**<br>• **Pre- and postbronchodilator spirometry (reversibility test) can be performed before sputum induction on the same day. The postbronchodilator FEV_1 from the reversibility test can be used as the pre-sputum induction FEV_1 baseline if the sputum induction is performed within 60 minutes.**<br>• **PC20 methacholine challenge may precede sputum induction on the same day. The postbronchodilator FEV_1 from the PC20 methacholine challenge test can be used as the pre- sputum induction FEV_1 baseline if the sputum induction is performed within 60 minutes.**<br>**Please note that the reversibility test (pre- and postbronchodilator spirometry) cannot be performed on the same day as PC20 methacholine challenge. However, the use of bronchodilators is permitted after the completion of the PC20 methacholine test.**<br>**The following assessments are to be performed after all pulmonary assessments are completed:**

		• **Blood samples (excluding screening samples)**<br>• **Urinalysis (excluding screening samples)**
7.1 Screening Procedures (paragraph 1, sentence 3)	If a PC20 methacholine challenge is to be completed during screening, it should be completed within 5 days following initial pulmonary function screening. Induced sputum should be done within 5 days of initial screening and after screening laboratory assessment results are available.	**Subject screening can be conducted over a period of up to 21 days.**
(paragraphs 2, 3 and 4)	None. New paragraphs 2, 3 and 4 were inserted after paragraph 1.	**For healthy subjects, the induced sputum procedure should take place after the results of Phadiatop and other inclusion/exclusion criteria have been met and laboratory assessments have been received.**<br>**For asthma subjects the PC20 methacholine test, if required for eligibility, and induced sputum procedure will be performed after other inclusion/exclusion criteria and laboratory assessments have been received.**<br>**If a subject is not able to produce an acceptable sample of sputum, he/she may return for a second attempt during the screening period. The second sputum induction should occur no earlier than 48 hours post first attempt and during the 21 days of the screening period.**
(paragraph 6)	None. New paragraph inserted.	**For subjects who are not eligible for this study due to screening virology serology test results, consultation with a physician with expertise in the treatment of such infections is recommended.**

7.2.1 Baseline Visit (heading)	7.2.1 Baseline Visit (Within 14 Days of Screening)	7.2.1 Baseline Visit
(paragraphs 1 and 2)	A baseline visit should be completed within 14 days of screening completion. Subjects should not have experienced any acute illness in the 2 weeks prior to study visit or the visit should be postponed until the subject can meet this prerequisite. If a subject cannot complete the baseline visit within 28 days of the end of screening, they will need to be rescreened before continuing with the study. During this visit, screening results will be reviewed to ensure continued subject eligibility. Asthmatic subjects will be required to complete an asthma history questionnaire, an asthma quality of life questionnaire (AQLQ) and an asthma control questionnaire (ACQ).	A baseline visit should be **scheduled** within **5-14** days **after the last** screening **procedure has been completed**. **The baseline procedures must be completed within a 7 day period.** Subjects should not have **had** any acute illness in the 2 weeks prior to study visit or the visit should be postponed until the subject can meet this prerequisite. If a subject cannot complete the baseline visit within 28 days of the end of screening, they will **be withdrawn from the study. Subjects can be rescreened after the 28 day time period has elapsed and will be assigned a new subject number before continuing with the study.**<br><br>Asthmatic subjects will be required to complete an asthma history questionnaire, an asthma quality of life questionnaire (AQLQ) and an asthma control questionnaire (ACQ).
(paragraph 3)	Lung functions will be assessed using fractional exhaled nitric oxide (FENO), spirometry and bronchodilatory response. Baseline samples for biomarkers (induced sputum, blood and urine) will be collected. In addition, a blood sample for genomics analysis will be collected from subjects who provided consent. Asthmatic subjects will also need to complete a PC20 methacholine challenge within 5 days of baseline visit if not required at screening.	**Lung function in healthy subjects will be assessed using the reversibility test (pre- and post- bronchodilator spirometry).** Lung **function in asthmatic subjects** will be assessed using fractional exhaled nitric oxide (FENO) **and pre- and postbronchodilator** spirometry **(reversibility test)**. Baseline samples for biomarkers (induced sputum, blood and urine) will be collected. In addition, a blood sample for genomics analysis will be collected from subjects who provided consent. Asthmatic subjects will also need to complete a PC20 methacholine **challenge if not performed at screening, but only if the subject's prebronchodilator FEV_1 is ≥ 60% of predicted normal on the day of the challenge. Subjects who qualify for the study based on historical methacholine challenge data will also have a methacholine challenge test at the baseline visit. If the sputum sample is unacceptable, one additional attempt can be made to obtain sputum at least 48 hrs after the first attempt.**

7.2.2 Bronchoscopy Visit (heading)	7.2.2 Bronchoscopy Visit (7-14 Days After Baseline Visit)	7.2.2 Bronchoscopy Visit
(paragraph 1)	A bronchoscopy and attendant procedures (spirometry and bronchodilation) will be performed during this visit. Subjects should not have experienced any acute illness in the 2 weeks prior to study visit or the visit should be postponed until the subject can meet this prerequisite. If an asthmatic subject cannot complete the bronchoscopy visit within 28 days of the baseline visit, they can be considered to be part of the non-bronchoscopy cohort. If the non-bronchoscopy cohort been completely enrolled, then the subject will be discontinued from the study. Healthy subjects that cannot complete the bronchoscopy visit within 28 days of the baseline visit will be discontinued from the study.	A bronchoscopy and attendant procedures will be performed during this visit **and should occur within 5 to 14 days after the baseline visit**. Details of the bronchoscopy procedure are described in the Study Reference Manual.
(paragraph 2)	None. New text insert.	**Healthy subjects should meet the following criteria or the bronchoscopy visit should be postponed until the subject can meet these prerequisites.**<br>• **No intercurrent illness in the 14 days prior to the bronchoscopy including upper respiratory tract infections etc.**<br>• **Post-bronchodilator $FEV_1 \geq 80\%$ predicted that has not declined by more than 10% from screening. For example, if baseline FEV_1 is 3.86 liters, a decrease of ≥ 386 mL would render the subject ineligible for the bronchoscopy on that day.**<br>• **Healthy subjects who cannot complete the bronchoscopy visit within 14 days of the baseline visit will be given additional 14 days to complete the procedure. If the subject could not complete the bronchoscopy after additional 14 days they will be discontinued from the study.**

(paragraph 3)		**Asthmatic subjects should not withhold any inhaled medications before the bronchoscopy visit. A pre- and post-bronchodilator reversibility test will be performed prior to the bronchoscopy. The postbronchodilator FEV_1 from this test serves as the prebronchoscopy FEV_1 baseline determining eligibility. Asthmatic subjects must meet the following criteria or the bronchoscopy visit should be postponed until the subject can meet these prerequisites.**<br>• **Must be between 18 and 55 years of age, inclusive, at informed consent.**<br>• **Clinically stable asthma for at least 14 days prior to the bronchoscopy procedure, as defined by:**<br>- **no clinically significant worsening of asthma requiring augmentation of therapy (eg, increased use of rescue medications, antibiotics, systemic steroids, increased controller medications, hospitalization) as per judgment of the investigator,**<br>• **Post-bronchodilator $FEV_1 \geq 60\%$ predicted that has not declined by more than 10% from screening. For example, if baseline FEV_1 is 2.56 liters, a decrease of ≥256 mL would render the subject ineligible for the bronchoscopy on that day.**<br>• **No intercurrent illness in the 14 days prior to the bronchoscopy including upper respiratory tract infections etc.**
(paragraph 4)	If an asthmatic subject cannot complete the bronchoscopy visit within 28 days of the baseline visit, they can be considered to be part of the non-bronchoscopy cohort. If the non-bronchoscopy cohort been completely enrolled, then the subject will be discontinued from the study. Healthy subjects that cannot complete the bronchoscopy visit within 28 days of the baseline visit will be discontinued from the study.	If an asthmatic subject cannot complete the bronchoscopy visit within **14** days of the baseline visit, **that subject** can be considered to be part of the non-bronchoscopy cohort. If the non-bronchoscopy cohort **has already** been completely enrolled, then the subject will be discontinued from the study.

(paragraph 5)	Subjects receiving a bronchoscopy may be required to remain overnight in the study unit for follow-up if their bronchodilator response or FEV1 values after bronchoscopy do not return within 4 hours to ≥ 80% of the values obtained prior to bronchoscopy or, in the opinion of the Investigator, a subject should remain overnight for observation. Subjects should be treated with regular bronchodilators with repeat spirometry in the morning and undergo reassessment before discharge. Subjects will be discharged when their FEV1 values return to ≥ 80% and based on the Investigator's assessment of the subject's well being.	Subjects receiving a bronchoscopy **will** be required to remain overnight in the study unit **or a suitable medical facility** for follow-up if, in the opinion of the investigator**, they are unfit for discharge based on symptoms, physical examination, vital signs, oximetry, and lung function parameters. In such a case,** subjects should be treated with regular bronchodilators **and any other treatments that may be medically indicated according to the judgment of the investigator** and undergo reassessment, **including spirometry,** before discharge **the next day.** Subjects will **then** be discharged based on the assessment of the **PI**.
(paragraph 6)	Blood and urine samples for biomarker analysis will also be collected from asthmatic subjects during this visit.	Blood and urine samples for biomarker analysis will also be collected from **all** subjects **undergoing bronchoscopy** during this visit.
7.2.3 Biomarker Visits (Asthmatic Subjects) (heading)	7.2.3Biomarker Visits (3, 6 and 12 Months After Baseline Visit) Asthmatic Subjects	7.2.3Biomarker Visits (Asthmatic Subjects)
(paragraph 1)	Additional visits are scheduled at 3, 6 and 12 months after the initial baseline visit for asthmatic subjects to monitor disease activity. Subjects should not have experienced any acute illness in the 2 weeks prior to study visit or the visit should be postponed until the subject can meet this prerequisite. Each visit will include completion of ACQ and AQLQ, review of subject asthma event logs, lung function testing and collection of blood and urine for biomarker analysis. The 6 month visit will also include the collection of induced sputum samples.	Additional visits are scheduled at 3, 6 and 12 months after the initial baseline visit for asthmatic subjects to monitor disease activity **and should occur within ± 14 days of that time point. All biomarker visit procedures must be completed within a 7 day period.** Subjects should not have **had** any acute illness in the 2 weeks prior to study visit or the visit should be postponed until the subject can meet this prerequisite. Each visit will include completion of ACQ and AQLQ, review of subject asthma event logs, lung function testing that includes **bronchodilator reversibility,** and collection of blood and urine for biomarker analysis. The 6-month visit will also include the collection of **an** induced sputum sample. **If the sputum sample is not adequate, one additional attempt to obtain sputum should be scheduled at least 48 hrs after the first attempt.**

(paragraph 2)	None. New paragraph inserted before original paragraph 2.	**If a subject is not available for a particular biomarker visit the investigator should contact the sponsor medical monitor and discuss options for completing/rescheduling that visit.**
7.3.3 Bronchoscopy (Endobronchial Biopsies and Brushings) (paragraph 2)	None	**After the procedures, subjects will receive a short acting bronchodilator and spirometry will be performed. Discharge of subjects will be based on the principal investigator's clinical judgment taking the spirometry readings into account. Additional short-acting bronchodilators will be administered if required. All medication administered during the bronchoscopy procedure will be captured in the eCRF concomitant medication log. If the PI determines that a subject is not fit for discharge at 4 hours after the procedure, that subject will be kept overnight in a medical facility and be reassessed for discharge in the morning.**
7.3.4 Induced Sputum (paragraph 1, sentence 2)	All subjects will be required to have a post-bronchodilator FEV1 of ≥ 60% predicted on the day of induction for induction with 3% hypertonic saline.	All subjects will be required to have a post-bronchodilator FEV_1 of ≥ 60% predicted on the day of induction for induction with 3%**, 4% and 5%** hypertonic saline.
(paragraph 1, sentence 3)	If post-bronchodilator FEV_1 is from ≥ 50% to < 60%predicted on the day of the induced sputum visit, induction will be performed with normal saline.	If post-bronchodilator FEV_1 is from ≥ 50% to < 60% predicted on the day of the induced sputum visit, induction will be performed with normal **(isotonic) 0.9%** saline.
(paragraph 1, sentence 4)	If post-bronchodilator FEV_1 is < 50%, no sputum induction will be performed.	If post-bronchodilator FEV_1 is < 50%, no sputum induction will be performed **on that day and the visit should be rescheduled.**
(paragraph 1, sentence 5)	None.	**An acceptable sputum sample requires a selected plug weight of at least 50mg and < 20% squamous cells.**

(paragraph 2)	None. New paragraph 2 inserted ahead of original paragraph 2.	**After the procedures, subjects will receive a short acting bronchodilator and spirometry will be performed. All medication administered during the bronchoscopy procedure will be captured in the eCRF concomitant medication log. Discharge of subjects will be based on the principal investigator's clinical judgment taking the spirometry readings into account. Additional short-acting bronchodilators will be administered if required. If the PI determines that a subject is not fit for discharge at 4 hours after the procedure, that subject will be kept overnight in a medical facility and be reassessed for discharge in the morning.**
7.4 Spirometry and Bronchodilator Response (paragraph 1, sentence 3)	None.	Confirmation of the training, as well as any updates, must be maintained at the study site.
(paragraph 3, sentence 2)	In addition, post bronchodilator PFTs are performed 15 to 30 (± 5) minutes after administration of 4 puffs of albuterol/salbutamol via metered dose inhaler (MDI) with a spacer (albuterol/salbutamol reversibility) and % predicted FEV_1 will be calculated using previously established formulas.	In addition, post bronchodilator PFTs are performed 15 to 30 (± 5) minutes after administration of albuterol/salbutamol via metered dose inhaler (MDI) with a spacer **or treatment with nebulized albuterol/salbutamol** (albuterol/salbutamol reversibility) and % predicted FEV_1 will be calculated using previously established formulas **(see Study Reference Manual).**
(paragraph 3, sentence 3)	None	**All medication administered during the bronchoscopy procedure will be captured in the eCRF concomitant medication log.**
(paragraph 4)	A detailed description of the evaluation of PFTs and formulas used to calculate reversibility and percent predicted FEV_1 are described in Study Reference Manual.	A detailed description of **the lung function testing appears in the** Study Reference Manual.

7.5 Methacholine Challenge (paragraph 1)	None.	**The methacholine challenge will not be performed if a subject has a pre-bronchodilator FEV_1 ≤ 60% of predicted on the day of the test. Detailed instructions for conducting methacholine challenge test are described in Study Reference Manual.**
(paragraph 2, sentence 1)	A methacholine challenge test will be performed at screening in asthma subjects if needed in order to assess airway responsiveness.	A methacholine challenge test will be performed at screening in asthma subjects if needed in order to assess **study eligibility**.
(paragraph 3)	In this test baseline spirometry for a subject is measured followed by inhalation of aerosolized saline followed by increasing concentrations of aerosolized methacholine up to a predetermined limit (usually 32 mg/mL). The provocative dose (PC20) is defined a dose of methacholine required to cause a >20% decrease from the baseline post-saline FEV_1 value. A PC20 result of 16 mg/mL will be considered to reflect increase airway responsiveness.	In **the methacholine challenge** test, baseline spirometry for a subject is measured by inhalation of increasing concentrations of aerosolized methacholine up to **16** mg/mL. The provocative dose (PC20) is defined **as the** dose of methacholine required to cause a >20% decrease from the baseline FEV_1 value. A PC20 result of ≤ 16 mg/mL will be considered to reflect increase airway responsiveness **for fulfilling eligibility criteria.**
(paragraph 4)	The methacholine challenge will not be completed if a subject has a pre-bronchodilator FEV_1 ≤ 60% of predicted on the day of the test. Detailed instructions for conducting methacholine challenge test are described in Study Reference Manual.	**After the procedures, subjects will receive a short acting bronchodilator and spirometry will be performed. Discharge of subjects will be based on the principal investigator's clinical judgment taking the spirometry readings into account. Additional short-acting bronchodilators will be administered if required. If the PI determines that a subject is not fit for discharge at 4 hours after the procedure, that subject will be kept overnight in a medical facility and be reassessed for discharge in the morning. All medication administered during the bronchoscopy procedure will be captured in the eCRF concomitant medication log.**

7.6 Fractional Exhaled NO (FENO) (paragraph 1)	Fractional exhaled nitric oxide will be measured at an exhalation flow rate of 50 mL/s according to ATS guidelines using an exhaled NO meter that is approved for asthma measurements. Three replicate measurements will be obtained that agree at the 10% level and up to a total of 8 measurements will be performed to achieve this level of agreement. The procedures for the collection of FENO are described in details in Study Reference Manual.	Fractional exhaled nitric oxide will be measured **for all asthmatic subjects** at an exhalation flow rate of 50 mL/s according to ATS guidelines using an exhaled NO meter that is approved for asthma measurements. **FENO testing must precede any other pulmonary procedures. Two** replicate **FENO** measurements will be obtained that agree at the 10% level and up to a total of 8 measurements will be performed to achieve this level of agreement. The procedures for the collection of FENO are described in detail in Study Reference Manual.
7.8 Asthma Quality of Life Questionnaire (AQLQ) (paragraph 1, sentence 1)	The Asthma Quality of Life Questionnaire is a 32 item, patient-reported outcome questionnaire to evaluate symptoms and several aspects of daily living in adult subjects (age 18+) with asthma (Juniper et al, 1999a; Juniper et al, 1993).	**Asthmatic subjects only will be asked to complete the** Asthma Quality of Life Questionnaire, a 32 item, patient-reported outcome questionnaire to evaluate symptoms and several aspects of daily living in adult subjects (age 18+) with asthma (Juniper et al, 1999a; Juniper et al, 1993).
7.8 Asthma Quality of Life Questionnaire (AQLQ) (paragraph 1, sentence 9)<br>7.9 Asthma Control Questionnaire (ACQ) (paragraph 2, sentence 3)	None.	**The questionnaire will be provided in paper format to the study centers.**
7.9Asthma Control Questionnaire (ACQ) (paragraph 1, sentence 1)	The Asthma Control Questionnaire instrument is designed to evaluate asthma control defined as "the full range of clinical impairment that patients with asthma may experience as a result of the disease" (Juniper et al, 1999b).	**Asthmatic subjects only will be asked to complete** the Asthma Control Questionnaire, **an** instrument designed to evaluate asthma control defined as "the full range of clinical impairment that patients with asthma may experience as a result of the disease" (Juniper et al, 1999b).
7.10 Asthma History Questionnaire (paragraph 1, sentence 2)	The questionnaire will be provided in the Study Reference Manual.	The questionnaire will be provided in **paper format to the study centers.**

7.11 Safety Evaluations (Clinical Laboratory Tests subheading, bullet 1)	• Urine Pregnancy Testing for women of childbearing potential only.	• Urine Pregnancy Testing for **all** women **participating in the study**.
(Vital Signs subheading, paragraph 1, sentence 1)	Measurement of supine vital signs (resting pulse rate, blood pressure, and temperature) will be performed after 5 minutes of rest at the timepoints specified in the study schedule of events (see Attachment 1).	Measurement of supine vital signs (resting pulse rate, blood pressure, and temperature) will be performed after **10** minutes of rest at the timepoints specified in the study schedule of events (see Attachment 1).
(Vital Signs subheading, paragraph 3)	None	**A detailed listing of vital signs to be evaluated will be provided in the Study Reference Manual.**
(Physical Examination subheading, paragraph 4)	None	**A detailed listing of body systems to be evaluated for initial physical exam and subsequent brief physical exams will be provided in the Study Reference Manual.**
7.12 Genomic Evaluation (paragraphs 1, 2 and 3)	Original text was from Section 7.3.5 Genomic Evaluation, which was deleted.<br><br>7.3.5 Genomic Evaluation<br>Specific genomic testing may be undertaken in consenting subjects (subjects participating in this portion of the study must sign a separate informed consent). The procedure will involve taking a blood sample that may be analyzed for specific target genes that may play a role in asthma. Any genomic assessments will be performed in strict adherence to current subject confidentiality standards for genetic testing.	7.**12** **Genomic Evaluation**<br><br>**There are 2 parts to the genomic component of this study.**<br><br>**Subjects will be given the option to participate in Part 1 only, Part 2 only, both parts, or neither part of the genomic component of this study (where local regulations permit).**<br><br>**Analysis Related to the Study (Part 1)**<br><br>**Collection of genomic samples will allow for genetic research to help understand asthma. Genetic analysis will be conducted if it is hypothesized that this may help resolve issues with the clinical data. DNA samples will only be used for genetic research related to asthma. Genetic research may consist of the analysis of 1 or more candidate genes or of the analysis of genetic markers throughout the genome (as appropriate) in relation to asthma clinical endpoints. Subjects will be offered the separate option to consent to storage of their optional samples for future research as scientific discoveries are made.**

		**DNA Storage for Future Research (Part 2)**<br>**Part 2 of the genomic research allows for the storage of DNA samples for future genetic research related to asthma. DNA samples will be stored for up to 5 years after the study is complete. Stored DNA samples and relevant clinical data will be de-identified after the Clinical Study Report has been issued. This involves removing personal identifiers and replacing the study subject identifier with a new number to limit the possibility of linking genetic data to a subject's identity.**
8.1 Completion<br>(paragraph 1, sentence 1)	A healthy subject will be considered to have completed the study if he or she has completed the bronchoscopy procedure and telephone follow-up (within one week after bronchoscopy).	A healthy subject will be considered to have completed the study if he or she has completed the bronchoscopy procedure and telephone follow-**ups on the day following bronchoscopy and 1** week after bronchoscopy.
8.2 Withdrawal From the Study<br>(bullet 6)	None	• **The study investigator or Centocor for any reason stops the study or stops the subject's participation in the study**
(paragraph 2)	None	**Subjects who terminate study participation before their last scheduled visit should contact the investigational site if possible for a final visit. The Study coordinator should consult with the Sponsor as to what testing should be performed at that visit.**
10.1.1 Adverse Event Definitions and Classifications<br>(paragraph 1)	An adverse event is any untoward medical occurrence in a clinical study subject administered a medicinal (investigational or non investigational) product or underwent a study procedure. As no therapeutic intervention is involved with this study, only events related to the study procedures, including spirometry, bronchoscopy, induced sputum, methacholine challenge and blood collection will be reported as adverse events.<br>An adverse event can therefore be any unfavorable and unintended sign (including an abnormal finding), symptom, or disease temporally associated with a study procedure.	An adverse event is any untoward medical occurrence in a clinical study subject administered a medicinal (investigational or non investigational) product or underwent a study procedure. **An adverse event does not necessarily have a causal relationship with the treatment.** An adverse event can therefore be any unfavorable and unintended sign (including an abnormal finding), symptom, or disease temporally associated with **the use of a medicinal (investigational or non investigational) product or study procedure, whether or not related to that medicinal (investigational or non investigational) product or study procedure. (Definition per International Conference on Harmonisation [ICH])**

(paragraph 2)	None	**As no therapeutic intervention is involved with this study, only events temporally associated with a study procedure and determined to be possibly, probably or very likely related to a study procedure including spirometry, bronchoscopy, induced sputum, methacholine challenge and blood collection will be reported as adverse events.**
10.2.2 Serious Adverse Events (paragraph 3, bullet 4)	• The event can be attributed to agents other than the study drug or to factors unrelated to study conduct	• The event can be attributed to **factors** other than study **procedures** or study conduct
11.1 Study-Specific Design Considerations (paragraph 2, sentence 7)	The overall risk of sputum induction is extremely low and there are no reported cases of death.	The overall risk of sputum induction is low and there are no reported cases of death **(see Study Reference Manual for safety monitoring)**.
(paragraph 4, sentence 1)	The bronchsocopy procedure includes insertion of the scope via the nares or oral cavity and hence through the vocal cords into the lower respiratory tree.	The **bronchoscopy** procedure includes insertion of the scope via **a naris** or **the** oral cavity and hence through the vocal cords into the lower respiratory tree.
11.2.2 Independent Ethics Committee or Institutional Review Board (IEC/IRB) (paragraph 4, bullet 6)	None	• **Reports of adverse events that are serious, unlisted/unexpected, and associated with a study procedure**
11.2.5 Long-Term Storage of Samples for Future Research (entire section)	None	**11.2.5 Long-Term Storage of Samples for Future Research**<br>**After publication of the final study report, samples and corresponding relevant clinical data will be made nonidentifiable by the removal of personal identifiers. Samples will be stored up to 5 years after completion of the study report. Only research related to asthma will be done on stored samples.**

12.2.2 Required Prestudy Documentation<br>(paragraph 2, bullet 1)<br>(paragraph 2, sentence 1)	• Completed investigator financial disclosure forms from all subinvestigators<br><br>The following documents must be provided to the sponsor before enrollment of the first subject:	• Completed investigator financial disclosure forms from all **clinical** subinvestigators, **where required**<br><br>The following documents must be provided to the sponsor before **screening** of the first subject:
12.8 Monitoring	The first post-initiation visit will be made as soon as possible after enrollment has begun.	The first post-initiation visit will be made as soon as possible after enrollment has begun (**when the first subject successfully completes the screening period) or in accordance with monitoring guideline, whichever occurs first.**
Attachment 1.1 Study Assessments for Healthy Subjects (new entries)	FENO measurement<br>Spirometry and bronchodilator response	deleted<br>changed to:<br>Spirometry prebronchodilator<br>Spirometry postbronchodilator
(Telephone Follow-up entry)	Scheduled for after bronchoscopy only<br><br>a. Review eligibility criteria and laboratory assessments.<br>b. Includes amphetamines, barbiturates, benzodiazepines, cannabinoids, cocaine, methadone, opiates, tricyclic antidepressants and a screen for urine cotinine.<br>c. See Attachment 2 for list of laboratory tests to be run.<br>d. Phadiatop test done at screening for all cohorts. Phadiatop test will be used for subject exclusion in the healthy cohort only.<br>e. Supine vital signs should be measured after 5 minutes of rest and include temperature, resting pulse rate, and blood pressure. When scheduled concurrently with a blood draw or ECG, vital signs should be measured after the ECG and before the blood draw.<br>f. Pregnancy test will be performed for all female subjects prior to study procedures.<br>g. Healthy subjects will be excluded if bronchodilator response is >12% or prebronchodilator FEV1 values are ≤ 85% of predicted values.	Scheduled for after screening, baseline and bronchoscopy visits.<br><br>**a. Screening can be conducted over a period of 21 days.**<br>**b. The baseline procedures can be conducted over a period of 7 days.**<br>**c.** Review eligibility **and safety** criteria **for the induced sputum and bronchoscopy procedures.**<br>d. Includes amphetamines, barbiturates, benzodiazepines, cannabinoids, cocaine, methadone, opiates, tricyclic antidepressants, a screen for urine cotinine **and alcohol breath test.**<br>**e.** See Attachment 2 for the list of **serologic, serum chemistry, hematologic and urine laboratory tests to be completed.**<br>**f. Blood draw for serology and Phadiatop test can be performed when the blood draw for serum chemistry, hematology and PT/aPTT is being done.**<br>g. Phadiatop test will be used for subject exclusion in the healthy cohort only.

	h. To be done both before and after bronchoscopy<br>i. Healthy subjects may be required to remain overnight in the study unit for follow-up if their bronchodilator response or FEV1 values after bronchoscopy do not return within 4 hours to ≥ 80% of the values obtained prior to bronchoscopy or, in the opinion of the Investigator, a subject should remain overnight for observation. Subjects will be discharged when their FEV1 values return to ≥ 80% and based on the Investigator's assessment of the subject's well being. See Section 7.2.2 for follow-up procedures.<br>j. Requires an additional informed consent from the subject.<br>k. Follow-up telephone call should occur within one week following the bronchoscopy.<br>l. If a subject does not produce sputum at screening, they may be given a second opportunity to do so.	h. Supine vital signs should be measured after **10** minutes of rest and include temperature, resting pulse rate, and blood pressure. When scheduled concurrently with a blood draw or ECG, vital signs should be measured after the ECG and before the blood draw.<br>i. Pregnancy test will be performed **all** for female subjects prior to study procedures.<br>**j. Only adverse events related to study procedures should be recorded.**<br>k. Healthy subjects will be excluded if the pre-bronchodilator **FEV_1 value is < 85% of predicted value, and bronchodilator response is ≥ 12% and 200 mL**.<br>**l. The post bronchodilator FEV_1 value obtained at this visit serves as eligibility criterion for induced sputum if the induced sputum procedure is performed within 60 minutes. This FEV_1 value also serves as the pre-induction FEV_1 baseline and as a reference for post procedure FEV_1 recovery value.**<br>**m. Post bronchodilator spirometry will be performed at this visit if subjects' post procedure FEV_1 does not return to 80% of pre induction FEV_1 baseline. (see Study Reference Manual).**<br>**n. Pre- and postbronchodilator FEV_1 values are assessed before bronchoscopy and the postbronchodilator FEV_1 serves as the prebronchoscopy baseline for eligibility and safety monitoring. After the bronchoscopy, a short-acting bronchodilator may be administered and spirometry is repeated (see Study Reference Manual).**

		**o.** If a subject does not produce a valid sputum sample at screening, they may be given a second opportunity to do so **within the screening window (21 days). Sputum Induction may be repeated at the baseline visit, if required, within the 7 day window for this visit. The second sputum induction should occur no earlier than 48 hours from first attempt for both the screening and baseline visits.**<br>**p.** Subjects **will** be required to remain overnight in the study unit for follow-**up if, based on the clinical judgment of the Investigator, a subject is unfit for discharge. Subjects will undergo reassessment, including spirometry, the following day and will be discharged based on the Investigator's assessment of the subject's well being. See Section 7.2.2 and the Study Reference Manual for follow-up procedures.**<br>q. Requires an additional informed consent from the subject.<br>r. **Telephone contact should be made on the day after sputum induction and both 1 and 7 days after bronchoscopy to collect any procedure related adverse events.**
Attachment 1.2 Study Assessments for Asthmatic Subjects Receiving a Bronchoscopy<br>Attachment 1.3 Study Assessments for Asthmatic Subjects Not Receiving a Bronchoscopy<br>(new entries)	None<br><br>Spirometry and bronchodilator response	Telephone follow-up<br><br>Spirometry prebronchodilator<br>Spirometry postbronchodilator

(footnotes)	a. Review eligibility criteria and laboratory assessments.<br>b. Includes amphetamines, barbiturates, benzodiazepines, cannabinoids, cocaine, methadone, opiates, tricyclic antidepressants and a screen for urine cotinine.<br>c. See Attachment 2 for list of laboratory tests to be run.<br>d. Phadiatop test done at screening for all cohorts. Phadiatop test will be used for subject exclusion in the healthy cohort only.<br>e. Supine vital signs should be measured after 5 minutes of rest and include temperature, resting pulse rate, and blood pressure. When scheduled concurrently with a blood draw or ECG, vital signs should be measured after the ECG and before the blood draw.<br>f. Pregnancy test will be performed for all female subjects.<br>g. Subject diary should be filled out from screening through bronchoscopy and for the 2 weeks prior to each biomarker visit.<br>h. To be done both before and after bronchoscopy<br>i. If prebronchodilator FEV1 is ≤ 60%, PC20 methocholine should not be done.<br>j. PC20 methacholine to be done within 5 days of screening if no bronchodilator response identified and no documented PC20 available.<br>k. PC20 methacholine on the next day for subjects who did not receive this procedure during screening.<br>l. If a subject does not produce sputum at screening, they may be given a second opportunity to do so.	(Footnotes related to bronchoscopy visit are not applicable to Attachment 1.3. Footnotes in Attachment 1.3 may have a different alphabetical sequence as a result.)<br>**a. Screening can be conducted over a period of 21 days.**<br>**b. The baseline and 6-month biomarkers procedures can be conducted over a period of 7 days.**<br>**c. These visits should be conducted ± 14 days of the scheduled time.**<br>**d.** Review eligibility **and safety** criteria **for the induced sputum and bronchoscopy procedures (see Sections 4.3.1 and 7.2.2).**<br>**e.** Includes amphetamines, barbiturates, benzodiazepines, cannabinoids, cocaine, methadone, opiates, tricyclic antidepressants, a screen for urine cotinine **and alcohol breath test.**<br>**f.** See Attachment 2 for the list of **serologic, serum chemistry, hematologic and urine laboratory tests to be completed.**<br>**g. Blood draw for serology and Phadiatop test can be performed when the blood draw for serum chemistry, hematology and PT/aPTT is being done.**<br>h. Phadiatop test is completed at baseline visit as it is not an inclusion criterion for asthmatics.<br>i. Supine vital signs should be measured after **10** minutes of rest and include temperature, resting pulse rate, and blood pressure. When scheduled concurrently with a blood draw or ECG, vital signs should be measured after the ECG and before the blood draw.

	m. Asthmatic subjects may be required to remain overnight in the study unit for follow-up if their bronchodilator response or FEV1 values after bronchoscopy do not return within 4 hours to ≥ 80% of the values obtained prior to bronchoscopy or, in the opinion of the Investigator, a subject should remain overnight for observation. Subjects will be discharged when their FEV1 values return to ≥ 80% and based on the Investigator's assessment of the subject's wellbeing. See Section 7.2.2 for follow-up procedures.<br>n. Requires an additional informed consent from the subject.	**j.** Pregnancy test will be performed for all female subjects **prior to study procedures.**<br>**k. Only adverse events related to study procedures should be recorded.**<br>**l. Asthma diary and medical event log should be filled out continuously from screening through the bronchoscopy visit and for 2 weeks prior to each biomarker visit.**<br>**m. Some asthma medications are withheld prior to reversibility testing – pre and post bronchodilator FEV_1 response. For a detailed list of medications, see Study Reference Manual.**<br>**n.** If prebronchodilator FEV_1 is < 60%, PC20 methacholine should not be done**. PC20 methacholine challenge test cannot be performed on the same day as reversibility testing.**<br>**o. PC20 methacholine is done at screening if FEV_1 bronchodilator response is < 12% and no documented reversibility or PC20 is available (see Sections 4.3.1 and 7.5).**<br>**p. Some asthma medications are withheld prior to PC20 methacholine test. For detailed list of medications, see Study Reference Manual.**<br>**q. PC20 methacholine test is performed at the baseline visit if not conducted at the screening visit.**<br>**r.** If a subject does not produce a valid sputum sample at screening, they may be given a second opportunity to do so **within the screening window (21 days). Sputum induction may also be repeated at the baseline and 6-month biomarker Visit if required within the 7 day window for this visit. The second sputum induction should occur no earlier than 48 hours from first attempt for the screening baseline and biomarker visits.**

		**s. Sputum induction may be performed on the same day as PC20 methacholine test with the PC20 methacholine test being conducted first followed by induced sputum. The postbronchodilator FEV_1 obtained after the PC20 methacholine test can serve as a pre sputum induction baseline value if sputum induction occurs within 60 minutes. This pre induction baseline FEV_1 value serves to determine eligibility for sputum induction, which strength of saline should be used, and for safety monitoring (see Study Reference Manual).**<br>**t.** Asthmatic subjects **will** be required to remain overnight in the study unit for follow-up if, **based on the clinical judgment of the Investigator, a subject is unfit for discharge. Subjects will undergo reassessment, including spirometry, the following day and will be discharged based on the Investigator's assessment of the subject's well being See Section 7.2.2 and the Study Reference Manual for follow-up procedures.**<br>u. Requires an additional informed consent from the subject.<br>**v. The study coordinator should contact the subject by telephone 7 days and 24 hours before the baseline, bronchoscopy and 6 month biomarker visits to ensure that subject's asthma is well controlled before the visit.**<br>**w. Telephone contact should be made the day after the sputum induction procedure to collect any procedure related adverse events.**<br>**x. Telephone contact should be made 1 and 7 days after the bronchoscopy visit to collect any procedure related adverse events.**

ATTACHMENT 2: CENTRAL LABORATORY ASSESSMENTS (header)<br>(footnote e)	ATTACHMENT 2: LOCAL LABORATORY ASSESSMENTS<br><br>None	ATTACHMENT 2: **CENTRAL** LABORATORY ASSESSMENTS<br><br>**e. Test to be conducted at the study center.**
APPENDIX A (heading)	APPENDIX A NHLBI ASTHMA CLASSIFICATION BY SEVERITY AND TREATMENT LEVELS	APPENDIX A
Table A.1 NHLBI Asthma classification by severity and treatment Levels	None. Table is unchanged	**Table A.1 NHLBI Asthma classification by severity and treatment Levels**
Table A.2 Asthma Severity Classification for Study Enrollment	None	**Table A.2 Asthma Severity Classification for Study Enrollment**<br><br>In addition, a new table was added that is to be used to classify asthmatic enrollment into the study.

AMENDMENT 2 - 22 APR 2011

The protocol has been revised to incorporate this amendment, which applies to all study centers. The rationale and description of the changes to the protocol are provided below. Minor formatting and typographical errors have also been corrected to improve the clarity and accuracy of the protocol text; these changes do not affect the overall conduct of the study.

This amendment is considered substantial based on the criteria set forth in article 10(a) of Directive 2001/20EC of the European Parliament and the Council of the European Union.

1. Statements that were listed as bullets were changed to numbers for clarity and ease of reference in the document

The inclusion and exclusion criteria for healthy and asthmatic subjects are listed as bullets in the protocol. The bullets were changed to numbers for better clarity to find a specific criterion.

Sections Affected	Original Content	Amended/New Content
4.2.1 Inclusion Criteria<br><br>4.2.2 Exclusion Criteria<br><br>4.3.1 Inclusion Criteria for Asthmatic Subjects<br><br>4.3.2 Exclusion Criteria for Asthmatic Subjects	Bullets	Numbers

2. Text was clarified on the number of subjects planned for each treatment group

The text was clarified to indicate that an equal number of subjects in each age group are not required only planned as this number is dependent on the number of subjects in each age group.

Sections Affected	Original Content	Amended/New Content
4.1 General Considerations (Paragraph 1)	Equal numbers of healthy subjects will be enrolled into 2 age groups (18 to 36 years of age or 37 to 55 years of age).	Equal numbers of healthy subjects **are planned to** be enrolled into 2 age groups (18 to 36 years of age or 37 to 55 years of age).
5 Study Subject Allocation (Sentence 3)	Subjects will be assigned to 1 of 4 cohorts based on their current asthma status (healthy, mild, moderate, or severe asthma) according to the criteria described in Section 4.1 and Table A.2. All healthy subjects and approximately 30 of the 50 subjects in each of the asthma severity classifications will undergo a bronchoscopy procedure with endobronchial biopsies and bronchial brushings. The healthy subject group will be split equally into 2 age ranges of 18 to 36 years of age or 37 to 55 years of age. Asthma subjects undergoing bronchoscopy must be between 18 and 55 years of age, inclusive.	Subjects will be assigned to 1 of 4 cohorts based on their current asthma status (healthy, mild, moderate, or severe asthma) according to the criteria described in Section 4.1 and Table A.2. All healthy subjects and approximately 30 of the 50 subjects in each of the asthma severity classifications will undergo a bronchoscopy procedure with endobronchial biopsies and bronchial brushings. The healthy subject group will be split into 2 age ranges of 18 to 36 years of age or 37 to 55 years of age. Asthma subjects undergoing bronchoscopy must be between 18 and 55 years of age, inclusive.

3. Reference to Step 2 was added in Section 4.1

There was an oversight in the original protocol and amendment 1 to include Step 2 in the list of references cited.

Sections Affected	Original Content	Amended/New Content
4.1 General Considerations (Bullet 2)	– Low to medium dose inhaled corticosteroid (ICS) alone, or low to medium dose ICS in combination with any other controller medication (eg LABA, leukotriene antagonist (LTRA), theophylline, etc,) with the exception of oral corticosteroids (OCS) and omalizumab (NHLBI STEP 3 or 4; Appendix A).	– Low to medium dose inhaled corticosteroid (ICS) alone, or low to medium dose ICS in combination with any other controller medication (eg LABA, leukotriene antagonist (LTRA), theophylline, etc,) with the exception of oral corticosteroids (OCS) and omalizumab (NHLBI STEP **2,** 3**,** or 4; Appendix A).

4. Expand the BMI inclusion criteria from ≤ 30 kg/m^2 to ≤ 32 kg/m^2

The BMI inclusion criterion was expanded to allow more subjects into the study per the feedback from the principle investigators, and the analysis of the screen failure.

Sections Affected	Original Content	Amended/New Content
4.2.1 Inclusion Criteria (Bullet 5)	• Have a BMI ≤ 30 kg/m^2.	5. Have a BMI ≤ **32 kg/m^2**.
4.3.1 Inclusion Criteria for Asthmatic Subjects (Bullet 5)	• Have a BMI ≤ 30 kg/m^2.	5. Have a BMI ≤ **32 kg/m^2.**

5. NSAID use was also added to inclusion criterion #13 to clarify the time to abstain from these drugs prior to the bronchoscopy procedure

NSAID use can also cause bleeding. The inclusion criterion was updated to allow subjects who are on NSAIDs to abstain from taking any 3 days prior to the bronchoscopy procedure.

Sections Affected	Original Content	Amended/New Content
4.2.1 Inclusion Criteria (Bullet 13)	• No bleeding disorder including use of anticoagulants and antiplatelet agents that could place the subjects at risk for bleeding. Able to abstain from aspirin use for 7 days prior to bronchoscopy without risk.	13. No bleeding disorder including use of anticoagulants and antiplatelet agents that could place the subjects at risk for bleeding. Able to abstain from aspirin use for 7 days **and NSAID use for 3 days** prior to bronchoscopy without risk.
Abbreviations		**NSAID: Nonsteroidal anti-inflammatory drug**

6. Text was clarified for exclusion criterion #4 in Section 4.2.2 and Attachment 1.1

The text was clarified to state "at least" 200 mL from baseline to further clarify that values greater than 200 would exclude a subject from the study.

Sections Affected	Original Content	Amended/New Content
4.2.2 Exclusion Criteria (Bullet 4)	• Have a bronchodilator response of ≥12% and 200 mL from baseline or an FEV_1 value ≤ 85% of predicted value at screening.	4. Have a bronchodilator response of ≥12% and **at least** 200 mL from baseline or an FEV_1 value ≤ 85% of predicted value at screening.
Attachment 1.1 Study Assessments for Healthy Subjects (footnote l)	Healthy subjects will be excluded if the pre-bronchodilator FEV_1 value is < 85% of predicted value, and bronchodilator response is ≥ 12% and 200 mL.	Healthy subjects will be excluded if the pre-bronchodilator FEV_1 value is < 85% of predicted value, and bronchodilator response is ≥ 12% and **at least** 200 mL.

7. A repeat reversibility test will be given to subjects with a reversibility result that is < 12% at screening

Subjects were excluded from the study if the bronchodilator reversibility was < 12% at screening. Text was added to allow subjects a repeat test at the discretion of the principle investigator.

Sections Affected	Original Content	Amended/New Content
4.3.1 Inclusion Criteria for Asthmatic Subjects (Bullet 9; sub-bullet 1)	• Symptoms compatible with asthma for at least 6 months prior to screening (wheezing, dyspnea, chest tightness), PI confirmation of diagnosis of asthma of any severity and exclusion of alternative diagnoses, and at least 1 one the following, tested sequentially at screening or based on historical documentation:<br>– Bronchodilator reversibility after inhalation of SABA at screening or documented within the previous 24 months defined as an increase in FEV_1 ≥ 12% and at least 200 mL from baseline.	9. Symptoms compatible with asthma for at least 6 months prior to screening (wheezing, dyspnea, chest tightness), PI confirmation of diagnosis of asthma of any severity and exclusion of alternative diagnoses, and at least 1 one the following, tested sequentially at screening or based on historical documentation:<br>– Bronchodilator reversibility after inhalation of SABA at screening or documented within the previous 24 months defined as an increase in FEV_1 ≥ 12% and at least 200 mL from baseline. **If reversibility is < 12% and per the opinion of the PI, a repeat bronchodilator reversibility test is performed; this result may be used for subject enrollment.**

8. Exclusion criterion #10 that addressed exclusion of subjects who tested positive for substances of abuse was modified and renumbered
The exclusion criterion for subjects who test positive on the urine toxicology screen was split into 2 criteria to clarify the content for substances that are not acceptable to take and substances that may be acceptable if there is a medical need, and the sponsor's medical monitor agrees to allow the subject into the study.

Sections Affected	Original Content	Amended/New Content
4.3.2 Exclusion Criteria for Asthmatic Subjects (Bullet 10)	• Positive urine toxicology screen for substances of abuse, including but not limited to amphetamines, barbiturates, benzodiazepines, cannabinoids, cocaine, methadone, opiates and tricyclic antidepressants.	**10. Positive urine toxicology screen for substances of abuse, including but not limited to, cannabinoids, cocaine, and methadone.**<br><br>**11.** Positive urine toxicology screen, including but not limited to amphetamines, barbiturates, benzodiazepines, opiates and tricyclic antidepressants **unless the results can be reliably attributed to a concomitant prescription medication by the PI for a condition identified on medical history, in which case the subject may be enrolled after consultation with and agreement of the Sponsor's medical monitor.**

9. Exclusion criterion #5 was added for clarification
A new exclusion criterion was added to exclude subjects who have a PC20 methacholine test > 16 mg/mL for clarity since asthmatic subjects with a PC20 methacholine result ≤ 16 mg/mL are allowed into the study.

Sections Affected	Original Content	Amended/New Content
4.3.2 Exclusion Criteria for Asthmatic Subjects		**5. If a PC20 methacholine test is performed during screening for enrollment, a PC20 result > 16 mg/mL excludes the subject from the study (refer to 4.3.1, inclusion criterion #9).**
4.3.1 Inclusion Criteria for Asthmatic Subjects (Bullet 9; sub-bullet 2)	• Symptoms compatible with asthma for at least 6 months prior to screening (wheezing, dyspnea, chest tightness), PI confirmation of diagnosis of asthma of any severity and exclusion of alternative diagnoses, and at least 1 one the following, tested sequentially at screening or based on historical documentation:	9. Symptoms compatible with asthma for at least 6 months prior to screening (wheezing, dyspnea, chest tightness), PI confirmation of diagnosis of asthma of any severity and exclusion of alternative diagnoses, and at least 1 one the following, tested sequentially at screening or based on historical documentation:

	– Bronchodilator reversibility after inhalation of SABA at screening or documented within the previous 24 months defined as an increase in $FEV_1 \geq 12\%$ and at least 200 mL from baseline.<br>– PC20 methacholine ≤ 16 mg/mL at screening or documented within 24 months prior to screening.	– Bronchodilator reversibility after inhalation of SABA at screening or documented within the previous 24 months defined as an increase in $FEV_1 \geq 12\%$ and at least 200 mL from baseline.<br>– PC20 methacholine ≤ 16 mg/mL at screening or documented within 24 months prior to screening. **If the PC20 is > 16 mg/mL, the subject is ineligible.**

10. The order for the study evaluations was switched with the 12-lead ECG listed before the Vital signs evaluation
When the vital signs are scheduled concurrently with a blood draw or ECG, vital signs should be measured after the ECG evaluation and before the blood draw.

Sections Affected	Original Content	Amended/New Content
7 Study Evaluations (Paragraph 3)	Procedures will be performed at the times and order indicated in study procedures section of the Schedule of Events (Attachment 1). Asthma subjects should complete the ACQ and AQLQ questionnaires prior to completing any study procedures. When multiple procedures are performed at a given visit, the following guideline for order of procedures is recommended:<br>• ACQ<br>• AQLQ<br>• Physical Exam<br>• Vital signs<br>• 12-lead ECG	Procedures will be performed at the times and order indicated in study procedures section of the Schedule of Events (Attachment 1). Asthma subjects should complete the ACQ and AQLQ questionnaires prior to completing any study procedures. When multiple procedures are performed at a given visit, the following guideline for order of procedures is recommended:<br>• ACQ<br>• AQLQ<br>• Physical Exam<br>• **12-lead ECG**<br>• **Vital signs**

11. The time allotted for bronchoscopy visit was expanded from 14 to 28 days
Healthy subjects who were not able to complete the bronchoscopy visit by Day 14 were being discontinued from the study. By expanding the timeframe, this will give the opportunity for subjects to stay in the study.

Sections Affected	Original Content	Amended/New Content
7.2.2 Bronchoscopy Visit (Paragraph 4)	If an asthmatic subject cannot complete the bronchoscopy visit within 14 days of the baseline visit, that subject can be considered to be part of the non-bronchoscopy cohort. If the non-bronchoscopy cohort has already been completely enrolled, then the subject will be discontinued from the study.	**Asthmatic subjects who cannot complete the bronchoscopy visit within 14 days of the baseline visit will be given an additional 14 days to complete the procedure.** If an asthmatic subject cannot complete the bronchoscopy visit within **28** days of the baseline visit, that subject can be considered to be part of the non-bronchoscopy cohort. If the non-bronchoscopy cohort has already been completely enrolled, then the subject will be discontinued from the study.
Attachment 1.1 Study Assessments for Healthy Subjects (New footnote c; subsequent footnotes were re-lettered)	Bronchoscopy 5-14 days after baseline	Bronchoscopy[c] 5-14 days after baseline<br><br>**c. Subjects who cannot complete the bronchoscopy visit within 14 days of the baseline visit will be given an additional 14 days to complete the procedure. If the subject could not complete the bronchoscopy after the additional 14 days they will be discontinued from the study.**
Attachment 1.2 Study Assessments for Asthmatic subjects Receiving a Bronchoscopy (New footnote c; subsequent footnotes were re-lettered)	Bronchoscopy 5-14 days after baseline	Bronchoscopy 5-14 days after baseline[c]<br><br>**c. Asthmatic subjects who cannot complete the bronchoscopy visit within 14 days of the baseline visit will be given an additional 14 days to complete the procedure. If an asthmatic subject cannot complete the bronchoscopy visit within 28 days of the baseline visit, that subject can be considered to be part of the non-bronchoscopy cohort. If the non-bronchoscopy cohort has already been completely enrolled, then the subject will be discontinued from the study.**

12. The provocative dose definition for the methocholine challenge required a correction

The provocative dose (PC20) is defined as the dose of methocholine required to cause a ≥ 20% decrease from the baseline FEV_1 value. The ≥ 20% decrease from baseline was incorrectly stated as the >20% in the original protocol and is being corrected in this amendment.

Sections Affected	Original Content	Amended/New Content
7.5 Methocholine Challenge (Paragraph 3, Sentence 2)	In the methacholine challenge test, baseline spirometry for a subject is measured by inhalation of increasing concentrations of aerosolized methacholine up to 16 mg/mL. The provocative dose (PC20) is defined as the dose of methacholine required to cause a > 20% decrease from the baseline FEV_1 value. A PC20 result of ≤ 16 mg/mL will be considered to reflect increase airway responsiveness for fulfilling eligibility criteria.	In the methacholine challenge test, baseline spirometry for a subject is measured by inhalation of increasing concentrations of aerosolized methacholine up to 16 mg/mL. The provocative dose (PC20) is defined as the dose of methacholine required to cause a **≥ 20%** decrease from the baseline FEV_1 value. A PC20 result of ≤ 16 mg/mL will be considered to reflect increase airway responsiveness for fulfilling eligibility criteria.

13. Text has been revised to remove reference to investigational study agent

The sentence as originally stated was template text that was not applicable to this study as no investigational study agent is being administered to subjects.

Sections Affected	Original Content	Amended/New Content
11.2.4 Privacy of Personal Data (Paragraph 1)	The collection and processing of personal data from subjects enrolled in this study will be limited to those data that are necessary to investigate the efficacy, safety, quality, and utility of the investigational study drug(s) used in this study.	The collection and processing of personal data from subjects enrolled in this study will be limited to those data that are necessary **to fulfill the objectives of the study.**

14. Title was added to the Appendix A title page

No title was with the appendix A page. This was an oversight and a title has been added.

Sections Affected	Original Content	Amended/New Content
Appendix A	Appendix A	Appendix A<br>**NHLBI ASTHMA CLASSIFICATION SYSTEM**

15. Inclusion of additional pregnancy test

The pregnancy urine test was not checked for the 3-month visit on the study assessment table. This was an oversight as a pregnancy test is required at this visit for asthmatic subjects.

Sections Affected	Original Content	Amended/New Content
Attachment 1.3 Study Assessments for Asthmatic Subjects <u>Not</u> Receiving a Bronchoscopy (Urine pregnancy test[j])	Visits: Screen; Baseline[b] 5-14 days after screening; Biomarkers[b] 6 months[c] after baseline	Visits: Screen; Baseline[b] 5-14 days after screening; **Biomarkers 3 months[c] after baseline**; Biomarkers[b] 6 months[c] after baseline

AMENDMENT 3 - 29 MAY 2012

The protocol has been revised to incorporate this amendment, which applies to all study centers. The rationale and description of the changes to the protocol are provided below. Minor formatting and typographical errors have also been corrected to improve the clarity and accuracy of the protocol text; these changes do not affect the overall conduct of the study.

1. The name of the sponsor for this protocol has been changed to Janssen Research & Development. This change is necessary due to a change in the corporate identity of the sponsor. Centocor Research & Development, Inc. now operates under the new sponsor identity of Janssen Research & Development and the legal identity of Janssen Research and Development, L.L.C.

Sections Affected	Original Content	Amended/New Content
Cover Page<br>(Sponsor Name)	Centocor Research & Development, Inc*	**Janssen** Research & Development*
Sponsorship Statement	*Centocor Research & Development, Inc. is a global organization that operates through different legal entities in various countries. Therefore, the legal entity acting as the sponsor for Centocor Research & Development, Inc. studies may vary, such as, but not limited to, Centocor Ortho Biotech Inc.; Centocor, B.V.; Centocor Ortho Biotech Products, L.P.; Janssen-Cilag International NV; or Janssen-Ortho Inc. The term “sponsor” is used throughout the document to represent these various legal entities.	***Janssen Research & Development** is a global organization that operates through different legal entities in various countries. Therefore, the legal entity acting as the sponsor **for Janssen Research & Development studies may vary, such as, but not limited to Janssen Biotech, Inc.; Janssen Products, LP; Janssen Biologics, BV;** Janssen-Cilag International NV; **Janssen, Inc; Janssen Infectious Diseases BVBA; Janssen R&D Ireland; or Janssen Research & Development, LLC.** The term “sponsor” is used throughout the protocol to represent these various legal entities**; the sponsor is identified on the Contact Information page that accompanies the protocol.**
8.2 Withdrawal From the Study<br>(bullet 6)	• The study investigator or Centocor for any reason stops the study or stops the subject’s participation in the study.	• The study investigator or **Sponsor** for any reason stops the study or stops the subject’s participation in the study.

2. An interim database lock has been added to the study. This database lock has been added to facilitate initial biomarker analyses as the bronchoscopy samples are collected early in the study.

Sections Affected	Original Content	Amended/New Content
3 Overview of Study Design (Paragraph 3)	None.	**Two database locks are planned: an interim database lock after completion of the last bronchoscopy procedures for the last subject receiving a bronchoscopy; a second database lock will occur at the end of the study after the last scheduled visit has occurred for the last subject in the study.**
9.3 Interim Analyses (Heading) (Paragraph 1, sentences 1 through 3)	9.3 Interim Analysis<br><br>An interim analysis on bronchoscopy samples will be performed after the first 15 healthy subjects and the first 15 asthma subjects of at least one severity group have undergone bronchoscopy to determine whether to continue with additional bronchoscopy sampling. No formal data base lock will be needed.	9.3 Interim Analyses<br><br>**Two interim analyses are planned for this study. The first interim analysis will be conducted** on bronchoscopy samples **and** will be performed after the first 15 healthy subjects and the first 15 asthma subjects of at least one severity group have undergone bronchoscopy to determine whether to continue with additional bronchoscopy sampling. No formal database lock **is required for this interim analysis.**
(Paragraph 2)	None.	**The second interim analysis will be conducted upon completion of last bronchoscopy procedures in the last subject participating in the bronchoscopy portion of the study. The main goal of this interim analysis will be biomarker evaluation of the samples collected at the screening, baseline, and bronchoscopy visits and will include but will not be limited to microarray analysis of RNA isolated from samples and soluble analyte analysis of serum and IS samples. To support this analysis a data base lock will be performed to provide demographic and clinical information for the planned biomarker analyses. The output of this interim analysis will include, but not be limited to, study population results, safety results, information on asthma characteristics of mild, moderate and severe cohorts**

		**and their respective clinical parameters, differences in biomarkers across cohorts, identification of biomarker-defined subsets across and within cohorts, correlations of biomarker, clinical, and demographic parameters, and correlations of biomarker measurements across sampling matrices.**

3. Changes to correct minor errors were made in the protocol and are listed below.

Sections Affected	Original Content	Amended/New Content
4.1 General Considerations (Paragraph 1, sentence 3)	Equal numbers of healthy subjects are planned to be enrolled into 2 age groups (18 to 36 years of age or 37 to 55 years of age).	**Approximately** equal numbers of healthy subjects are planned to be enrolled into 2 age groups (18 to 36 years of age or 37 to 55 years of age).
7.3.4 Induced Sputum (Paragraph 2, sentence 2)	All medication administered during the bronchoscopy procedure will be captured in the eCRF concomitant medication log.	All medication administered during the **induced sputum** procedure will be captured in the eCRF concomitant medication log.
7.5 Methacholine Challenge (Paragraph 1, sentence 1)	The methacholine challenge will not be performed if a subject has a pre-bronchodilator FEV1 ≤ 60% of predicted on the day of the test.	The methacholine challenge will not be performed if a subject has a pre-bronchodilator FEV1 < 60% of predicted on the day of the test.
Attachment 1.2 Study Assessments for Asthmatic Subjects Receiving a Bronchoscopy<br>Eligibility criteria (entry for Biomarkers 6 months after baseline)<br>Telephone follow-up (entry for Bronchoscopy 5 - 14 days after baseline)	 <br>X[d]<br>X[v,z]	 <br>X[**e**]<br>X[**w,y**]

APPENDIX A: NHLBI ASTHMA CLASSIFICATION SYSTEM

Table A.1 NHLBI Asthma classification by severity and treatment Levels

Components of Severity		Classification of Asthma Severity (Youths ≥12 years of age and adults)			
		Intermittent	Persistent		
			Mild	Moderate	Severe
Impairment Normal FEV_1/FVC: 8-19 yr 85% 20-39 yr 80% 40-59 yr 75% 60-80 yr 70%	Symptoms	≤2 days/week	>2 days/week but not daily	Daily	Throughout the day
	Nighttime awakenings	≤2x/month	3-4x/month	>1x/week but not nightly	Often 7x/week
	Short-acting $beta_2$-agonist use for symptom control (not prevention of EIB)	≤2 days/week	>2 days/week but not >1x/day	Daily	Several times per day
	Interference with normal activity	None	Minor limitation	Some limitation	Extremely limited
	Lung function	• Normal FEV_1 between exacerbations<br>• FEV_1 ≥80% predicted<br>• FEV_1/FVC normal	• FEV_1 ≥80% predicted<br>• FEV_1/FVC normal	• FEV_1 >60% but <80% predicted<br>• FEV_1/FVC reduced 5%	• FEV_1 <60% predicted<br>• FEV_1/FVC reduced >5%
Risk	Exacerbations requiring oral systemic corticosteroids	0-1/year	≥2/year →		
		← Consider severity and interval since last exacerbation. Frequency and severity may fluctuate over time for patients in any severity category. →			
		Relative annual risk of exacerbations may be related to FEV_1			

Lowest level of treatment required to maintain control	Classification of Asthma Severity			
	Intermittent	Persistent		
		Mild	Moderate	Severe
	Step 1	Step 2	Step 3 or 4	Step 5 or 6

Source: http://www.rtmagazine.com/issues/articles/2009-05_01.asp. *Accessed 31 Mar 2010*. Adapted from: National Institutes of Health. Clinical Practice Guidelines: Expert Panel Report 3: Guidelines for the Diagnosis and Management of Asthma. NHLBI: *NIH publication 08-4051*, 2007.

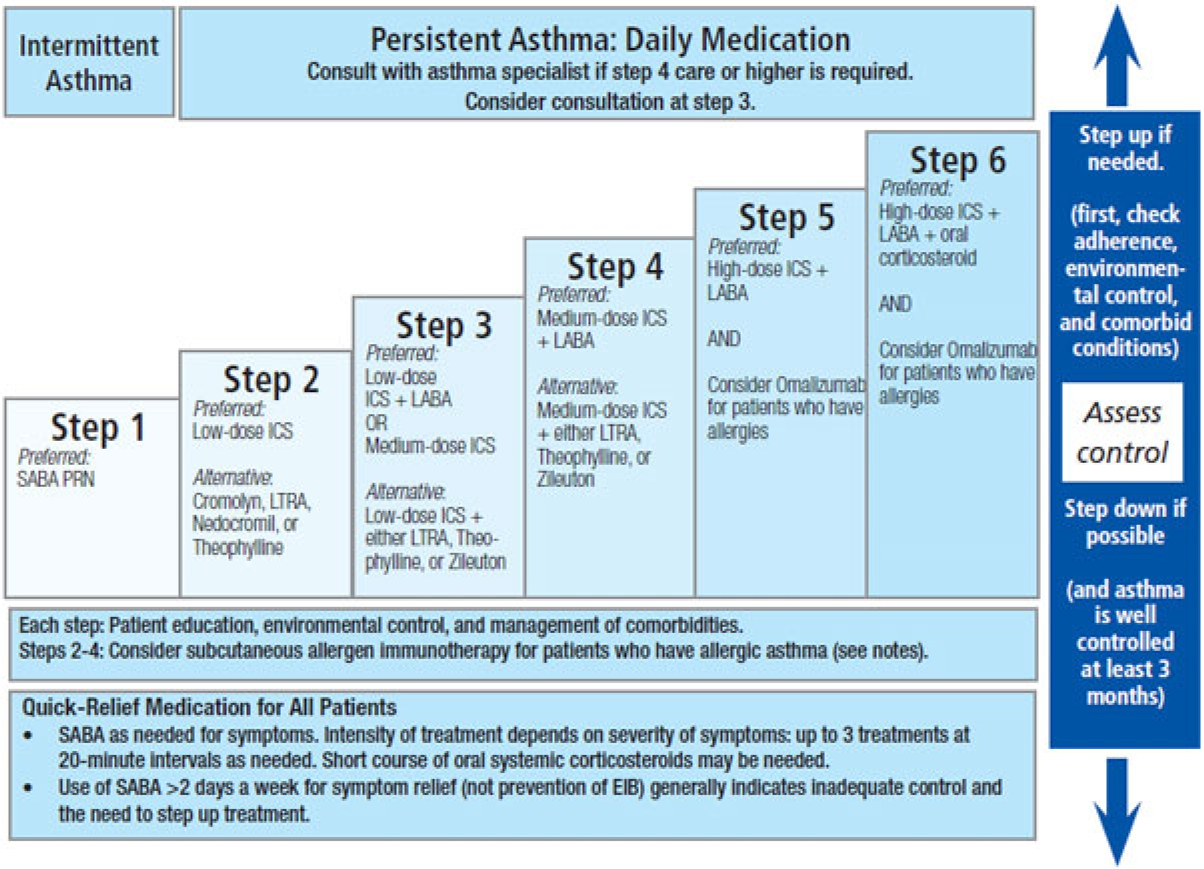

Source: http://www.rtmagazine.com/issues/articles/2009-05_01.asp. *Accessed 31 Mar 2010*. Adapted from: National Institutes of Health. Clinical Practice Guidelines: Expert Panel Report 3: Guidelines for the Diagnosis and Management of Asthma. NHLBI: *NIH publication 08-4051*, 2007.

Table A.2 Asthma Severity Classification for Study Enrollment

ASTHMA SEVERITY GROUP CLASSIFICATION

Controller Medications	FEV : ≥ 80%	FEV: ≥60 & <80%	FEV: ≥50 & <60%	FEV1: <50%
No controllers	MILD	EXCLUDE	EXCLUDE	EXCLUDE
Low to Medium dose ICS alone or in combination with any other controller except OSC and omalizumab	EXCLUDE	MODERATE	EXCLUDE	EXCLUDE
High dose ICS alone or in combination with any other controller including OCS and omalizaumab	EXCLUDE	SEVERE	SEVERE	EXCLUDE

APPENDIX B: NHLBI CLASSIFICATION OF DAILY DOSE LEVELS FOR INHALED CORTICOSTEROIDS FOR YOUTHS ≥ 12 YEARS OF AGE AND ADULTS

Drug	Low Daily Dose Adult	Medium Daily Dose Adult	High Daily Dose Adult
**Beclomethasone HFA**<br>40 or 80 mcg/puff	80–240 mcg	>240–480 mcg	>480 mcg
**Budesonide DPI**<br>90, 180, or 200 mcg/inhalation	180–600 mcg	>600–1,200 mcg	>1,200 mcg
**Flunisolide**<br>250 mcg/puff	500–1,000 mcg	>1,000–2,000 mcg	>2,000 mcg
**Flunisolide HFA**<br>80 mcg/puff	320 mcg	>320–640 mcg	>640 mcg
**Fluticasone**			
**HFA/MDI:** 44, 110, or 220 mcg/puff	88–264 mcg	>264–440 mcg	>440 mcg
**DPI:** 50, 100, or 250 mcg/inhalation	100–300 mcg	>300–500 mcg	>500 mcg
**Mometasone DPI**<br>200 mcg/inhalation	200 mcg	400 mcg	>400 mcg
**Triamcinolone acetonide**<br>75 mcg/puff	300–750 mcg	>750–1,500 mcg	>1,500 mcg

Key: DPI, dry powder inhaler; HFA, hydrofluoroalkane; MDI, metered-dose inhaler

Adapted from: National Institutes of Health. Clinical Practice Guidelines: Expert Panel Report 3: Guidelines for the Diagnosis and Management of Asthma. NHLBI: *NIH publication 08-4051*, 2007.
