# Supplementary material for: Asthma characteristics and biomarkers from the Airways Disease Endotyping for Personalized Therapeutics (ADEPT) longitudinal profiling study
Source: Respir Res. 2015 Nov 17;16:142. doi: 10.1186/s12931-015-0299-y (PMC4650115; doi:10.1186/s12931-015-0299-y)
Supplement: Additional file 2: — Asthma History Questionnaire. (DOCX 29 kb) [file 12931_2015_299_MOESM2_ESM.docx]

| **Additional file 2: Asthma History Questionnaire** | Mild | Moderate | Severe |
| --- | --- | --- | --- |
| **n** | 52 | 55 | 51 |
| **Patient reports premature birth** | 4 (7.7%) | 1 (1.8%) | 5 (9.8%) |
| **First degree (family) relatives have asthma (Y)** | 26 (50.0%) | 23 (43.4%) | 22 (43.1%) |
| **Age of onset of asthma symptoms** | | | |
| Birth-1 year | 3 (5.8%) | 1 (1.8%) | 3 (5.9%) |
| 2-10 years | **22(42.3%)** | 13(24.5%) | 14(27.5%) |
| 11-20 years | 12(23.1%) | 13(24.5%) | 6(11.8%) |
| 21-40 years | 9(17.3%) | **21(39.6%)** | **17(33.3%)** |
| 41-60 years | 6(11.5%) | 7(13.2%) | 11(21.6%) |
| 61-70 years | 0 | 0 | 0 |
| **History of serious lung infection e.g. pneumonia, RSV** | 10 (19.2%) | 20 (36.4%) | 16 (31.4%) |
| **Reported comorbid conditions** | | | |
| Seasonal nasal allergies | 41 (78.8%) | 35 (63.6%) | 32 (62.7%) |
| Eczema | 15 (28.8%) | 12 (21.8%) | 15 (29.4%) |
| Nasal allergies all year round | 18 (34.6%) | 17 (30.9%) | 16 (31.4%) |
| Dermatitis | 9 (17.3%) | 5 (9.1%) | 7 (13.7%) |
| Seasonal eye allergies | 24 (46.2%) | 26 (47.3%) | 22 (43.1%) |
| Gastric reflux (heartburn) | 18 (34.6%) | 18 (32.7%) | 18 (35.3%) |
| Eye allergies all year round | 6 (11.5%) | 4 (7.3%) | 6 (11.8%) |
| Anxiety and/or depression | 14 (26.9%) | 11 (20.0%) | 14 (27.5%) |
| Nasal polyps | 2 (3.8%) | 10 (18.2%) | 15 (29.4%) |
| Sinus infections | 26 (50.0%) | 19 (34.5%) | 24 (47.1%) |
| Obesity | 2 (3.8%) | 4 (7.3%) | 7 (13.7%) |
| Sleep apnea | 1 (1.9%) | 1 (1.8%) | 3 (5.9%) |
| None of the above | 2 (3.8%) | 4 (7.3%) | 1 (2.0%) |
| **Reported symptoms when asthma is worsening** |  |  |  |
| Shortness of breath | 45 (86.5%) | 51 (92.7%) | 50 (98.0%) |
| Red, itchy eyes | 16 (30.8%) | 13 (23.6%) | 12 (23.5%) |
| Tightness in your chest | 38 (73.1%) | 43 (78.2%) | 41 (80.4%) |
| Sneezing or itchy, runny nose | 25 (48.1%) | 27 (49.1%) | 23 (45.1%) |
| Cough | 38 (73.1%) | 40 (72.7%) | 36 (70.6%) |
| Coughing up phlegm | 23 (44.2%) | 33 (60.0%) | 31 (60.8%) |
| Skin rashes e.g. red, itchy welts | 8 (15.4%) | 6 (10.9%) | 6 (11.8%) |
| High-pitched whistling sounds when breathing in and out(wheezing) | 38 (73.1%) | 42 (76.4%) | 42 (82.4%) |
| Other symptoms | 7 (13.5%) | 7 (12.7%) | 3 (5.9%) |
| **History of Nocturnal asthma** | 34 (65.4%) | 45 (81.8%) | 44 (86.3%) |
| **Asthma symptoms related to any of the following:** |  |  |  |
| n | 52 | 55 | 51 |
| Seasonal allergens (e.g. grass, tree, mold) | 35 (67.3%) | 28 (50.9%) | 33 (64.7%) |
| Weather changes | 20 (38.5%) | 31 (56.4%) | 30 (58.8%) |
| House dust (e.g. after vacuuming carpets) | 41 (78.8%) | 41 (74.5%) | 36 (70.6%) |
| Cold air | 32 (61.5%) | 33 (60.0%) | 31 (60.8%) |
| Viral infections (e.g. cough, flu) | 32 (61.5%) | 38 (69.1%) | 28 (54.9%) |
| Symptoms of gastric reflux | 5 (9.6%) | 3 (5.5%) | 6 (11.8%) |
| Stress, anxiety, depression | 14 (26.9%) | 14 (25.5%) | 21 (41.2%) |
| Exercise and other strenuous activities | 41 (78.8%) | 38 (69.1%) | 43 (84.3%) |
| Exposure to damp basements, cottages, etc. | 11 (21.2%) | 24 (43.6%) | 21 (41.2%) |
| Exposure to an agricultural environment | 13 (25.0%) | 10 (18.2%) | 15 (29.4%) |
| Food, food additives, and preservatives (e.g. sulfites) | 4 (7.7%) | 5 (9.1%) | 14 (27.5%) |
| Exposure to pollen during a particular season | 27 (51.9%) | 29 (52.7%) | 27 (52.9%) |
| Irritants (e.g. tobacco smoke, strong odors, air pollutants, chemicals, vapors, gases, aerosols) | 33 (63.5%) | 39 (70.9%) | 40 (78.4%) |
| Taking certain drugs | 3 (5.8%) | 4 (7.3%) | 4 (7.8%) |
| Animals | 21 (40.4%) | 26 (47.3%) | 23 (45.1%) |
| Exposures in the work environment | 8 (15.4%) | 8 (14.5%) | 10 (19.6%) |
| Other triggers | 6 (11.5%) | 3 (5.5%) | 6 (11.8%) |
| **How the patient self-rated their asthma severity** |  |  |  |
| n | 52 | 54 | 51 |
| Mild | 46 (88.5%) | 14 (25.9%) | 7 (13.7%) |
| Moderate | 6 (11.5%) | 39 (72.2%) | 14 (27.5%) |
| Severe | 0 | 1 (1.9%) | 30 (58.8%) |
| **Patient ever smoked tobacco products** |  |  |  |
| n | 52 | 55 | 51 |
| No | 35 (67.3%) | 31 (56.4%) | 31 (60.8%) |
| Yes | 17 (32.7%) | 24 (43.6%) | 20 (39.2%) |
| **The average number of tobacco products smoked per day** |  |  |  |
| n | 16 | 24 | 20 |
| 1-5 | 8 (50.0%) | 9 (37.5%) | 7 (35.0%) |
| 6-10 | 5 (31.3%) | 11 (45.8%) | 9 (45.0%) |
| 11-15 | 2 (12.5%) | 2 (8.3%) | 1 (5.0%) |
| 16-20 | 1 (6.3%) | 2 (8.3%) | 2 (10.0%) |
| >20 | 0 | 0 | 1 (5.0%) |
| **Ever exposed to second-hand tobacco smoke at home** |  |  |  |
| n | 52 | 55 | 51 |
| No | 18 (34.6%) | 15 (27.3%) | 15 (29.4%) |
| Yes | 34 (65.4%) | 40 (72.7%) | 36 (70.6%) |
| **Currently exposed to tobacco smoke at home** |  |  |  |
| n | 34 | 40 | 36 |
| No | 29 (85.3%) | 32 (80.0%) | 31 (86.1%) |
| Yes | 5 (14.7%) | 8 (20.0%) | 5 (13.9%) |
| **Ever exposed to second-hand tobacco smoke in the workplace** |  |  |  |
| n | 52 | 55 | 51 |
| No | 31 (59.6%) | 28 (50.9%) | 18 (35.3%) |
| Yes | 21 (40.4%) | 27 (49.1%) | 33 (64.7%) |
| **Currently exposed to tobacco smoke in workplace** |  |  |  |
| n | 21 | 27 | 33 |
| No | 17 (81.0%) | 21 (77.8%) | 29 (87.9%) |
| Yes | 4 (19.0%) | 6 (22.2%) | 4 (12.1%) |
| **Asthma prescription medications prescribed now or in past** |  |  |  |
| n | 52 | 55 | 51 |
| Short-acting bronchodilators, also known as rescue inhalers or relievers (e.g. albuterol/salbutamol) | 49 (94.2%) | 44 (80.0%) | 49 (96.1%) |
| Daily controller medications (e.g. inhaled corticosteroids, long-acting bronchodilators, leukotriene inhibitors) | 23 (44.2%) | 54 (98.2%) | 48 (94.1%) |
| Oral corticosteroids (e.g. prednisone) on a daily basis | 4 (7.7%) | 9 (16.4%) | 11 (21.6%) |
| Allergen immunotherapy | 5 (9.6%) | 6 (10.9%) | 8 (15.7%) |
| omalizumab | 0 | 0 | 7 (13.7%) |
| Other | 1 (1.9%) | 2 (3.6%) | 1 (2.0%) |
| **Frequency of asthma worsening requiring an increase in medications** |  |  |  |
| n | 52 | 55 | 51 |
| Never | 3 (5.8%) | 3 (5.5%) | 3 (5.9%) |
| Rarely | 22 (42.3%) | 25 (45.5%) | 11 (21.6%) |
| Once every 2-3 years | 3 (5.8%) | 4 (7.3%) | 6 (11.8%) |
| Once a year | 10 (19.2%) | 9 (16.4%) | 5 (9.8%) |
| More than once each year | 10 (19.2%) | 6 (10.9%) | 13 (25.5%) |
| Many times each year | 4 (7.7%) | 8 (14.5%) | 13 (25.5%) |
| **How many times in the past 5 years have you been hospitalized for asthma?** |  |  |  |
| n | 52 | 55 | 51 |
| 0 times | 47 (90.4%) | 46 (83.6%) | 35 (68.6%) |
| 1-3 times | 5 (9.6%) | 9 (16.4%) | 14 (27.5%) |
| 4-7 times | 0 | 0 | 2 (3.9%) |
| **Has your asthma ever caused you to stop breathing?** |  |  |  |
| n | 52 | 55 | 51 |
| No | 46 (88.5%) | 48 (87.3%) | 42 (82.4%) |
| Yes | 6 (11.5%) | 7 (12.7%) | 9 (17.6%) |
| **Has your asthma ever caused you to have a breathing tube inserted in your windpipe?** |  |  |  |
| n | 52 | 55 | 51 |
| No | 52 (100.0%) | 54 (98.2%) | 47 (92.2%) |
| Yes | 0 | 1 (1.8%) | 4 (7.8%) |
| **Has your asthma ever caused you to be admitted into an intensive care unit?** |  |  |  |
| n | 52 | 55 | 51 |
| No | 52 (100.0%) | 48 (87.3%) | 43 (84.3%) |
| Yes | 0 | 7 (12.7%) | 8 (15.7%) |
